# Soft and Transient Bioelectronics Enabled by Polymer‐Free Ti_3_C_2_T*_x_* MXene Hydrogels

**DOI:** 10.1002/adma.75137

**Published:** 2026-09-25

**Authors:** Raghav Garg, Franco A. Laimo, Stefano Ippolito, Prastuti Upadhyay, Spencer R. Averbeck, Dimitris Boufidis, Charles‐Antoine Assenmacher, Dayo O. Adewole, Sai Panigrahy, Bita Soltan Mohammadlou, Lucy Plant, Yuan Zhang, Andrew G. Richardson, Yury Gogotsi, D. Kacy Cullen, Flavia Vitale

**Affiliations:** ^1^ Department of Neurology University of Pennsylvania Philadelphia Pennsylvania USA; ^2^ Center of Neuroengineering and Therapeutics University of Pennsylvania Philadelphia Pennsylvania USA; ^3^ Center for Neurotrauma Neurodegeneration and Restoration Corporal Michael J. Crescenz Veterans Affairs Medical Center Philadelphia Pennsylvania USA; ^4^ Department of Neurosurgery Perelman School of Medicine University of Pennsylvania Philadelphia Pennsylvania USA; ^5^ A.J. Drexel Nanomaterials Institute and Department of Materials Science and Engineering Drexel University Philadelphia Pennsylvania USA; ^6^ Department of Bioengineering University of Pennsylvania Philadelphia Pennsylvania USA; ^7^ Comparative Pathology Core Department of Pathobiology School of Veterinary Medicine University of Pennsylvania Philadelphia Pennsylvania USA; ^8^ Department of Mechanical Engineering and Mechanics Drexel University Philadelphia Pennsylvania USA; ^9^ Department of Physical Medicine and Rehabilitation University of Pennsylvania Philadelphia Pennsylvania USA

**Keywords:** bioelectronics, hydrogel, Mxene, neural interfaces, transient electronics

## Abstract

Implantable bioelectronics for acute neural applications must combine tissue‐like mechanics with high electrochemical performance while enabling safe biodegradation to avoid risks associated with explant surgeries. Here, we present MXgel bioelectronics, a soft and fully transient hydrogel platform that enables high‐fidelity electrophysiological recordings and effective stimulation with programmable degradation under physiological conditions. The platform employs polymer‐free Ti_3_C_2_T*_x_* MXene hydrogels (MXgel) that achieve exceptional electrical conductivities (hydrated: 790 ± 150 S m^−1^, freeze‐dried: 4420 ± 576 S m^−1^). Integrated on biodegradable gelatin‐based substrates, MXgel bioelectronics exhibit low electrochemical impedance (1.06 ± 0.37 kΩ at 1 kHz), 25‐fold higher cathodic charge storage (207.1 ± 48.8 mC cm^−2^), and 10‐fold greater charge injection capacity (0.24 ± 0.08 mC cm^−2^) than conventional clinical electrodes. We demonstrate that these properties enable cortical and peripheral neural recordings with ∼60 dB signal‐to‐noise ratio and effective neuromodulation. Moreover, MXgel bioelectronics undergo safe degradation under physiological conditions with predictable and tunable kinetics. Importantly, MXgel bioelectronics do not trigger severe foreign body reaction, and their degradation byproducts do not accumulate in the body, confirming safe systemic clearance. Together, these results establish MXgel bioelectronics as a clinically relevant platform for soft, high‐performance, and transient neural interfaces.

## Graphical abstract

We present Ti_3_C_2_T*_x_* MXene hydrogel (MXgel) bioelectronics, a soft, flexible, and fully transient platform for high‐fidelity neural recording and stimulation. MXgel bioelectronics exhibit exceptional electrical conductivities, low electrochemical impedances, and high charge injection capacities. This enables neural recordings with ∼60 dB signal‐to‐noise ratio and precise neuromodulation. Moreover, MXgel bioelectronics undergo safe biodegradation with programmable kinetics without triggering severe foreign body reaction.

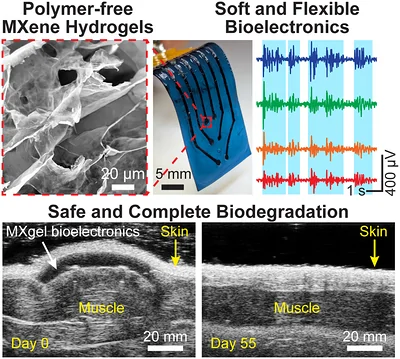

## 1 Introduction

Implantable bioelectronics interface with excitable biological tissues and allow direct electrophysiological recordings and functional control via electrical stimulation [1, 2, 3]. While electrically active tissues are inherently soft with intricate topographies (*E*_Neural tissue_ ∼ 10 kPa), conventional bioelectronics are rigid with significantly greater mechanical moduli since they are composed of metals and semiconductors (e.g., *E*_Pt_ and *E*_Si_ > 1 GPa) [2, 4, 5]. This mechanical mismatch at the interface initiates a stiffness‐related foreign body reaction (FBR) [6], leading to the formation of fibrotic encapsulation that reduces device functionality [7, 8]. Conductive hydrogels have emerged as promising materials for soft bioelectronics due to their unique biomimetic mechanics, high water content, and versatile functional properties [3, 4, 9, 10]. Hydrogel‐based bioelectronics lead to minimal stiffness‐related FBR, making them attractive for chronic applications where long‐term stability and seamless bio‐integration are essential [11, 12, 13]. However, for acute applications (such as epilepsy monitoring, temporary cardiac pacing, or post‐injury neuromonitoring), the use of hydrogel bioelectronics presents a significant bottleneck since secondary surgeries to explant the devices can increase the risk of infection, inflammation, and tissue damage [13]. Transient bioelectronics eliminate the need for device extraction by safely degrading in a physiological environment [13, 14]. Despite their utility in acute applications, transient bioelectronics rely on bioresorbable metals (e.g., Mg, Zn, Mo, W) [12] and semiconductors (e.g., Si), which are inherently rigid, have limited electrochemical functionality, and exhibit unpredictable degradation kinetics [13, 15, 16]. Moreover, the growing demand for disposable medical electronics further exacerbates the global challenge of medical waste due to their hazardous processing and environmental persistence [17]. This requires a shift toward sustainable materials and fabrication strategies that minimize the ecological footprint of next‐generation medical devices. Therefore, to address these challenges, there is a critical need to develop implantable bioelectronic platforms that can form mechanically compliant biointerfaces with reliable recording and stimulation capabilities, while ensuring predictable and safe in vivo biodegradation.

Here, we introduce a soft and transient hydrogel bioelectronic platform that integrates high‐performance conductive hydrogels with predictable, controllable, and safe biodegradation. Our approach leverages the unique properties and favorable biocompatibility of Ti_3_C_2_T*_x_* MXene to bridge the gap between soft and transient electronics [18, 19, 20]. Unlike conventional conductive hydrogel fillers (e.g., metallic nanoparticles [21], nanocarbons [22, 23], and conductive polymers [24, 25]) that are biologically persistent, Ti_3_C_2_T*_x_* undergoes oxidation‐driven hydrolysis in physiological environments to form biocompatible byproducts [26, 27, 28]. To harness this transience of Ti_3_C_2_T*_x_* for soft bioelectronics, we formulated MXene hydrogels (MXgels) composed of ionically crosslinked Ti_3_C_2_T*_x_*. The resulting high‐porosity MXgels do not require a polymeric scaffold, leading to superior electrical conductivities, thus, outperforming conventional conductive hydrogels. We engineer a fully transient platform by incorporating MXgel onto a biodegradable genipin‐crosslinked gelatin (gelapin) substrate, ensuring both mechanical compliance and safe biodegradation of the entire device. Crucially, we show that we can precisely program the degradation rates of MXgel bioelectronics under physiological conditions by tuning the morphology of Ti_3_C_2_T*_x_* flakes and the composition of the gelapin substrate. Comprehensive electrochemical characterization reveals that MXgel bioelectronics demonstrate lower electrochemical impedance and greater cathodic charge storage and charge injection capacities than clinical metal electrodes. These properties enable high‐fidelity neural recordings with signal‐to‐noise ratio (*SNR*) up to 60 dB and effective stimulation in the central and peripheral nervous systems. Finally, we establish that MXgel bioelectronics are biocompatible and do not trigger severe FBR. The degradation byproducts also do not accumulate in the body, thus confirming safe systemic clearance. Our work establishes MXgel bioelectronics as a versatile platform for clinically viable, soft, and fully transient bioelectronics that pave the way for sustainable medical devices.

## 2 Results

### 2.1 Synthesis and Properties of MXgels

We synthesize MXgel by directly crosslinking individual Ti_3_C_2_T*_x_* flakes using cations, specifically Ca^2+^ ions (Figure 1a). The wet‐chemical etching‐based synthesis of Ti_3_C_2_T*_x_* MXene yields negatively charged Ti_3_C_2_T*_x_* flakes with ‐F, ‐Cl, = O, and ─OH terminations [29]. Addition of cations, under specific concentration ratios, to a stable colloidal dispersion of Ti_3_C_2_T*_x_* initiates electrostatic interactions between the two species, leading to rapid gelation [30, 31]. Although a variety of cations can be used for the gelation of Ti_3_C_2_T*_x_*, we selected Ca^2+^ ions due to their high biocompatibility and ubiquitous presence in biological systems. To synthesize Ti_3_C_2_T*_x_* MXgels, we mixed equal volumes of 40 mg mL^−1^ Ti_3_C_2_T*_x_* dispersion with 100 mM CaCl_2_ at a mass ratio of 10:1 (Ti_3_C_2_T*_x_*:Ca^2+^ ions, Note S1). This resulted in a highly porous, interconnected 3D network of Ti_3_C_2_T*_x_* MXene flakes evident upon freeze‐drying (Figure 1b), with uniform distribution of Ca^2+^ ions (Figure S1 and Table S1). As anticipated, the adsorption of Ca^2+^ ions (charge density = +52 C mm^−3^) [32] onto the surface of Ti_3_C_2_T*_x_* flakes leads to an increase in the zeta potential from −40.6 ± 1.7 mV to −34.8 ± 0.6 mV (Figure 1c). Crucially, Raman spectroscopy confirmed that Ca^2+^‐induced rapid gelation of Ti_3_C_2_T*_x_* does not affect the physical and chemical structure of Ti_3_C_2_T*_x_* flakes (Figure S2) [33]. Moreover, approximately 3.70 ppm and 7.01 ppm of Ca^2+^ ions are released from MXgels after 5 min and 24 h soak in deionized water (DI‐H_2_O) at 37°C, respectively; while 2.50 ppm and 12.50 ppm Ca^2+^ ions are released from MXgels after 5 min and 24 h soak in artificial cerebrospinal fluid (aCSF) at 37°C, respectively (Figure S3). These results indicate that the interactions between Ti_3_C_2_T*_x_* and Ca^2+^ ions remain stable even under physiological conditions. Importantly, the concentration of Ca^2+^ ions released from MXgel is substantially lower than the concentration of free Ca^2+^ in human plasma [34].

**FIGURE 1:**
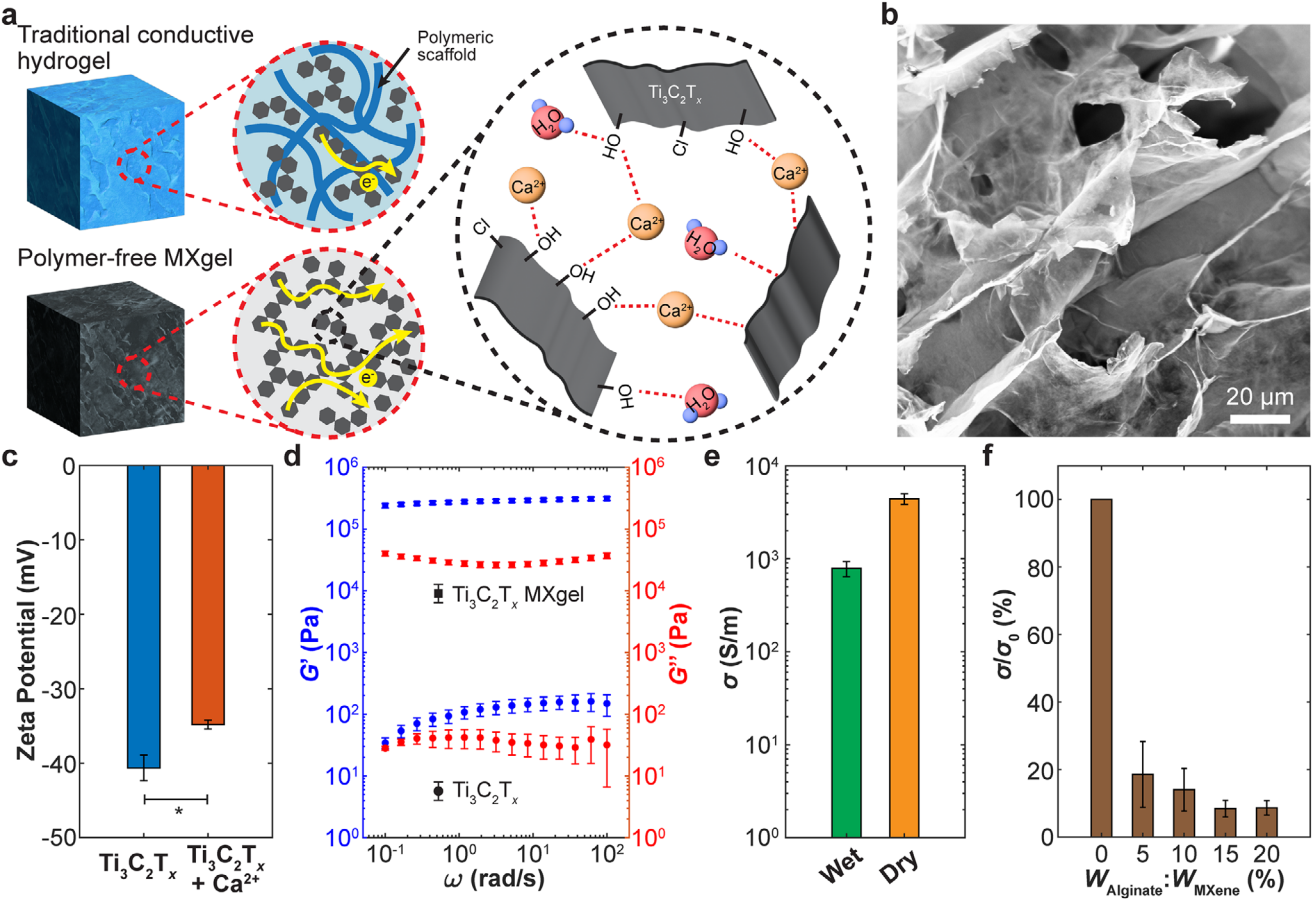
Structural and electrical properties of MXgel. (a) Schematic of a traditional conductive hydrogel with polymeric scaffold and conductive fillers, and a polymer‐free MXgel composed entirely of Ti_3_C_2_T*_x_* flakes. Red dashed insets illustrate the hydrogel network and electron transport networks. Black dashed inset illustrates the crosslinking of Ti_3_C_2_T*_x_* flakes via Ca^2+^ ions. (b) Scanning electron microscopy image of freeze‐dried Ti_3_C_2_T*_x_* MXgel. (c) Zeta potential of precursor Ti_3_C_2_T*_x_* dispersion and Ti_3_C_2_T*_x_* with Ca^2+^ ions. Data presented as mean ± SD (*N* = 3). *p < 0.05, one‐way ANOVA with post‐hoc Tukey. (d) Storage (*G*′) and loss (*G*″) moduli as a function of angular frequency (*ω*) at 0.1% strain for precursor Ti_3_C_2_T*_x_* dispersions and Ti_3_C_2_T*_x_* MXgels. Data presented as mean ± SD (*N* = 3). (e) Electrical conductivity (*σ*) of wet (hydrogel) and dry (freeze‐dried) Ti_3_C_2_T*_x_* MXgel. Data presented as mean ± SD (*N* = 5). (f) Relative conductivity (*σ/σ*_0_) as a function of the weight of alginate (*W*_Alginate_) to the weight of MXene (*W*_MXene_) in the alginate‐MXgels. Data presented as mean ± SD (*N* = 5).

To elucidate the effect of gelation on mechanical properties, we performed dynamic rheological measurements of the storage (*G*′) and loss moduli (*G*″) on MXgel and precursor Ti_3_C_2_T*_x_* aqueous dispersions (40 mg mL^−1^). We observed that *G*′_MXgel_ was frequency‐independent and greater than *G*″_MXgel_, indicating the formation of large domain structures similar to those in a solid‐like gel (Figure 1d) [35, 36]. Due to the high concentration of Ti_3_C_2_T*_x_* in the dispersions, we observed *G′*_MXene_ > *G″*_MXene_ between 10^0^–10^2^ Hz [35]. However, the fact that *G*′_MXgel_ was approximately three orders of magnitude higher than *G*′_MXene_ (*G*′_MXgel_ ∼ 3 × 10^5^ Pa and *G*′_MXene_ ∼ 1 × 10^2^ Pa at 1 Hz, Figure 1d), corroborates the successful formation of MXene hydrogels [35]. Similar trends in rheological properties were noted as a function of oscillatory stress (Figure S4a). We determined that the tan δ (tan δ = *G*″/*G*′) values for MXgel were lower than those for precursor Ti_3_C_2_T*_x_* dispersion, confirming the more elastic nature of the structure achieved upon gelation (tan δ_MXgel_ ∼ 10^−1^ and tan δ_MXene_ ∼ 3 × 10^−1^ at 1 Hz, Figure S4b) [37]. Importantly, the Young's modulus of hydrated MXgel was measured to be 5.74 ± 1.09 kPa via atomic force microscope (AFM)‐based nanoindentation measurements (Figure S5a). In addition, we calculated the shear yield stress of hydrated MXgel to be 2.1 ± 0.4 kPa (Figure S5b). These intrinsic values confirm that the active electrode material directly interfacing with the tissue closely matches the modulus of neural tissue.

We characterized the electrical conductivity of MXgels using four‐point measurements. In the fully hydrated state, MXgels exhibit electrical conductivity of 787 ± 147 S m^−1^ (Figure 1e). Such high electrical conductivity can be attributed to the inherently high electrical conductivity of Ti_3_C_2_T*_x_* (up to 20,000 S cm^−1^) [38] and suggests strong crosslinking of adjacent MXene flakes, allowing efficient electron transport despite the ubiquitous presence of H_2_O molecules within the network. Increasing the concentration of Ca^2+^ crosslinker to 1000 mM significantly increased the electrical conductivity of MXgel to 1357 ± 368 S m^−1^ (Figure S6). However, excessively high concentrations of Ca^2+^ ions in a biological environment substantially increase the risk of ion leaching, which can be hazardous and cytotoxic to surrounding tissues [39]. Therefore, we selected 100 mM Ca^2+^ ions as the optimum formulation as it results in robust gelation and functional electrical conductivity, while maximizing physiological safety and preventing harmful leaching. Removal of H_2_O via freeze‐drying increases inter‐flake contact significantly increasing electrical conductivity [40]. In the case of MXgel, freeze‐drying forms aerogels with electrical conductivity of 4420 ± 576 S m^−1^ (Figure 1e). Notably, the electrical conductivity of hydrated polymer‐free MXgels surpasses that of conventional hydrogels composed of conductive fillers such as nanocarbons (*σ* ∼ 1–40 S m^−1^) [22] and MXene/PEDOT hybrids (*σ* ∼ 3.85 × 10^2^ S m^−1^) [41], and it is comparable to that of conductive polymer‐based hydrogels (*σ* ∼ 0.6–4.7 × 10^3^ S m^−1^) [42, 43, 44]. To demonstrate the hindering effect of passive polymeric scaffolds on electron transport, we introduced alginate into MXgels. We observed that adding as little as 5% (*w*/*w*) of alginate results in a ∼81.5% reduction in electrical conductivity (Figure 1f). This can be attributed to the disruption of the percolation network by the insulating polymer chains.

### 2.2 Design and Electrochemical Performance of MXgel Bioelectronics

To realize tissue‐like transient bioelectronics, we optimized a hydrogel substrate onto which the conductive MXgel electrodes and interconnects could be patterned. We identified genipin‐crosslinked gelatin (gelapin) as the ideal substrate, owing to its well‐documented biocompatibility, mechanical compliance with biological tissues, low inherent electrical conductivity, and biodegradability [45, 46, 47]. We selected 100% crosslinked gelapin as the substrate as it exhibits mechanical moduli similar to soft biological tissues (*E*_100%_ = 218.9 ± 36.8 kPa) and minimal swelling under physiological conditions (Figures S7 and S8). Crucially, the electrical conductivity of gelapin substrates (*σ* = 4.59 ± 0.93 mS m^−1^) is five orders of magnitude lower than that of MXgels and two orders of magnitude lower than that of biological tissues (*σ* = 0.1–1 S m^−1^) [48] (Figure S9).

Prior to fabricating bioelectronic devices, we confirmed the lack of cytotoxic effects induced by Ti_3_C_2_T*_x_* MXgels and gelapin hydrogels via the alamarBlue metabolic assay on primary rat astrocytes. The relative fluorescence of resorufin measured from both MXgel and gelapin extracts was greater than that of the control (blank extract) and Triton X‐100‐treated extracts (positive control), indicating an absence of toxic leachables and suggesting retention of elevated metabolic activity upon exposure to MXgel and gelapin extracts (Figure S10a) [49, 50, 51]. This is further corroborated by the linear increase in relative fluorescence of resorufin over the incubation period (*R^2^* ≥ 0.97, Figure S10b). We also validate the biocompatibility of our hydrogel formulations via a live/dead assay using primary rat astrocytes. Upon exposure to control (blank), MXgel, and gelapin extracts for 24 h, high astrocyte viabilities of 97.87 ± 1.27%, 97.10 ± 2.79%, and 99.49 ± 0.04%, respectively, were observed (Figures S11a,b). At the same time, high cell densities of 124 ± 13, 134 ± 10, and 127 ± 12 cells mm^−2^ after 24 h exposure to control (blank), MXgel, and gelapin extracts, respectively, were also noted (Figure S11c). The absence of statistical difference in astrocyte viabilities and cell densities between control, MXgel, and gelapin indicates that our hydrogels are biocompatible. Taken together, these new data corroborate previous reports on the biocompatibility and absence of cytotoxicity of Ti_3_C_2_T*_x_* MXene and gelapin [19, 20, 45, 52, 53, 54].

To assemble MXgel devices, we developed a simple molding‐based fabrication scheme (Figure S12). Briefly, we first designed a custom 3D printed master mold and used it to fabricate elastomer‐based negative molds. We then cast gelapin into the elastomer molds and let the gelapin substrate cure overnight into robust hydrogel substrates with a thickness of 416 ± 21 µm. To prevent diffusion of ionic species through gelapin [55], we introduced a thin layer of poly(D, L‐lactide‐*co*‐glycolide) (PLGA_75:25_, lactide:glycolide of 75:25), with a thickness of 17 ± 3 µm, ensuring electrical insulation. We then injected Ti_3_C_2_T*_x_* dispersions (40 mg mL^−1^) into the electrode and interconnect channels, followed by CaCl_2_ solution (100 mM). Once MXgel electrodes were successfully formed, a top passivation layer of PLGA_75:25_ was applied. This process yields soft, flexible devices that retain the highly porous 3D structure of MXgel throughout the fabrication process (Figure 2a–c). The absence of crosstalk between individual electrodes corroborated the integrity of the PLGA passivation and ensured electrical isolation of individual electrodes (Figure 2d).

**FIGURE 2:**
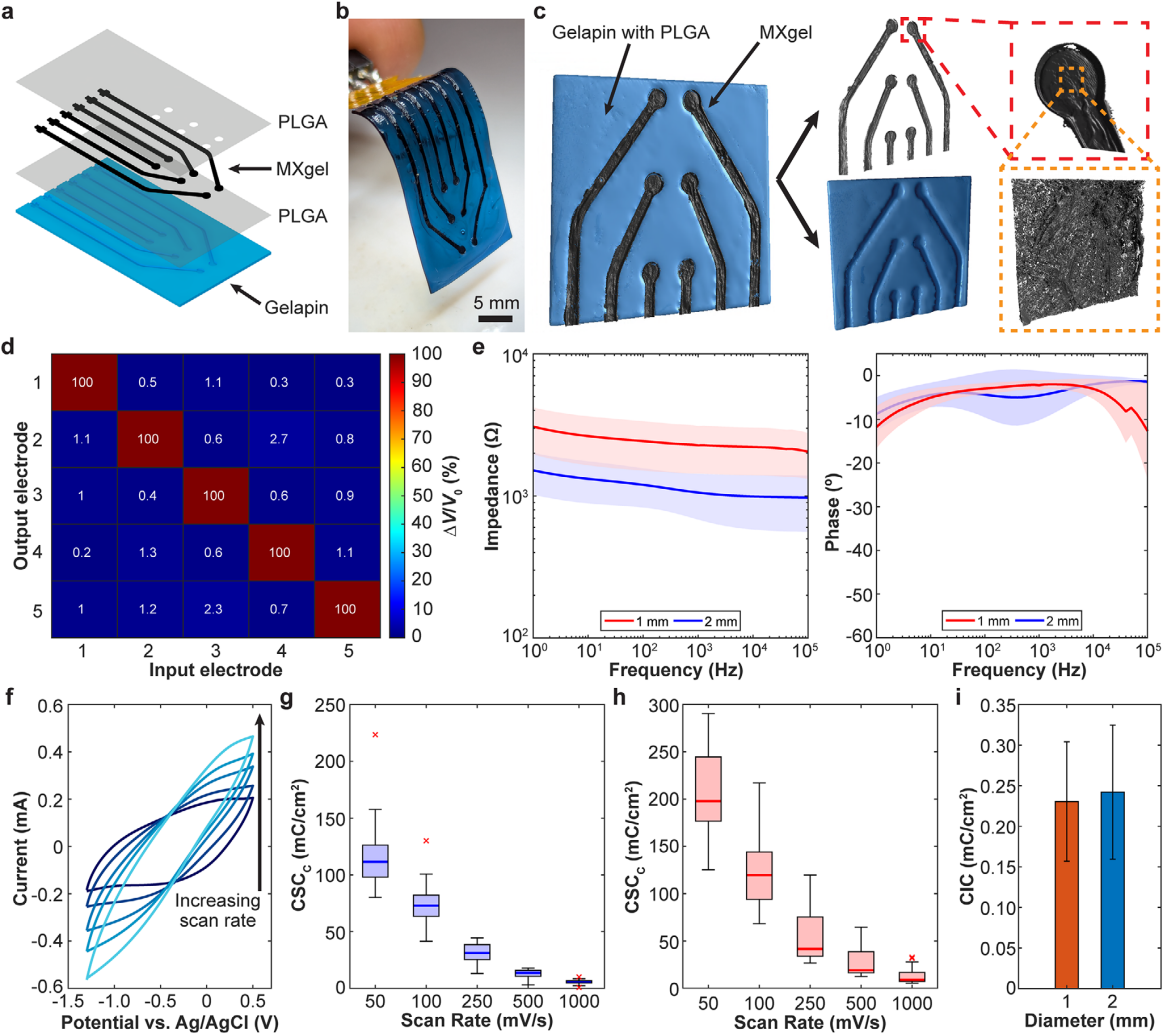
Electrochemical characterization of MXgel bioelectronics. (a) Schematic illustrating the structure of functional MXgel bioelectronics. (b) Optical image of a fabricated MXgel device with 1 mm electrodes. (c) Micro‐CT image reconstruction of a MXgel device showing 3D topography. (d) Crosstalk matrix for a representative MXgel device with five electrodes (*N* = 3 devices). (e) Impedance (left) and phase (right) as a function of frequency as measured for 1 and 2 mm MXgel electrodes. Solid line and shaded region denote mean and standard deviation, respectively (*N* = 17 electrodes across five devices for 1 mm; *N* = 20 electrodes across seven devices for 2 mm). (f) Representative cyclic voltammetry curves of a 2 mm MXgel electrode measured at increasing scan rates of 50, 100, 250, 500, and 1000 mV s^−1^. Cathodic charge storage capacity (*CSC*_C_) as a function of scan rate as measured for (g) 2 mm and (h) 1 mm MXgel electrodes (*N* = 17 electrodes across five devices for 1 mm; *N* = 20 electrodes across seven devices for 2 mm). Central mark, bottom edge, top edge, whiskers, and ‘*x*′ denote median, 25th percentile, 75th percentile, most extreme data points, and outliers, respectively. (i) Charge injection capacity (*CIC*) of MXgel electrodes as a function of electrode diameter. Data presented as mean ± SD (*N* = 14 electrodes across four devices for 1 mm; *N* = 13 electrodes across six devices for 2 mm).

Under physiological conditions (hydrated state), the MXgel bioelectronics exhibit an effective Young's modulus of 324.8 ± 134.0 kPa (Figure S13a) and an effective bending modulus of 339.1 ± 65.8 kPa (Figure S13b). The effective moduli of MXgel bioelectronics are highly compliant with biological tissues, especially when compared to conventional platforms. Here, the optimized multi‐layer structure of MXgel bioelectronics provides the structural integrity necessary for surgical handling and deployment, while simultaneously allowing the MXgel to seamlessly interface with target tissues. Furthermore, subjecting MXgel bioelectronic structures to repeated bending cycles established their mechanical robustness since no delamination of MXgel from the substrate was observed. Crucially, the electrical properties remained stable even after 1000 bending cycles under 20% compressive strain (Figure S14), suggesting excellent functional durability under physiologically relevant strains.

We then performed comprehensive electrochemical characterization of MXgel bioelectronics by evaluating their impedance, charge storage capacity, and charge injection capacity [56, 57]. For high *SNR* electrophysiology recordings, low impedance electrodes are critical [58, 59, 60, 61]. MXgel electrodes exhibit low electrochemical impedances across a wide range of frequencies with average impedances of 2.27 ± 0.83 kΩ and 1.06 ± 0.37 kΩ at 1 kHz for 1 and 2 mm electrodes, respectively (Figure 2e). In the 1–10^5^ Hz frequency regime, the impedance spectra of MXgel electrodes are predominantly resistive, which is attributed to the solution resistance (*R*_S_ ≈ 72 Ω), the size of the electrodes, and the resistance of the connecting cables [58]. The high electrical conductivity and electrochemically active surface area of Ti_3_C_2_T*_x_* further contribute to the low impedance of the electrodes [56].

Ideal stimulation electrodes should exhibit capacitive behavior to maintain a stable electrode‐tissue interface by avoiding oxidation of biochemicals and electrolysis of water [57]. The cyclic voltammetry (CV) response of MXgel electrodes is predominantly capacitive with no faradaic reactions (Figure 2f), owing to the high capacitance of Ti_3_C_2_T*_x_* [62]. Consistent with prior reports [63], the maximum cathodic (*E*_mc_) and anodic potential (*E*_ma_) excursions for MXgel electrodes are ‐1.3 and +0.5 V, respectively. Consequently, the cathodic charge storage capacity (*CSC*_C_) of MXgel electrodes is 207.1 ± 48.8 mC cm^−2^ and 119.0 ± 32.0 mC cm^−2^ for 1 and 2 mm electrodes, respectively (Figure 2g,h, scan rate 50 mV s^−1^), which is up to 25*x* higher than conventional metal thin‐film electrodes and up to 50*x* higher than nanocarbon‐based hydrogel electrodes [22, 64, 65]. Interestingly, the *CSC*_C_ of MXgel electrodes increases with reducing CV scan rates, a phenomenon that can be attributed to the longer duration of the charging–discharging cycles at lower scan rates, allowing greater ionic diffusion through the bulk porosity of the electrodes [57, 66]. Next, we determined the charge injection capacity (*CIC*) of MXgel electrodes by measuring voltage transients under incrementally increasing current pulses. Since the magnitude of *E*_mc_ (‐1.3 V) is smaller than *E*_ma_ (+0.5 V) for Ti_3_C_2_T*_x_*, we designed asymmetric biphasic current pulses with cathodic, inter‐pulse, and anodic pulse durations fixed at 500, 200, and 2000 µs, respectively. This allowed us to maintain charge neutrality at the working electrode by setting the amplitude of the anodic pulses to 0.25*x* that of the cathodic pulses [57]. The *CIC* of 1 and 2 mm MXgel electrodes was determined to be 0.23 ± 0.07 and 0.24 ± 0.08 mC cm^−2^, respectively (Figure 2i), which is up to 10*x* greater than conventional thin‐film Au electrodes [65]. Moreover, under continuous application of physiologically relevant biphasic current pulses with a cathodic phase of 1 mA – 500 µs (Note S2), we did not observe any accelerated electrochemical degradation of the MXgel bioelectronics (Figure S15). An initial drop in electrode impedance was observed; however, this is attributed to the gradual electrolyte infiltration of the porous hydrogel network. Subsequently, there was no significant change in electrode impedance up to 10,000 continuous current pulses (Figure S15). This corroborates robust electrochemical performance and absence of degradation due to electrochemical fatigue.

For applications requiring micro‐scale electrodes, we reduced the diameter of the electrodes to 500 µm while retaining low electrochemical impedances (|*Z*| = 2.74 ± 0.76 kΩ at 1 kHz) and high charge injection capacities (*CIC* = 0.68 ± 0.04 mC cm^−2^) (Figures S16 and S17).

We also validated the compatibility of MXgel bioelectronics with ethylene oxide (EtO) sterilization, a widely adopted technique for sterilizing implantable medical devices [67]. We note that EtO sterilization did not alter the electrochemical impedance of the devices (Figure S18). This observation is in agreement with our prior findings that EtO sterilization does not cause any physical, chemical, or functional changes to Ti_3_C_2_T*_x_* bioelectronics [68]. These results highlight the customizability and readiness of MXgel bioelectronics for their use in sterile implants.

### 2.3 Degradation Kinetics and Programmable Transience

The introduction of foreign materials inside a host triggers its innate immune response [6, 13, 69], which leads to the recruitment of macrophages that generate reactive oxygen species [13]. We leverage this oxidative environment to drive safe biodegradation of MXgel bioelectronics by carefully engineering the structural and chemical properties of our platform. To ensure clinical viability, we investigated both structural and functional lifetimes of MXgel devices. Ti_3_C_2_T*_x_* undergoes hydrolysis and oxidation in the presence of water to form TiO_2_, CO_2_, and hydrocarbons (Figure 3a) [26, 27, 28]. Concurrently, PLGA hydrolyzes into lactic acid and glycolic acid [70], while crosslinked gelatin degrades into gelatin fragments, amines, and organic molecules [45]. All resulting byproducts, including TiO_2_, have been established to be biocompatible [70, 71, 72].

**FIGURE 3:**
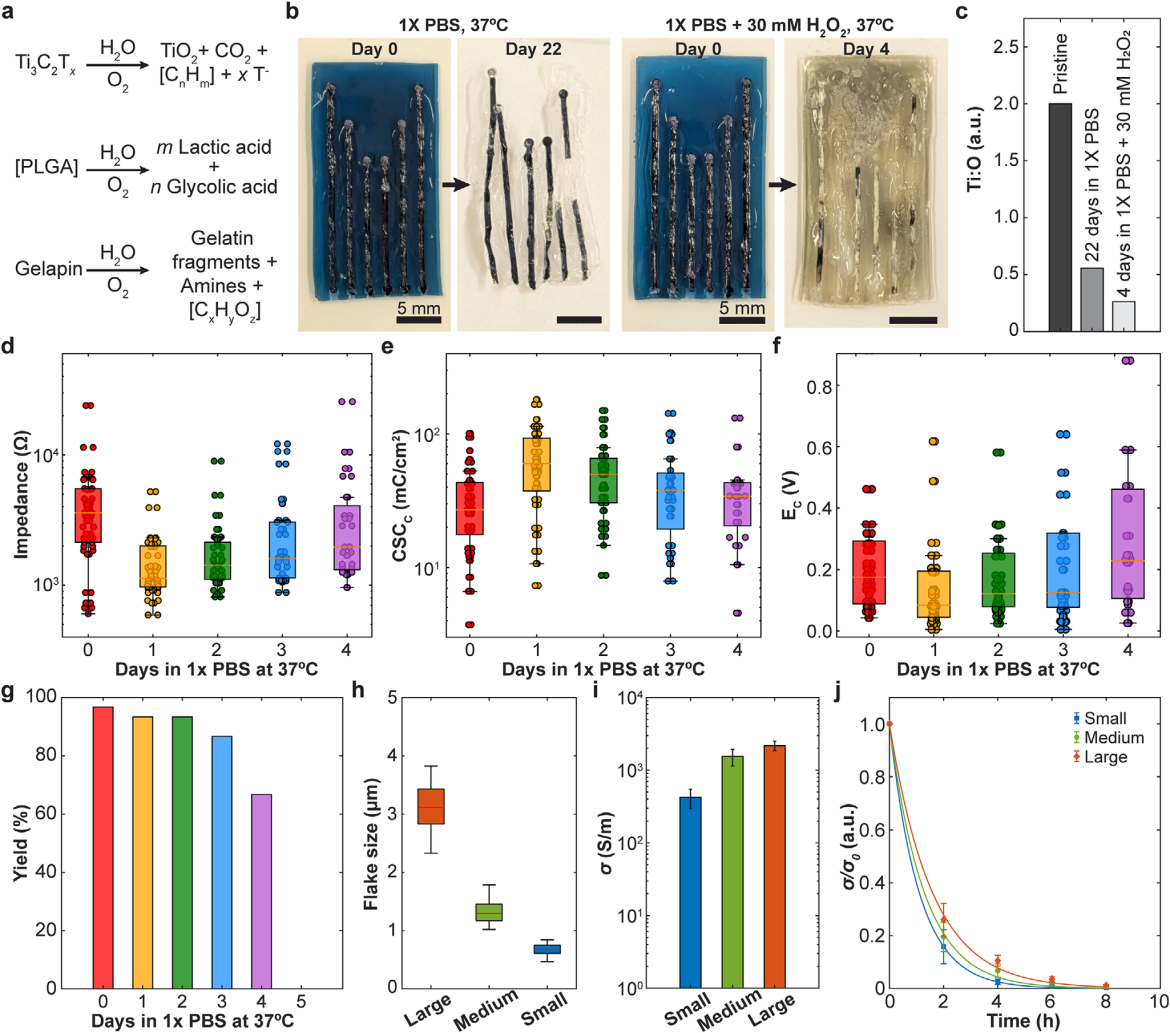
Characterization of the structural and functional degradation of MXgel bioelectronics. (a) Chemical formulas of the hydrolysis of Ti_3_C_2_T*_x_*, PLGA, and gelapin in the presence of H_2_O and O_2_. (b) Optical images of representative MXgel devices over multiple days in 1X PBS and 1X PBS with 30 mM H_2_O_2_ at 37°C (*N* = 5 devices). (c) Ratio of Ti:O for MXgels before and after exposure to 1X PBS and 1X PBS with 30 mM H_2_O_2_. (d) Electrochemical impedance at 1 kHz, (e) *CSC*_C_ at 250 mV s^−1^, and (f) cathodic potential excursion (*E*_c_) for 0.5 mA 0.5‐ms pulses measured for functional 1 mm MXgel electrodes as a function of days in 1X PBS at 37°C. Central mark, bottom edge, top edge, whiskers, and circles denote median, 25th percentile, 75th percentile, one SD, and individual data points, respectively (*N* = 30 electrodes across five devices on day 0). (g) Yield of functional 1 mm MXgel electrodes as a function of days in 1X PBS at 37°C. (h) Size distribution of small, medium, and large flake Ti_3_C_2_T*_x_* (*N* = 60). Central mark, bottom edge, top edge, and whiskers denote median, 25th percentile, 75th percentile, and most extreme data points, respectively. (i) Electrical conductivity (*σ*) of MXgel fabricated with small, medium, and large flake Ti_3_C_2_T*_x_*. (j) Relative electrical conductivity (*σ/σ*_0_) of MXgel fabricated with small, medium, and large flake Ti_3_C_2_T*_x_* as a function of time in 1X PBS with 30 mM H_2_O_2_. Data presented as mean ± SD (*N* = 5).

We assessed the structural lifetime of the devices by exposing them to simulated physiological (1X PBS at 37°C) and inflammation‐mimicking (1X PBS with 30 mM H_2_O_2_ at 37°C) conditions over multiple days. The addition of 30 mM H_2_O_2_ mimics the reactive oxygen species‐rich environment generated around the implant as a result of FBR [56, 69, 73]. Under physiological conditions, substantial dissolution of both the gelapin substrate and exposed Ti_3_C_2_T*_x_* MXene was observed after 22 days (Figure 3b). A reduction in the Ti:O ratio from 2.0 to 0.6 was measured via energy‐dispersive x‐ray spectroscopy (EDS), indicating the formation of TiO_2_ (Figures 3c, S19a, and Table S2). In contrast, inflammation‐mimicking conditions triggered rapid breakdown of gelapin and extensive degradation of Ti_3_C_2_T*_x_* within 4 days, evidenced by a further drop in the Ti:O ratio to 0.3 (Figures 3b,c, S19b, and Table S2). We anticipate that the in vivo structural degradation kinetics will fall within the window defined by these two simulated conditions.

To evaluate the functional lifetime, we monitored the electrochemical performance under simulated physiological conditions. A majority of MXgel electrodes (>50%) retained their low electrochemical impedances, high *CSC*_C_, and stable cathodic overpotential (*E*_c_) over 4 days (Figures 3d–f and S20). A drop in the impedance from 3.6 × 10^3^ Ω (median |*Z*| at 1 kHz) to 1.1 × 10^3^ Ω (median |*Z*| at 1 kHz) was observed within the first 24 h (Figures 3d and S20a), which can be attributed to the increase in electrode capacitance due to gradual electrolyte infiltration of the porous hydrogel network. Notably, no measurable swelling of the MXgel was observed in the first 24–48 h (Figure S21), which is crucial as it minimizes delamination between MXgel, PLGA, and gelapin. The increase in electrode capacitance is well corroborated by the increase in the capacitive behavior of cyclic voltammograms, evident from the increase in *CSC*_C_ from 62 mC cm^−2^ (day 0, median *CSC*_C_ at 250 mV s^−1^) to 136 mC cm^−2^ (day 1, median *CSC*_C_ at 250 mV s^−1^), and reduction in *E*_c_ from 0.16 V (day 0, median *E*_c_) to 0.08 V (day 1, median *E*_c_) (Figures 3e,f and S20b–d). Over time, MXgel electrodes exhibited impedance spectra and faradaic cyclic voltammograms characteristic of the gold backend connectors (Figure S22). At this stage, we determined the MXgel electrodes to have “failed” since the electrochemical interface was no longer strictly restricted to the exposed electrode surface. For MXgel bioelectronics fabricated with 50 mg mL^−1^ PLGA_75:25_, more than 50% of the electrodes were functional for up to 4 days with the device yield gradually declining from 97% to 67% over the first 4 days. By day 5, all MXgel electrodes showed the same electrochemical response as the backend gold connectors and were considered to have failed (Figures 3g and S22). This suggests that the functional failure of MXgel bioelectronics is governed by the gradual permeation of electrolyte into the passivation layers.

Subsequently, we engineered the thickness and composition of the PLGA layer to extend the functional lifetime of the MXgel bioelectronics. We fabricated MXgel bioelectronics with 100 mg ml^−1^ PLGA_75:25_, which resulted in a 28 ± 3 µm thick PLGA layer. The thicker PLGA passivation drastically extended the functional lifetime of MXgel bioelectronics with more than 50% electrode remaining functional for up to 13 days under physiological conditions (Figure S23a,c). In parallel, we also fabricated devices with 50 mg mL^−1^ PLGA_85:15_ (lactide:glycolide, 85:15), resulting in a 18 ± 3 µm thick PLGA layer. Here, more than 50% of the electrodes were functional for up to 8 days under physiological conditions (Figure S23b,c). The extended stability of PLGA_85:15_ can be attributed to the higher lactide content, which increases the polymer's hydrophobicity and subsequently slows down the hydrolytic degradation rate [74]. We also note that the devices maintained their structural integrity over multiple days without any visual evidence of delamination of MXgel from the underlying substrates (Figure S24).

A unique advantage of using Ti_3_C_2_T*_x_* is that its physical and chemical structure can be easily tailored to control the functional and structural degradation kinetics [29, 75, 76]. To demonstrate this, we synthesized Ti_3_C_2_T*_x_* flakes of different sizes: small (0.67 ± 0.09 µm), medium (1.32 ± 0.19 µm), and large (3.12 ± 0.39 µm) (Figures 3h and S25). The electrical conductivity of MXgel fabricated with small, medium, and large Ti_3_C_2_T*_x_* flakes was 421 ± 125, 1540 ± 394, and 2180 ± 314 S m^−1^, respectively (Figure 3i). This increase in electrical conductivity can be attributed to improved electron transport and reduced inter‐flake resistance as the flake size increases [77]. We next evaluated the degradation kinetics of MXgel synthesized using different Ti_3_C_2_T*_x_* flake sizes by tracking their electrical conductivities under accelerated conditions of 1X PBS with 30 mM H_2_O_2_. MXgel fabricated with small, medium, and large Ti_3_C_2_T*_x_* flakes experienced exponential degradation of their electrical conductivities with time constants of 1.09, 1.27, and 1.57 h, respectively (Figure 3j and Table S3). The rapid decay in electrical conductivity of MXgel fabricated with small Ti_3_C_2_T*_x_* flakes can be attributed to the higher density of defects and edges that increase the material's susceptibility to hydrolytic and oxidative degradation [76, 78]. On the other hand, the slower degradation of MXgel fabricated with large Ti_3_C_2_T*_x_* flakes can be attributed to the lower density of defects. Therefore, by tailoring the structure of Ti_3_C_2_T*_x_* and the passivation layers, the functional lifetime of MXgel bioelectronics can be tuned to meet diverse clinical applications such as epilepsy monitoring, ICU monitoring, and temporary nerve stimulation.

### 2.4 High‐Fidelity Cortical Recordings and Neuromodulation

Building on the systemic structural and functional evaluation, we validated MXgel bioelectronics for recording and stimulating the central nervous system. First, we acquired electrocorticography (ECoG) recordings from the somatosensory barrel field cortex (S1‐bf) of an anesthetized rodent using a grid of four 1 mm MXgel electrodes (Figure 4a,b). The low impedance of MXgel electrodes minimizes noise (e.g., thermal noise) [79], allowing us to record ECoG signals with high *SNR* (59.1 ± 9.8 dB). This performance is consistent with emerging high‐surface‐area materials (e.g., Pt black, CNTs) designed to outperform standard planar Au and Pt interfaces [80]. Such high signal quality renders MXgel bioelectronics well‐suited for capturing critical high‐frequency oscillations, including high gamma activity (80–150 Hz), which require high‐fidelity interfaces.

**FIGURE 4:**
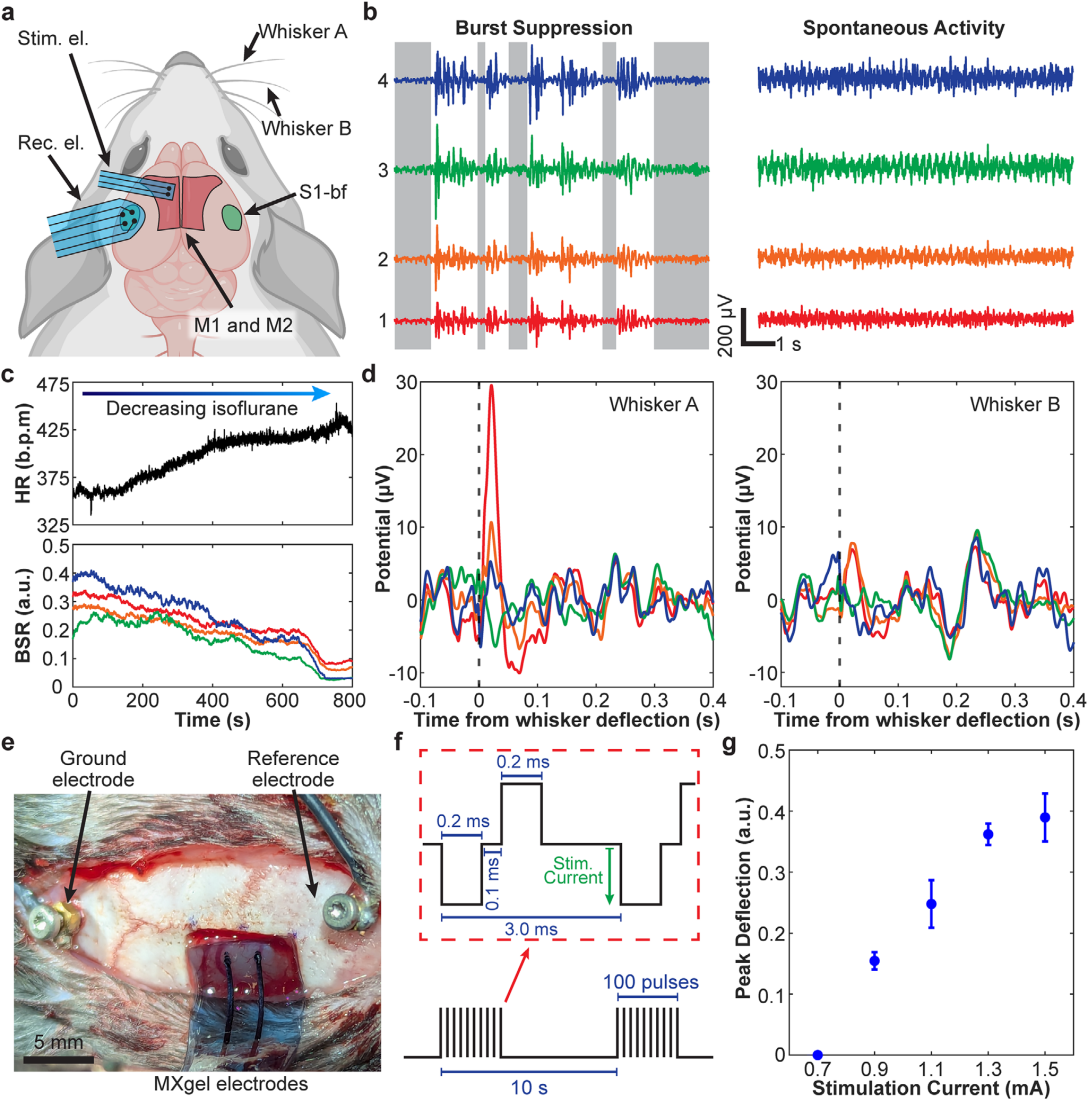
MXgel bioelectronics for ECoG recordings and supracortical stimulation. (a) Schematic depicting the placement of recording electrodes (Rec. el.) over the somatosensory cortex‐ barrel field (S1‐bf) and stimulation electrode (Stim. el.) over the primary motor cortex (M1 and M2) in a rat. (b) Representative electrocorticography (ECoG) recordings from S1‐bf in a rat demonstrating burst suppression (left) and spontaneous activity (right). (c) Heart rate (*HR*) and burst suppression ratio (*BSR*) as calculated from ECoG recordings from the S1‐bf rat as the concentration of isoflurane is decreased over time. (d) Average evoked ECoG activity recorded over the sensory cortex of a rat upon physical deflection of whisker A (*N* = 273 deflections) and whisker B (*N* = 550 deflections). (e) Photograph of MXgel electrodes over the motor cortex of a rat with reference and ground electrodes. (f) Illustration of the stimulation protocol applied over the motor cortex as a train of 100 biphasic pulses. (g) Peak deflection of the jaw as a function of the amplitude of the stimulation current applied over the motor cortex. Data presented as mean ± SD (*N* = 10 pulses per stimulation current amplitude).

Next, we varied the dose of the isoflurane anesthesia and recorded its effect on cortical activity. Increasing the dose of isoflurane induced characteristic suppression of intermittent cortical activity (a.k.a. burst suppression), while reducing the isoflurane dose restored continuous spontaneous activity (Figure 4b) [81]. We quantified the burst suppression ratio (*BSR*) across the array and observed spatial variability of 20%–40% that reflects heterogeneity in cortical silencing (Figure 4c) [82]. We further assessed the spatial selectivity by recording evoked potentials in response to mechanical stimulation of multiple whiskers. Across the four‐electrode grid, varying amplitudes of evoked cortical activity were recorded when different whiskers were stimulated. Evoked potentials were recorded in somatotypically aligned electrodes upon mechanical stimulation of Whisker A; whereas no evoked potentials were recorded in non‐aligned electrodes upon mechanical stimulation of Whisker B (Figure 4d).

Direct modulation of cortical activity through supracortical stimulation enables precise neuromodulation without disrupting the integrity of the cortex [83]. Therefore, we tested the potential of MXgel electrodes for supracortical stimulation by placing 500 µm electrodes over the motor cortex (Figure 4e). Trains of symmetric biphasic pulses with amplitudes ranging from 0.7 to 1.5 mA, cathodic/anodic pulse duration of 0.2‐ms, and inter‐pulse duration of 0.1‐ms were delivered (Figure 4f). We observed that currents exceeding 0.7 mA elicited a stimulation‐evoked deflection of the rodent's jaw. Increasing the amplitude of the stimulation current increased the magnitude of the deflection (Figure 4g). These results confirm the potential of MXgel bioelectronics for effectively recording and stimulating cortical activity.

### 2.5 Recording and Stimulation in the Peripheral Nervous System

The peripheral nervous system (PNS) is composed of nerves that reside outside the central nervous system and innervate muscles and organs. Conventional bioelectronic interfaces can deform or damage these soft nerves given the mechanical mismatch. Hydrogel‐based electronics address this challenge by establishing a more compliant interface that minimizes tissue disruption. To evaluate the performance of MXgel bioelectronics as PNS interfaces, we implanted 1 mm MXgel electrodes over the rat *sciatic nerve* to deliver electrical stimulation and over the *tibialis anterior* muscle to record evoked responses (Figure 5a). As a benchmark for the recorded evoked activity, we inserted a needle electrode into the *tibialis anterior* muscle belly. Direct electrical stimulation of the *sciatic nerve* using cathodic‐first current pulses with increasing current amplitudes (0.47–1.07 mA) and 0.2‐ms pulse duration resulted in robust activation of the *tibialis anterior* muscle as illustrated by the characteristic evoked electromyography (EMG) (Figure 5b). The MXgel electrodes reliably captured evoked EMG across 10 stimulation trials, with minimal inter‐trial variability. The superficial placement of the MXgel electrode led to surface EMG recordings with waveforms different than those acquired with the needle electrode, which was inserted into the muscle belly. This is further corroborated by the difference in the magnitude of peak‐to‐peak potential (*V*_pp_) of the evoked EMG, which varied between 6.8 ± 0.2 mV for MXgel and 20.0 ± 0.1 mV for the needle electrodes under 1.07 mA pulses (Figure 5c). Despite the differences in electrode positions, the recruitment curves as recorded by MXgel and needle electrodes followed characteristic sigmoidal curves with onset at ∼ 0.57 mA.

**FIGURE 5:**
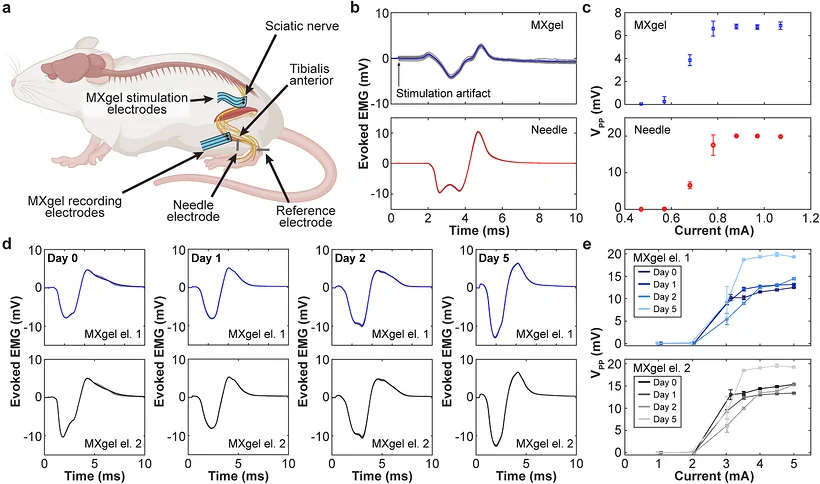
PNS stimulation and evoked EMG recording using MXgel bioelectronics. (a) Schematic depicting the implantation of MXgel recording and stimulation electrodes over the *tibialis anterior* and the *sciatic nerve*, respectively. (b) Evoked EMG recorded at the *tibialis anterior* using 1 mm MXgel and a needle electrode when the *sciatic nerve* is stimulated with a 1.07 mA symmetric cathodic‐first current pulse via bipolar 1 mm MXgel electrodes. Gray and colored plots denote the individual pulses (*N* = 10) and average of individual traces, respectively. (c) Peak‐to‐peak potential as a function of stimulation current pulse amplitude. Data presented as mean ± SD (*N* = 10 pulses per stimulation current amplitude). (d) Evoked EMG recorded at the *tibialis anterior* using two implanted 1 mm MXgel electrodes under transcutaneous stimulation of the *sciatic nerve* with 5.0 mA pulses. Gray and colored plots denote the individual pulses (*N* = 10) and the average of individual traces, respectively. (e) Peak‐to‐peak potential (*V*_pp_) as a function of transcutaneous stimulation current pulse amplitude. Dark to light colors denote responses recorded from day 0 to day 5. Data presented as mean ± SD (*N* = 10 pulses per stimulation current amplitude).

Through our extensive simulated degradation tests, we established that the maximum functional lifetime of MXgel bioelectronics is 5 days. Therefore, we implanted two 1 mm MXgel electrodes on the *tibialis anterior* muscle and recorded evoked EMG under transcutaneous *sciatic nerve* stimulation over a period of 5 days (Figure S26). We were able to successfully record evoked EMG using the implanted MXgel electrodes over 5 days, indicating reliable functionality (Figure 5d,e). The maximum *V*_pp_ recorded under 5 mA stimulation pulses was 12.5 ± 0.1 mV (day 0), 13.2 ± 0.1 mV (day 1), 14.5 ± 0.2 mV (day 2), and 19.3 ± 0.1 mV (day 5) for MXgel electrode 1; and 15.4 ± 0.1 mV (day 0), 13.4 ± 0.1 mV (day 1), 15.3 ± 0.3 mV (day 2), and 19.2 ± 0.2 mV (day 5) for MXgel electrode 2 (Figure 5e). The variations in the evoked EMG over multiple days can be attributed to the minor differences in the placement of the transcutaneous stimulator over the *sciatic nerve*. However, all recruitment curves followed a sigmoidal function with a consistent onset current amplitude of 2 mA. These results were consistent in a second animal where evoked EMG was reliably recorded using MXgel electrodes over a period of 4 days (Figure S27).

### 2.6 In Vivo Biocompatibility of MXgel Bioelectronics

The clinical utility of transient bioelectronics hinges on their ability to undergo complete biodegradation and systemic clearance without eliciting chronic tissue inflammation or long‐term toxicity. Therefore, we performed extensive multimodal analysis of the long‐term in vivo biodegradation, systemic clearance, and local tissue tolerance of implanted MXgel bioelectronics over an 8‐week period. We first utilized high‐resolution ultrasound imaging to serially monitor the physical dimensions of the devices implanted over the *biceps femoris* muscle for up to 8 weeks (Figures 6a, S28, and S29). Over the 8‐week time period, we observed a steady reduction in device thickness from 0.68 ± 0.06 mm (at 0 weeks) to 0.15 ± 0.06 mm (at 8 weeks), corroborating the progressive in vivo biodegradation of our MXgel bioelectronics (Figure 6b). Throughout the 8 weeks, the animals displayed steady weight gain (Figure 6c), suggesting that neither the devices nor their degradation products caused systemic toxicity or physiological distress.

**FIGURE 6:**
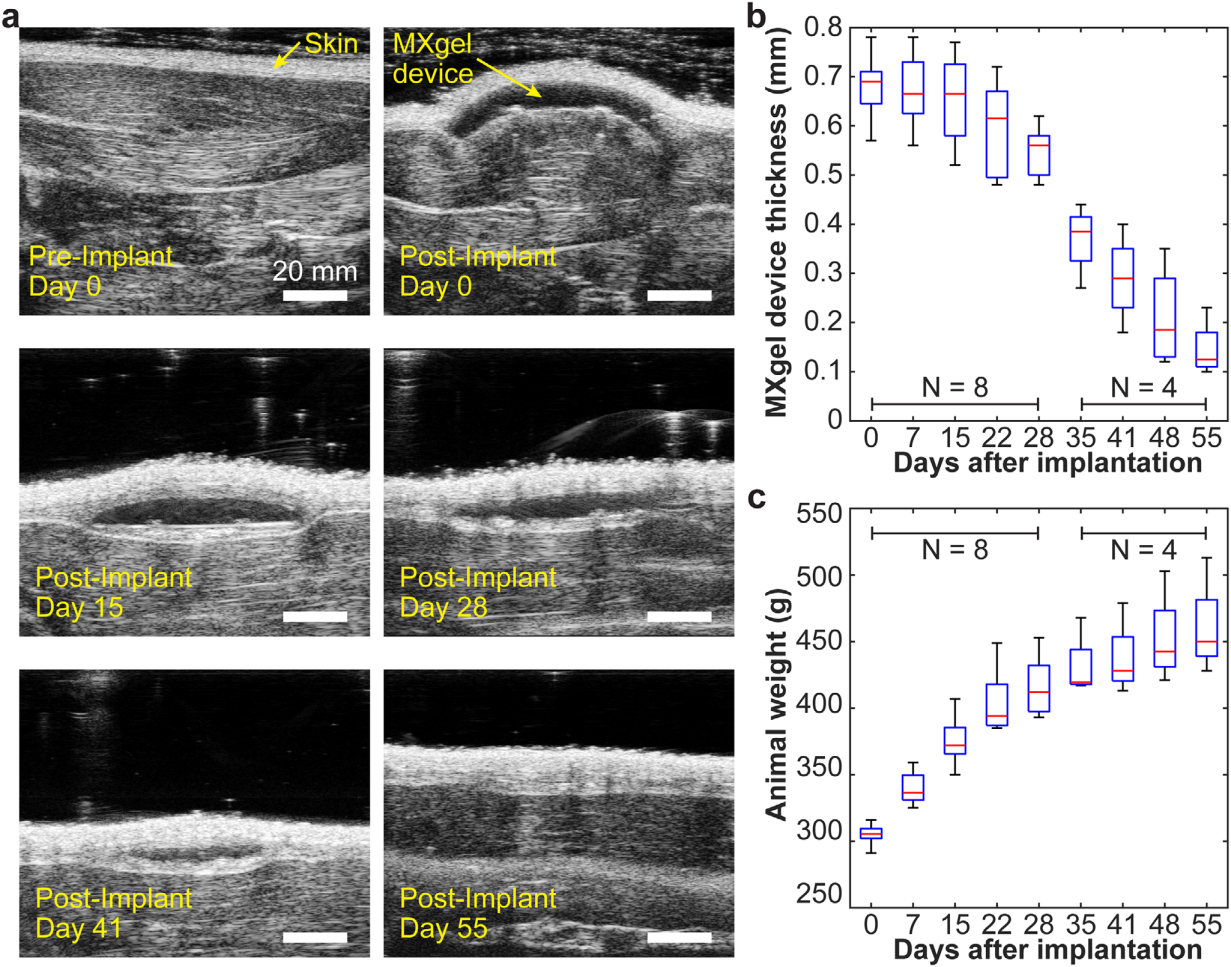
In vivo structural evaluation of MXgel bioelectronic devices. (a) Representative ultrasound images of the *biceps femoris* before and after implantation of MXgel bioelectronics. (b) Thickness of MXgel bioelectronic devices implanted over the *biceps femoris* as a function of number of days after implantation. Central mark, bottom edge, top edge, and whiskers denote median, 25th percentile, 75th percentile, and most extreme data points, respectively (*N =* 8 animals on day 0). (c) Weight of the animals (rat) as a function of number of days after MXgel bioelectronic device implantation. Central mark, bottom edge, top edge, and whiskers denote median, 25th percentile, 75th percentile, and most extreme data points, respectively (*N =* 8 animals on day 0).

Next, we investigated any potential accumulation of the degradation byproducts of Ti_3_C_2_T*_x_*, specifically TiO_2_, by measuring the concentrations of Ti^4+^ ions in the liver, kidneys, and spleen at 4 and 8 weeks post‐implantation. At 4 weeks, no Ti^4+^ ions were detected in any of the analyzed tissue samples. At 8 weeks, only trace levels of Ti^4+^ ions were detected in the liver (0.02 ± 0.01 ppm), kidneys (0.02 ± 0.01 ppm), and spleen (0.01 ± 0.01 ppm) (Table S4). These negligible concentrations of Ti^4+^ ions confirm that the degradation byproducts of Ti_3_C_2_T*_x_* do not accumulate in the body and are safely excreted via regular clearance pathways.

Finally, we conducted extensive long‐term histopathological analysis using hematoxylin and eosin (H&E) staining of the MXgel bioelectronics’ implant site at the *biceps femoris* to assess tissue response over 8 weeks. Compared to the contralateral (control) sections, the tissue architecture at the ipsilateral (implanted) sections was well preserved, which included well‐organized muscle fibers and the absence of any morphological disruptions (Figure 7a). Subsequently, tissue inflammation, fibrosis, muscle injury, and hydrogel persistence were assessed by a trained veterinary pathologist using a semi‐quantitative pathological scoring scheme [84]. At 4 weeks post‐implantation, mild granulomatous inflammation was observed on the ipsilateral side (inflammation score = 2; none/unremarkable = 0 and severe = 4), which resolved to minimal granulomatous inflammation by 8 weeks (inflammation score = 1) (Table S5). A mild‐to‐moderate fibrotic capsule on both the inner (muscle‐facing) and outer (skin‐facing) sides of the implant was also observed with a fibrosis score of 2.75 ± 0.5 and 3.0 at 4 and 8 weeks, respectively (Table S5). Specifically, the thickness of the inner capsule, including the underlying fascia, was 235 ± 129 µm and 279 ± 182 µm at 4 and 8 weeks, respectively; while that of the outer capsule was 131 ± 67 µm and 174 ± 118 µm at 4 and 8 weeks, respectively (Figure 7c). In the contralateral sections, the average thickness of the fascia was measured as 171 ± 64 µm and 113 ± 54 µm at 4 and 8 weeks, respectively (Figure 7c).

**FIGURE 7:**
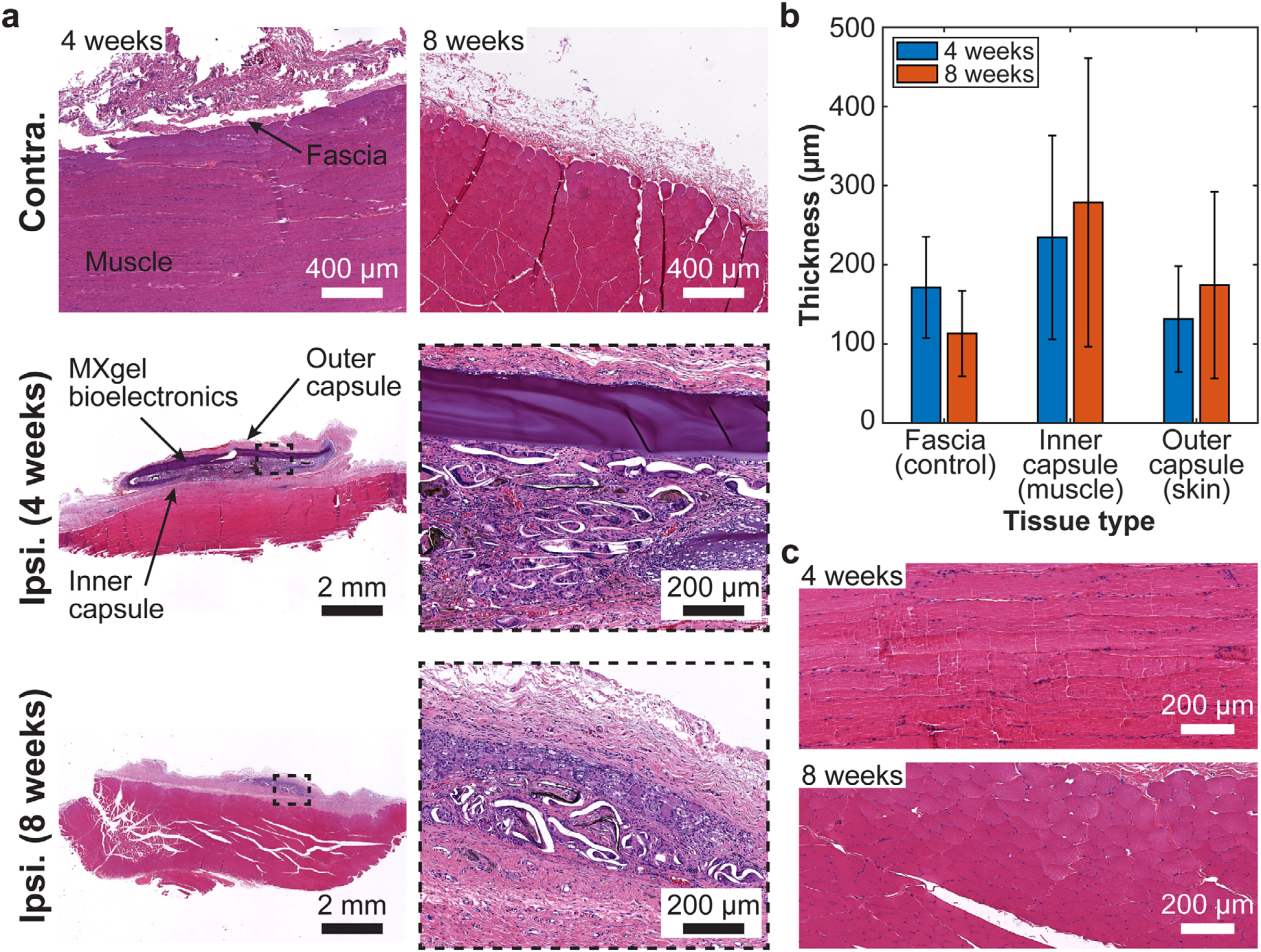
Histopathological structural evaluation of implanted MXgel bioelectronic devices. (a) Representative hematoxylin and eosin (H&E) stained histological images of *biceps femoris* cross‐sections following the implantation of MXgel bioelectronics for 4 and 8 weeks. Ipsilateral (Ipsi.) side contained the hydrogel bioelectronic implant, whereas contralateral (Contra.) side served as the non‐implanted internal negative control. (b) Thickness of the fascia (contralateral control), inner capsule (muscle side), and outer capsule (skin side) as measured from H&E stained photomicrographs of *biceps femoris* cross‐sections following implantation of MXgel bioelectronics at 4 and 8 weeks. Data presented as mean ± SD (*N =* 4). (c) Representative H&E stained high‐resolution images of *biceps femoris* cross‐sections following the implantation of MXgel bioelectronics for 4 and 8 weeks.

To quantify the overall tissue response, we defined an FBR score as the sum of the inflammation and fibrosis scores. Average FBR scores of 4.75 ± 0.50 and 4.00 ± 0.00 at 4 and 8 weeks, respectively, suggest a mild FBR to the implanted MXgel bioelectronics (no FBR = 0 and severe FBR = 7–8). This is consistent with the documented tissue response to the surgical implantation of foreign materials and bioelectronic devices [7, 69]. Importantly, no chronic inflammation or material‐induced toxicity was observed, and the underlying muscle tissue architecture remained well preserved throughout the 8‐week implantation period (Figure 7c and Table S5). Concurrently, the hydrogel persistence score decreased from 3 at 4 weeks to 1 at 8 weeks (Table S6), providing direct histological evidence of the near‐complete biodegradation of the implanted MXgel bioelectronics, consistent with the findings from the in vivo ultrasound (Figures 6 and S28 and S29). Together, these findings establish that MXgel bioelectronics undergo predictable, complete biodegradation without triggering severe FBR or systemic bioaccumulation.

## 3 Conclusion

In this work, we introduce MXgel bioelectronics as unique soft yet transient hydrogel bioelectronics for acute electrophysiological recording and stimulation. MXgel is fabricated through simple crosslinking of Ti_3_C_2_T*_x_* MXene with Ca^2+^ ions, resulting in a polymer‐free conductive hydrogel with exceptional electrical conductivities up to 790 ± 150 and 4420 ± 576 S m^−1^ in hydrated and freeze‐dried states, respectively. Subsequently, MXgel bioelectronics exhibit low electrochemical impedances (|*Z*| = 1.06 ± 0.37 kΩ at 1 kHz) and high stimulation capacities (*CSC*_C_ = 207.1 ± 48.8 mC cm^−2^ and *CIC* = 0.24 ± 0.08 mC cm^−2^). These favorable functional properties allow high‐fidelity electrophysiological recordings with *SNR* up to 60 dB and effective delivery of electrical stimulation for neuromodulation. Moreover, we demonstrate that MXgel bioelectronics can fully dissolve under physiological conditions, offering safe and effective alternatives to permanent hydrogel bioelectronics that necessitate surgical extraction and generate medical waste. Crucially, we establish that MXgel bioelectronics are biocompatible and do not trigger severe FBR. The subsequent degradation byproducts of MXgel bioelectronics also do not accumulate in the body and are safely excreted. Thus, achieving performance comparable to or exceeding reported thin‐film based transient bioelectronics and hydrogel‐based bioelectronics (Table S7).

Despite these advances, the current MXgel bioelectronics rely on wired physical connections to external recording amplifiers and pulse generators, limiting their applicability as fully implantable neural interfaces. Future iterations of this platform will necessitate integration of wireless communication and power transfer to minimize risks associated with interconnect failure or infection [85, 86], which can be achieved via inductive coupling [87]. Ultimately, by combining tissue‐like softness, high performance, and programmable degradation, MXgel bioelectronics pave the way for the next generation of sustainable neural interfaces.

## 4 Experimental Section

### 4.1 Material Synthesis

#### 4.1.1 Ti_3_C_2_T*_x_* MXgel Fabrication

Ti_3_C_2_T*_x_* MXene was produced by Murata Manufacturing Co. Ltd. (Japan), following the minimally intensive layer delamination (MILD) method [88]. Here, gentle mechanical shaking was used instead of the sonication processes to obtain larger Ti_3_C_2_T*_x_* flakes with a minimal concentration of defects. The obtained surfactant‐free Ti_3_C_2_T*_x_* MXene colloidal dispersions in DI‐H_2_O were diluted to a concentration of 40 mg mL^−1^ for fabricating the hydrogels. The average size of Ti_3_C_2_T*_x_* MXene flakes was measured to be 1.67 ± 0.20 µm. To prepare Ti_3_C_2_T*_x_* MXgels, a set volume of 40 mg mL^−1^ Ti_3_C_2_T*_x_* MXene dispersions were placed in appropriate molds or vials. An equal volume of 100 mM CaCl_2_ was then added on top of the Ti_3_C_2_T*_x_* MXene to achieve a mass ratio of 10:1 (Note S1). Gelation was subsequently allowed to take place for 5 min at room temperature.

To prepare MXgels with greater concentrations of Ca^2+^ ions, equal volumes of either 500 mM or 1000 mM CaCl_2_ were added to 40 mg mL^−1^ Ti_3_C_2_T*_x_* MXene. Gelation was subsequently allowed to take place for 5 min at room temperature.

#### 4.1.2 MXene and Alginate Hydrogel Synthesis

To prepare Ti_3_C_2_T*_x_* MXene and alginate hydrogels, stock solutions of Ti_3_C_2_T*_x_* and alginate (Sigma‐Aldrich, W201502) were prepared in DI‐H_2_O with concentrations of 56 and 25 mg mL^−1^, respectively. Fixed volumes of Ti_3_C_2_T*_x_* dispersion were aliquoted in individual microcentrifuge tubes, and alginate solution was added to achieve final weight ratios of 0, 5, 10, 15, and 20 between solid alginate (*W*_Alginate_) and Ti_3_C_2_T*_x_* (*W*_MXene_). Each microcentrifuge tube was gently vortexed to create a homogeneous dispersion without breaking the Ti_3_C_2_T*_x_* flakes. The dispersions were subsequently cast into 2 × 15 × 0.50 mm molds and crosslinked with 100 mM CaCl_2_ for 5 min. The crosslinked hydrogels were gently washed three times with DI‐H_2_O to remove excess ions.

#### 4.1.3 Gelapin Synthesis

Prior to gelapin synthesis, the concentration of amine groups in gelatin was measured using the ninhydrin assay (3.67 × 10^−4^ moles of amine per 1 g of gelatin). Gelatin solution was then prepared by adding 1 g of gelatin to a glass vial with 5 mL DI‐H_2_O and 5 mL glycerol (Sigma‐Aldrich, G9012). The gelatin was allowed to bloom for 15 min at room temperature and later placed in an oven set to 60°C until the gelatin was fully dissolved. A 50 mg mL^−1^ stock solution of genipin was prepared by dissolving 20 mg genipin into a solution of 20 mL DI‐H_2_O and 20 mL ethanol (Supelco, EX0276‐1). The genipin solution was gently vortexed for 30 s. Since the amine concentration of gelatin was measured to be 3.67 × 10^−4^ moles g^−1^, appropriate volumes of the genipin solution were added to the gelatin solution to create 0%, 25%, 50%, and 100% crosslinked samples. The two solutions were gently vortexed at low speeds for 15 s and then placed in an oven set to 80°C for 4 min. The final solution was then cast into the desired molds and allowed to gel overnight at room temperature.

#### 4.1.4 Synthesis of Ti_3_C_2_T*_x_* MXgels with Different Flake Sizes

A stock Ti_3_C_2_T*_x_* dispersion was prepared by aqueous etching of Ti_3_AlC_2_ MAX phase using 18 mL HCl and 2 mL HF per gram of MAX phase at 35°C for 24 h. The resulting multilayer MXene was washed with DI‐H_2_O and intercalated with LiCl and then washed and collected using a centrifuge. The single‐layer MXene colloid was split into three equal parts. One part was used as is for large‐flake MXene; one part was sonicated for 10 min, resulting in medium‐size MXene flakes; and the final part was sonicated for 15 min, resulting in small‐size MXene flakes. The three samples were separately concentrated to 40 mg mL^−1^ by centrifuging at 10,000 rpm for 30 min. Subsequently, Ti_3_C_2_T*_x_* MXgels with different flake sizes were prepared using a 100 mM CaCl_2_ solution following the procedure described above.

### 4.2 Material Characterization

#### 4.2.1 Scanning Electron Microscopy (SEM) Imaging and Energy Dispersive X‐Ray Spectroscopy (EDS)

SEM and EDS characterizations were performed using a Supra 50 VP field‐emission SEM (Zeiss) with a 40 Ultim Max EDS detector (Oxford Instruments). Sample preparation for SEM and EDS required freeze‐drying the hydrogels. More specifically, the samples were first frozen at −20°C for 2 days and then freeze‐dried overnight using a freeze‐dryer (Labconco Freezone 2.5 plus Freeze Dryer) set to −85°C and 0.10 mbar. The samples were then loaded inside the SEM, and high‐resolution images were acquired using an accelerating voltage of 10 kV with a working distance of 5–15 mm. For EDS mapping, the working distance was set to 15 mm. As the dry samples were conductive, no sputter coating was needed. To measure the flake size of Ti_3_C_2_T*_x_*, dispersions of Ti_3_C_2_T*_x_* were drop cast onto plasma‐treated alumina membranes. A thin coating (∼4 nm) of Pt/Pd (80/20) was sputtered to assist imaging since alumina membranes were not conductive.

High‐resolution SEM images of Ti_3_C_2_T*_x_* flakes were acquired using an Apreo 2S Lo Vac SEM (ThermoFisher) with the accelerating voltage and working distance set to 10 kV and 10 mm, respectively. Fiji (ImageJ) software was used for measuring flake sizes, and 60 flakes were measured for each Ti_3_C_2_T*_x_* dispersion of different flake size.

#### 4.2.2 Raman Spectroscopy

Sample preparation for Raman spectroscopy was identical to that for SEM imaging. Raman spectra of freeze‐dried MXgel samples were acquired using an inVia Raman Microspectrometer (Renishaw plc) fitted with a 785 nm excitation diode laser operating at 10% power (10.5 mW). Each Raman spectrum was recorded with an acquisition time of 10 s.

#### 4.2.3 Zeta Potential Measurements

Zeta potential of Ti_3_C_2_T*_x_* dispersions, with and without Ca^2+^ ions, was measured using a Zeta Sizer equipped with an MP2 Auto titrator (Zetasizer Nano Zs, Malvern Panalytical). For all measurements, the concentration of Ti_3_C_2_T*_x_* aqueous dispersions used was ∼0.01 mg mL^−1^. The zeta potential of Ti_3_C_2_T*_x_* with Ca^2+^ ions (0.001 mg ml^−1^) was obtained by mixing the two components in the mass ratio of 10:1 (Ti_3_C_2_T*_x_*:Ca^2+^ ions).

#### 4.2.4 Viscoelasticity Measurements

Viscoelasticity measurements were performed using a Discovery HR‐3 Hybrid Rheometer (TA Instruments). MXgels were prepared directly on the instrument's sample loading plate by mixing 500 µL of 40 mg mL^−1^ Ti_3_C_2_T*_x_* with 500 µL of 100 mM CaCl_2_, matching the mass ratio of 10:1. Storage modulus (*G*') and loss modulus (*G*'') were recorded as a function of oscillation amplitude and frequency through oscillatory shear tests performed at 25°C. For amplitude sweeps, the frequency was set to 10 rad s^−1^ and strain was varied from 0.01%–10%. For frequency sweeps, the amplitude was set to 1%, and the frequency was varied from 0.1–100 rad s^−1^.

To calculate the shear yield stress of hydrated MXgel, the measured elastic stress was plotted as a function of oscillatory strain, and the peak elastic stress was determined [89, 90].

#### 4.2.5 Nanoindentation Measurements

Nanoindentation measurements to quantify the mechanical properties of MXgel were performed using an atomic force microscope (AFM, Asylum) fitted with a colloidal AFM tip with a radius of ∼2500 nm. MXgel samples with dimensions of 10 × 10 × 0.5 mm were loaded onto the AFM stage and hydrated with DI‐H_2_O. The force exerted by the AFM tip on the MXgel surface was subsequently measured as a function of indentation depth.

The Young's modulus of MXgel was calculated by fitting the recorded data with the Hertz model [91], defined by the following equation:

$$F=\frac{4}{3}\;*\frac{E}{1-\nu^{2}}*\sqrt{R}*\delta^{\frac{3}{2}}$$

where, *F*, *E*, *ʋ*, *R*, and *δ* are the force measured by the AFM tip, Young's modulus, Poisson's ratio (assumed 0.5 for hydrogel), radius of the AFM tip (∼2500 nm), and indentation depth, respectively.

#### 4.2.6 Electrical Conductivity Measurements

Hydrogel samples were prepared into molds with dimensions of either 2 × 15 × 0.50 mm or 2 × 15 × 0.25 mm. The crosslinked hydrogels were gently washed three times with DI‐H_2_O to remove excess ions. Electrical resistance was measured using a four‐point probe apparatus (Loresta‐AX, MCP‐T370) with a probe spacing of ∼1.6 mm. Electrical conductivity (*σ*) was calculated following the equation:

$$\sigma =l/\left(R*A\right)$$

where *l*, *R*, and *A* are the inter‐probe spacing (1.6 mm), measured resistance, and cross‐sectional area of the sample, respectively.

#### 4.2.7 Inductively Coupled Plasma Optical Emission Spectroscopy (ICP‐OES) for Ca^2+^

Elemental analysis of Ca^2+^ was performed using an Agilent 5900 ICP‐OES instrument. Calibration curves were constructed using Ca^2+^ standard solutions with concentrations of 0, 5, 8.33, 12.50, and 25 ppm in DI‐H_2_O prepared by serial dilution of certified standards (Agilent). MXgels were soaked in 5 mL of DI‐H_2_O and aCSF at 37°C for 5 min and 24 h, and the sample solutions were collected. Individual sample solutions were then independently filtered using a 0.22 µm PTFE syringe filters. Measurements were conducted under an RF power of 1.2 kW, with plasma, auxiliary, and nebulizer gas flow rates of 12, 1, and 0.7 L min^−^^1^, respectively. Ca^2+^ was quantified at a wavelength of 396.847 nm, with Y (371.029 nm) used as an internal standard. Calibration curves exhibited excellent linearity (*R* ^2^ > 0.99), with a relative standard deviation (RSD) below 0.3% based on three independent measurements per sample.

#### 4.2.8 Tensile Testing of Gelapin

The mechanical properties of gelapin samples were measured using a Discovery HR‐3 Hybrid Rheometer (TA Instruments). Briefly, gelapin samples with varying degrees of crosslinking were prepared as dog‐bone structures with dimensions 10 × 5 × 0.5 mm. The samples were gently clamped into position, and tensile forces were applied at a constant strain rate until failure. The Young's modulus was determined from the slope of the linear region of the stress‐strain curves.

#### 4.2.9 Gelapin Degradation

For the gelapin degradation assay, 10 × 10 × 1 mm coupons of gelapin with varying degrees of crosslinking were fabricated. The weight of each sample was measured post‐gelation. The samples were then placed in 1X PBS at 37°C. The samples were gently taken out of the 1X PBS at period intervals and excess liquid was soaked using Kimwipes (Kimtech Science). The samples were then weighed and reintroduced into the 1X PBS at 37°C. Once the samples for a particular crosslinking condition failed to hold their physical structure, they were considered to be degraded.

#### 4.2.10 MXgel Degradation

To characterize the structural degradation of MXgel, Ti_3_C_2_T*_x_* MXgel samples were prepared in 2 mL centrifuge tubes. Post gelation, 1 mL of 1X PBS or 1 mL of 1X PBS with 30 mM H_2_O_2_ (JT Baker, 2190‐03) was added to the samples, and the samples were then placed in an oven at 37°C. After a fixed amount of time (22 days for samples in 1X PBS and 4 days for samples in 1X PBS with 30 mM H_2_O_2_), the samples were washed three times with DI‐H_2_O and SEM/EDS characterization was performed. Four MXgel samples were used for each condition. 1X PBS solution was refreshed weekly, while the 1X PBS with 30 mM H_2_O_2_ solution was refreshed daily. To characterize the demonstrated tunability of functional properties of MXgel, Ti_3_C_2_T*_x_* colloids with different flake sizes were fabricated. Subsequently, 2 × 15 × 0.50 mm MXgel samples were fabricated. The MXgel samples were placed in 1X PBS with 30 mM H_2_O_2_, and electrical conductivity was measured using the four‐point probe technique at regular time intervals. The electrical conductivity data were fit with equation:

$$\sigma =\sigma_{0}\;*\exp\left(-t/\tau \right)$$

where *σ*, *σ*_0_, *t*, and *τ* are the electrical conductivity, electrical conductivity at *t* = 0 h, time (h), and the time constant (h), respectively.

#### 4.2.11 MXgel Swelling Behavior

To characterize the swelling behavior of MXgel, 10 × 10 × 0.5 mm Ti_3_C_2_T*_x_* MXgel samples were fabricated. The samples were gently rinsed 3*x* with DI‐H_2_O and equilibrated in 1X PBS for 10 min. After the equilibration period, the samples were weighed and subsequently soaked in 1X PBS at 37°C. The samples were gently taken out of the 1X PBS at period intervals and excess liquid was soaked using Kimwipes (Kimtech Science). The samples were then weighed and reintroduced into the 1X PBS at 37°C.

#### 4.2.12 Metabolic Activity and Viability Assays

MXgel and gelapin hydrogels were fabricated in 35‐mm Petri dishes and sterilized by UV exposure for 1 h. Hydrogel‐conditioned media extracts were generated by incubating hydrogel samples with 2.5 mL of astrocyte culture medium, which included neurobasal medium with 2% B‐27, 1% G‐5, 0.25% L‐glutamine, and 1% penicillin‐streptomycin, at 37°C and 5% CO_2_ for 24 h. The blank extract consisted of the same medium incubated under identical conditions in the absence of hydrogels. After 24 h, extracts were collected, sterile‐filtered through 0.22 µm filters, and applied to rat astrocytes seeded at 60,000 cells/mL in 96‐well plates for alamarBlue metabolic activity assays or 24‐well plates for live/dead assays. Rat astrocytes between passages 4–8 were used to achieve an enriched astrocytic population, as previously described [19]. Cells were exposed to the generated hydrogel‐conditioned media extracts for 24 h before endpoint analysis.

For metabolic activity analysis, cells were incubated with 10% v/v alamarBlue Cell Viability Reagent (Thermo Fisher Scientific), and fluorescence was measured at 1, 2, 3, and 4 h post‐reagent addition using a microplate reader (Ex 560/Em 590; top read). Matched no‐cell wells containing the same extracts and alamarBlue were included to control for extract‐dependent background and potential optical or chemical interference, and sample‐matched background subtraction was applied before analysis. A detergent‐treated positive control (0.05% Triton X‐100) was included to confirm assay sensitivity. Linear regression was performed on the 1–4 h time‐course data to verify that measurements were acquired within the linear dynamic range of the assay.

For live/dead analysis, astrocytes were incubated with 2 µM Calcein AM, 4 µM EthD‐1, and 2 µM Hoechst 33342 at room temperature for 30 min. Fluorescence images from randomly selected regions (*N* = 4 per condition) were acquired using a Keyence BZ‐X810 microscope with a 10*x* objective. Cellular viability was calculated following the equation:

$$Viability\;\;\left(\%\right)=\frac{Total\;No.\;of\;cells-No.\;of\;dead\;cells}{Total\;No.\;of\;cells}\;*100$$

### 4.3 MXgel Device Fabrication

To fabricate MXgel devices, a computer‐aided design (CAD) of the desired device geometry and electrode size was generated. This was used to 3D print a master mold using an ultra‐high resolution resin printer (Boston Micro Fabrication, S240). The master mold was used to create a negative mold of the desired print using a silicone elastomer (EcoFlex, 00–30). 100% crosslinked gelapin was cast into the elastomer negative mold and allowed to gel overnight. Once gelled, the gelapin substrate was carefully removed from the mold and placed onto a clean glass slide. The top side of the gelapin substrate was then coated with a thin film of 50 mg mL^−1^ PLGA_75:25_ (lactide:glycolide, 75:25) (Sigma‐Aldrich, P1941). Backend connectors were gently placed into the electrode channels while the PLGA was still wet. The PLGA film was allowed to dry overnight under vacuum in a desiccator at room temperature. Ti_3_C_2_T*_x_* dispersion (40 mg mL^−1^) was then carefully injected into the electrode traces using a syringe, followed by CaCl_2_ solution (100 mM). The electrodes were allowed to gel for 5 min before applying a top passivation of PLGA_75:25_. The top passivation layer was set for 15 min under vacuum in a desiccator at room temperature.

To fabricate MXgel bioelectronic devices with varying PLGA thickness and compositions, 100 mg mL^−1^ PLGA_75:25_ and 50 mg mL^−1^ PLGA_85:15_ (lactide:glycolide, 85:15) (Sigma‐Aldrich, 739979) were used.

### 4.4 Device Characterization

#### 4.4.1 Micro‐CT Characterization

Sample preparation for micro‐CT characterization involved freeze‐drying of fully fabricated MXgel bioelectronic devices. A freeze‐dried sample was glued on a pin and mounted on a pin sample holder to minimize any artifacts. Micro‐CT scans were acquired using the Xradia 620 Versa micro‐CT system (Zeiss), with two scans performed at different resolutions. In the first scan of the electrodes, the sample was scanned at 40 kV and 3 W using a 0.4*x* detector to achieve a resolution of 6.64 µm. The source‐to‐sample distance was set to 20.7 mm, and the detector‐to‐sample distance to 85.5 mm. The scan used a 5.5‐s exposure time, with 2401 projections taken over a 60‐degree sample rotation. No filter was applied to the x‐ray source during this scan. For the high‐resolution second scan, a 20*x* objective was used to achieve a resolution of 410 nm. The source‐to‐sample and detector‐to‐sample distances were 19.4 and 12.9 mm, respectively. This scan was performed at 70 kV and 6.5 W, with a 7‐s exposure time and no filter applied. The raw projections were processed using Zeiss XRM reconstructor software (v.2022.1), where standard corrections for center shift and beam hardening were applied. Further image refinement on the high‐resolution scan included Gaussian filtration to improve segmentation quality. For post‐processing, Dragonfly Pro software was used, applying thresholding with manual adjustments.

#### 4.4.2 Mechanical Characterization

The MXgel bioelectronic structures were fabricated as described above and hydrated in 1X PBS for 15 min. The samples were then loaded onto Instron 5564 (Instron, Illinois Tool Works). For tensile tests, the samples were loaded vertically between the tensile grips, and tensile forces were applied under a constant strain rate until failure. The Young's modulus of the samples was calculated from the linear region of the stress‐strain curves.

For bending tests, the samples were loaded onto a three‐point bending fixture, and constant force was applied until failure. The bending modulus (*E_f_*) was calculated using the following equation:

$$E_{f}=\frac{L^{3}}{4*b*h^{3}}\;*m$$

where, *L*, *b*, *h*, and *m* are the distance between the support spans, width of the sample, thickness of the sample, and slope of the linear portion of the force‐deflection plot, respectively.

#### 4.4.3 Cyclic Bending Test

For the cyclic bending test, MXgel traces were fabricated on gelapin substrates as described above. The devices were loaded onto Instron 5948 (Instron, Illinois Tool Works) and subjected to repeated bending via 20% compressive strain at a rate of 20% s^−1^. Two‐point probe electrical resistance measurements were performed using a multimeter.

#### 4.4.4 Electrochemical Characterization

Electrochemical characterizations were performed using a Gamry Reference 600 potentiostat (Gamry Instruments) with a three‐electrode cell composed of MXgel electrodes, graphite rode (Bio‐Rad Laboratories Inc.), and an Ag/AgCl electrode (CH Instruments, CHI111) as the working electrodes, counter electrode, as the reference electrode, respectively. We used 1X phosphate‐buffered saline (1X PBS, pH = 7.4) as the electrolyte. Electrode impedance spectroscopy (EIS) was performed under a 10 mV *V*_RMS_ sinusoidal potential (*V*_DC_ = 0 V) with decreasing AC frequencies from 10^5^–1 Hz. Cyclic voltammetry measurements were performed with a potential range set to −1.3 to +0.5 V and scan rates of 50, 100, 250, 500, and 1000 mV s^−1^. The cathodic charge storage capacity (CSC_C_) was calculated by computing the time integral of the cathodic current in the cyclic voltammograms. Voltage transients were measured by applying asymmetric biphasic current pulses with 500 µs cathodic, 200 µs inter‐pulse, and 2000 µs anodic phases. To maintain charge neutrality, the amplitude of the anodic current was set to 0.25x the amplitude of the cathodic current. The charge injection capacity (CIC) of the working electrode was computed as the area‐normalized charge injected at a maximum cathodic excursion of −1.3 V vs. Ag/AgCl. For crosstalk characterization, voltage transients were measured at every electrode of a device when a current pulse (0.5 mA, a 500‐ms cathodic pulse) was applied at a set working electrode. The difference in the cathodic potentials just before the end of the cathodic pulse was calculated to determine crosstalk.

For electrochemical fatigue testing, the MXgel electrodes were subjected to continuous application of biphasic current pulses with a cathodic phase of 1 mA–500 µs. The electrochemical impedance of the electrodes was measured after every 2,000 current pulses.

#### 4.4.5 MXgel Bioelectronics Degradation

To assess the structural degradation of MXgel bioelectronics, MXgel devices with 1 mm electrodes without any backend connectors were placed in 1X PBS at 37°C. Every day, the devices were gently taken out of their respective baths, gently dried using Kimwipes (Kimtech Science), and imaged with a stereoscope (AM Scope). To assess the functional degradation of MXgel bioelectronics, MXgel devices with 1 mm electrodes and backend connectors were stored in 1X PBS at 37°C such that only the electrodes were exposed to 1X PBS, and the backend connectors were outside the 1X PBS. Electrochemical impedance spectroscopy, cyclic voltammetry at 250 mV s^−1^, and voltage transient measurements were performed daily for all electrodes. This was followed by replacing the 1X PBS. Functional degradation was assessed for five devices with six electrodes per device. For both structural and functional degradation, 1X PBS was replaced daily with fresh 1X PBS.

### 4.5 In Vivo Recording and Stimulation

All animal procedures were approved by the Institutional Animal Care and Use Committees at the University of Pennsylvania (protocol #806241 and #806073) and the Corporal Michael Crescenz Veterans Affairs Medical Center (protocol #02003). Animal husbandry and experimental procedures were performed in accordance with the Guide for the Care and Use of Laboratory Animals and complied with the ARRIVE (Animal Research: Reporting of In Vivo Experiments) guidelines.

#### 4.5.1 Device Sterilization

To sterilize MXgel devices, fully‐fabricated devices were packaged in commercial ethylene oxide sterilization bags. The samples were sterilized in an ethylene oxide sterilizer (Anprolene, AN 74) using a single ethylene oxide ampule for the 12 h cycle.

#### 4.5.2 Recording and Stimulating Cortical Activity

Two adult female Long‐Evans rats (Crl:LE, 280–315 g) were used for in vivo CNS neuro‐electrophysiological testing. Anesthesia was induced with 5% isoflurane in O_2_ prior to placing the animal in a stereotaxic frame and administering systemic and local (scalp) analgesics. The respiratory rate and pedal reflex were used to monitor the depth of anesthesia. Subsequently, 1.0%–1.5% isoflurane was maintained during the experiments. The heart rate was measured using a custom pulse oximeter placed. For the cortical recording experiment, a craniotomy exposed the right whisker sensory cortex (+3 to +6 mm from midline, ‐5 to 0 mm from bregma) with the dura left intact. Two skull screws were anchored into the occipital and frontal bones as the ground and reference electrodes, respectively. A 1 mm MXgel electrode array was held against the exposed dura with a stereotaxic micromanipulator. The array was connected to an Intan RHS system (Intan Technologies) and neural activity was recorded at 30 kHz with a passband of 1.2 Hz to 7603.8 Hz. After recording spontaneous activity during varied levels of anesthesia, sensory‐evoked neural activity was recorded in response to mechanical stimulation of a contralateral whisker. The whisker, trimmed to 15 mm, was placed in a 0.2 mm capillary tube attached to a piezoelectric bender calibrated to deliver a 0.4 mm peak deflection. The stimulus waveform consisted of a 4‐ms ramp up, a 4‐ms plateau, and a 4‐ms ramp down, which was low‐pass filtered to avoid ringing. Mechanical stimuli were repeatedly delivered at 0.5 Hz, and the trigger for each mechanical stimulus was recorded.

For the cortical stimulation experiment, the right motor cortex was surgically exposed (+1 to +4 mm from midline, ‐3 to +3 mm from bregma) with the dura left intact. A skull screw serving as ground and stimulation return was placed over the cerebellum. A reference screw was placed in the left frontal bone. With a 0.5 mm MXgel electrode array held against the dura, electrical stimuli were delivered to chosen electrodes using the Intan RHS system while recording on other channels. The monopolar stimulation consisted of a 0.2‐ms/phase biphasic charge‐balanced pulses of variable amplitude delivered in trains of 100 pulses with a 3‐ms inter‐pulse interval. Trains were repeated every 10 s while the experimenters monitored contralateral evoked movement. Video recordings were performed using a camera. Intraperitoneal injection of sodium pentobarbital was used to euthanize the rats at the conclusion of the experiment.

#### 4.5.3 Analysis of Neural Recordings

The recorded neural data were bandpass filtered using a 4th‐order Butterworth filter for 4–150 Hz. Burst suppression ratio (*BSR*) was calculated following a previously described scheme [92]. Briefly, the first derivative of the recorded neural data was determined. Suppression events were then identified as instances when the first derivative of the recorded neural signals was within ± 0.15 µV s^−1^ and had a minimum duration of 200‐ms. A moving average filter with a 60 s window was then applied to calculate the *BSR* as a function of time.

The *SNR* was calculated using the following equation:

$$SNR\;\;\left(dB\right)=20*\log\left(\frac{Signal_{RMS}}{Noise_{RMS}}\right)$$

For sensory evoked neural data analysis, the RMS of the filtered neural signals was calculated over a 2‐ms moving window with 2‐ms displacement (no overlap). The recorded triggers for each mechanical stimulus were then used to accurately mark corresponding temporal instances in the RMS neural data. All stimulation pulses for whiskers A and B were binned into 600‐ms epochs with individual stimulation pulses at 100‐ms intervals. Average *RMS* neural data for all mechanical stimulation pulses for each electrode were then computed.

#### 4.5.4 Cortical Stimulation Analysis

The deflection of the jaw due to cortical stimulation was measured using Kinovea, a motion analysis software. Video recordings corresponding to each stimulation condition were loaded into Kinovea, and a coordinate system was established using the markings on the stereotactic assembly. To determine the deflection of the jaw due to the stimulation pulses, a whisker was selected and annotated across all videos. Automatic tracking of the annotated whisker was performed by Kinovea, and the maximum deflection for each stimulation pulse was recorded.

#### 4.5.5 Recording and Stimulation in the Peripheral Nervous System

Adult male Lewis rats (LEW/Crl; 300–330 g; 8–10 weeks old) used for the in vivo PNS neuro‐electrophysiological studies were housed in pairs under a 12 h light/dark cycle with unrestricted access to food and water. For acute studies, anesthesia was induced and maintained with isoflurane. The hindlimb was shaved and aseptically prepared using betadine solution. Preoperative analgesia was induced through subcutaneously administration of Meloxicam (2 mg kg^−1^) at the nape of the neck. Bupivacaine (2 mg kg^−1^) was also subcutaneously injected along the planned incision site for local analgesia. A skin incision was then made over the lateral aspect of the lower leg directly above the *tibialis anterior* muscle, with care taken to preserve the underlying fascia. The skin was gently dissected from the fascia to create a pocket for placement of the MXgel recording electrodes atop the muscle. The incision was closed using 6–0 nylon sutures. To record compound muscle action potentials (CMAPs), the gluteal musculature was bluntly dissected exposing the *sciatic nerve* at the sciatic notch. A monopolar subdermal needle electrode was positioned within the belly of the *tibialis anterior* muscle for recording, while a reference electrode was inserted into the distal tendon. Biphasic stimulation pulses (0.47–1.07 mA, 0.2‐ms pulse duration, 1 Hz) were applied to the exposed *sciatic nerve* using bipolar MXgel stimulation electrodes. Following determination of the activation threshold, the stimulation intensity was progressively increased until the CMAP amplitude reached a plateau. Responses were recorded using a Natus Viking EDX system (gain: 100X; bandpass filter: 10–10,000 Hz; notch filter: 60 Hz). Following the procedure, the incision was closed with staples, and animals were monitored during recovery before being returned to the vivarium. The procedure for implanting MXgel recording‐, control‐, and reference‐ electrodes for the multi‐day implantation experiments was similar to that described above; however, no stimulation electrodes were implanted. Under anesthesia, a handheld bipolar stimulator was percutaneously placed near the *sciatic nerve*, and stimulating pulses were delivered (0.10–5.00 mA, 0.2‐ms pulse duration, 1 Hz). After identifying the activation threshold, the stimulation intensity was again gradually increased until the CMAP amplitude plateaued. Responses were recorded using a Natus Viking EDX system (gain: 100X; bandpass filter: 10–10,000 Hz; notch filter: 60 Hz). Following this procedure, the incision was closed with sutures, and animals were monitored during recovery before being returned to the vivarium.

### 4.6 In Vivo Biocompatibility Analysis

The in vivo biocompatibility of MXgel bioelectronics on the underlying tissues was evaluated by implanting MXgel devices without the backend connectors atop the *biceps femoris* muscle. Here, a 5 mm skin incision was made, and the fascia was bluntly dissected to accommodate the MXgel bioelectronics. Once the MXgel bioelectronics were placed over the ipsilateral *biceps femoris*, the skin was closed with 6–0 nylon sutures.

#### 4.6.1 Ultrasound Imaging of Implanted MXgel Bioelectronics

For non‐invasive imaging of the implanted MXgel bioelectronics, ultrasound imaging was performed on a weekly basis. Prior to imaging, the animals were anesthetized via isoflurane inhalation and the hindlimb was shaved. A layer of acoustic coupling gel (Aquasonic) was applied over the skin at the implant site, and ultrasound images were acquired using a Vevo 2100 (FUJIFILM VisualSonics Inc.) ultrasound imaging system fitted with an MS550D transducer (22–55 MHz). Both longitudinal and transverse cross‐sectional images were recorded for each animal every week up to their respective terminal time points (*N* = 3 images per orientation). The dimensions of the implanted devices were measured using Fiji (ImageJ). The overall thickness of the devices at each time point was determined by calculating the average thickness from both the longitudinal and transverse sections.

#### 4.6.2 Ti^4+^ Bioaccumulation Analysis

Post‐euthanasia, at the terminal time points of 4 and 8 weeks post‐implantation, the liver, kidneys, and spleen were excised and immediately flash‐frozen in liquid N_2_. Subsequently, tissue samples (500 mg of liver, 500 mg of kidney, and 200 mg of spleen) were weighed and transferred into glass vials. The tissues were chemically digested using 1 mL of HNO_3_ (70%) and 0.25 mL of H_2_O_2_ (30%) at 65°C for 2.5 h. The digestion process was subsequently completed overnight at room temperature. Once fully digested, the resulting solutions were diluted with DI‐H_2_O to volumes of 10 mL. Any residual particulates were removed by individually filtering the sample solutions through 0.22 µm PTFE syringe filters. Finally, elemental analysis for the quantification of Ti^4+^ concentrations was performed using an Agilent 5900 ICP‐OES.

#### 4.6.3 Histological Analysis

The whole *biceps femoris* muscle was harvested post‐euthanasia and submerged in 4% paraformaldehyde (PFA, Electron Microscopy Sciences) for 24 h. The tissue samples were then embedded in paraffin, sectioned, mounted onto glass slides, and stained using hematoxylin and eosin (H&E). Stained sections were imaged using an Aperio Versa 200 slide scanner (Leica BioSystems) and the Aperio ImageScope software (Leica BioSystems). Histological analyses including tissues sectioning, H&E staining, and slide evaluations were performed by a trained veterinary pathologist at the Penn Vet Comparative Pathology Core.

### 4.7 Statistical Analysis

All data processing and analysis were performed using MATLAB R2024b (The MathWorks, Inc.). Statistical comparisons were conducted using one‐way analysis of variance (ANOVA), followed by Tukey's *post‐hoc* multiple‐comparison test.

## Author Contributions

**Raghav Garg**: conceptualization, investigation, writing – original draft, methodology, validation, visualization, writing – review and editing, formal analysis. **Franco A. Laimo**: investigation, methodology, validation, writing – review and editing, formal analysis. **Stefano Ippolito**: formal analysis, investigation, methodology, validation, writing – review and editing. **Prastuti Upadhyay**: investigation, methodology, validation, writing – review and editing, formal analysis. **Spencer R. Averbeck**: investigation, methodology, validation, writing – review and editing, formal analysis, visualization. **Dimitris Boufidis**: investigation, methodology, validation, visualization, writing – review and editing, formal analysis. **Charles‐Antoine Assenmacher**: investigation, methodology, validation, visualization, writing – review and editing, formal analysis. **Dayo O. Adewole**: investigation, methodology, validation, writing – review and editing. **Sai Panigrahy**: investigation, methodology, validation, writing – review and editing, formal analysis. **Bita Soltan Mohammadlou**: investigation, methodology, validation, visualization, writing – review and editing, formal analysis. **Lucy Plant**: investigation, methodology, validation, writing – review and editing, formal analysis. **Yuan Zhang**: investigation, methodology, validation, writing – review and editing, formal analysis. **Andrew G. Richardson**: investigation, methodology, validation, writing – review and editing, visualization, formal analysis, resources, funding acquisition. **Yury Gogotsi**: investigation, methodology, writing – review and editing, supervision, resources, funding acquisition. **D. Kacy Cullen**: investigation, methodology, writing – review and editing, supervision, resources, funding acquisition. **Flavia Vitale**: conceptualization, investigation, funding acquisition, writing – original draft, writing – review and editing, methodology, supervision, resources.

## Conflicts of Interest

F.V. and Y.G. are co‐inventors on US Patent “Implantable devices using 2D metal carbides and nitrides (MXenes)” (Patent No: 11,925,466 B2). Y.G. is a co‐founder of MXene, Inc., a company that manufactures MXene materials.

## Supporting information




**Supporting File**: adma75137‐sup‐0001‐SuppMat.pdf.

## Data Availability

The data that support the findings of this study are available from the corresponding author upon reasonable request.

## References

[1] S. P. Lacour , G. Courtine , and J. Guck , “Materials and Technologies for Soft Implantable Neuroprostheses,” Nature Reviews Materials 1 (2016): 16063.

[2] R. Feiner and T. Dvir , “Tissue–Electronics Interfaces: from Implantable Devices to Engineered Tissues,” Nature Reviews Materials 3 (2017): 17076.

[3] D. Boufidis , R. Garg , E. Angelopoulos , D. K. Cullen , and F. Vitale , “Bio‐Inspired Electronics: Soft, Biohybrid, and “Living” Neural Interfaces,” Nature Communications 16 (2025): 1861. 10.1038/s41467-025-57016-0 PMC11845577 39984447

[4] H. Yuk , B. Lu , and X. Zhao , “Hydrogel Bioelectronics,” Chemical Society Reviews 48 (2019): 1642–1667. 10.1039/c8cs00595h 30474663

[5] R. Akhtar , M. J. Sherratt , J. K. Cruickshank , and B. Derby , “Characterizing the Elastic Properties of Tissues,” Materials Today 14 (2011): 96–105. 10.1016/S1369-7021(11)70059-1 PMC3378034 22723736

[6] V. S. Polikov , P. A. Tresco , and W. M. Reichert , “Response of Brain Tissue to Chronically Implanted Neural Electrodes,” Journal of Neuroscience Methods 148 (2005): 1–18. 10.1016/j.jneumeth.2005.08.015 16198003

[7] J. W. Salatino , K. A. Ludwig , T. D. Y. Kozai , and E. K. Purcell , “Glial Responses to Implanted Electrodes in the Brain,” Nature Biomedical Engineering 1 (2017): 862–877. 10.1038/s41551-017-0154-1 PMC6261524 30505625

[8] R. Chen , A. Canales , and P. Anikeeva , “Neural Recording and Modulation Technologies,” Nature Reviews Materials 2 (2017): 16093. 10.1038/natrevmats.2016.93 PMC6707077 31448131

[9] Y. S. Zhang and A. Khademhosseini , “Advances in Engineering Hydrogels,” Science 356 (2017): aaf3627. 10.1126/science.aaf3627 PMC5841082 28473537

[10] Y. Zhang , Y. Tan , J. Lao , H. Gao , and J. Yu , “Hydrogels for Flexible Electronics,” ACS Nano 17 (2023): 9681–9693. 10.1021/acsnano.3c02897 37199537

[11] Y. Choi , J. Koo , and J. A. Rogers , “Inorganic Materials for Transient Electronics in Biomedical Applications,” MRS Bulletin 45 (2020): 103–112.

[12] H.‐S. Han , S. Loffredo , I. Jun , et al., “Current Status and Outlook on the Clinical Translation of Biodegradable Metals,” Materials Today 23 (2019): 57–71.

[13] C. Li , C. Guo , V. Fitzpatrick , et al., “Design of Biodegradable, Implantable Devices towards Clinical Translation,” Nature Reviews Materials 5 (2020): 61–81.

[14] G. D. Cha , D. Kang , J. Lee , and D. H. Kim , “Bioresorbable Electronic Implants: History, Materials, Fabrication, Devices, and Clinical Applications,” Advanced Healthcare Materials 8 (2019): 1801660. 10.1002/adhm.201801660 30957984

[15] A. H. M. Sanchez , B. J. Luthringer , F. Feyerabend , and R. Willumeit , “Mg and Mg Alloys: How Comparable Are in Vitro and in Vivo Corrosion Rates? A Review,” Acta Biomaterialia 13 (2015): 16–31. 10.1016/j.actbio.2014.11.048 25484334

[16] K. J. Yu , D. Kuzum , S.‐W. Hwang , et al., “Bioresorbable Silicon Electronics for Transient Spatiotemporal Mapping of Electrical Activity from the Cerebral Cortex,” Nature Materials 15 (2016): 782–791. 10.1038/nmat4624 PMC4919903 27088236

[17] C. Yang , B. Wang , J. Wan , A. Kamath , F. You , and B. Tian , “Quantifying the Global Eco‐Footprint of Wearable Healthcare Electronics,” Nature 649 (2026): 73–82. 10.1038/s41586-025-09819-w 41476272

[18] R. Garg and F. Vitale , “Latest Advances on MXenes in Biomedical Research and Health Care,” MRS Bulletin 48 (2023): 283–290. 10.1557/s43577-023-00480-0 PMC9943034 36846314

[19] D. Boufidis , E. N. Krizman , C. M. Smith , et al., “Astrocyte Interactions with Ti_3_C_2_T* _x_ * MXene Flakes: Insights into Viability, Morphology, and Functionality,” Advanced Materials Interfaces 12 (2025): 00261. 10.1002/admi.202500261 PMC12747572 41472803

[20] A. M. Jastrzebska , A. Szuplewska , T. Wojciechowski , et al., “In Vitro Studies on Cytotoxicity of Delaminated Ti_3_C_2_ MXene,” Journal of Hazardous Materials 339 (2017): 1–8. 10.1016/j.jhazmat.2017.06.004 28601597

[21] Y. Ohm , C. Pan , M. J. Ford , X. Huang , J. Liao , and C. Majidi , “An Electrically Conductive Silver–Polyacrylamide–Alginate Hydrogel Composite for Soft Electronics,” Nature Electronics 4 (2021): 185–192.

[22] C. M. Tringides , N. Vachicouras , I. de Lázaro , et al., “Viscoelastic Surface Electrode Arrays to Interface with Viscoelastic Tissues,” Nature Nanotechnology 16 (2021): 1019–1029. 10.1038/s41565-021-00926-z PMC9233755 34140673

[23] J. Deng , H. Yuk , J. Wu , et al., “Electrical Bioadhesive Interface for Bioelectronics,” Nature Materials 20 (2021): 229–236. 10.1038/s41563-020-00814-2 32989277

[24] H. Montazerian , E. Davoodi , C. Wang , et al., “Boosting Hydrogel Conductivity via Water‐Dispersible Conducting Polymers for Injectable Bioelectronics,” Nature Communications 16 (2025): 3755. 10.1038/s41467-025-59045-1 PMC12015517 40263282

[25] J. Chong , C. Sung , K. S. Nam , et al., “Highly Conductive Tissue‐Like Hydrogel Interface through Template‐Directed Assembly,” Nature Communications 14 (2023): 2206. 10.1038/s41467-023-37948-1 PMC10113367 37072411

[26] S. Huang , G. Xiang , and V. N. Mochalin , “Formation of Hydrocarbons and Carbon Oxides in MXene Reactions with Water under Varying Oxidative Conditions,” Nanoscale 17 (2025): 9937–9946. 10.1039/d4nr04937c 40029167

[27] K. P. Marquez , K. M. D. Sisican , R. P. Ibabao , et al., “Understanding the Chemical Degradation of Ti_3_C_2_T* _x_ * MXene Dispersions: A Chronological Analysis,” Small Science 4 (2024): 2400150. 10.1002/smsc.202400150 PMC11935063 40212246

[28] S. Huang and V. N. Mochalin , “Hydrolysis of 2D Transition‐Metal Carbides (MXenes) in Colloidal Solutions,” Inorganic Chemistry 58 (2019): 1958–1966. 10.1021/acs.inorgchem.8b02890 30649863

[29] A. VahidMohammadi , J. Rosen , and Y. Gogotsi , “The World of Two‐Dimensional Carbides and Nitrides (MXenes),” Science 372 (2021): abf1581. 10.1126/science.abf1581 34112665

[30] Y. Deng , T. Shang , Z. Wu , et al., “Fast Gelation of Ti_3_C_2_T* _x_ * MXene Initiated by Metal Ions,” Advanced Materials 31 (2019): 1902432. 10.1002/adma.201902432 31513318

[31] Z. Zhang , Z. Yao , Y. Li , S. Lu , X. Wu , and Z. Jiang , “Cation‐Induced Ti_3_C_2_T* _x_ * MXene Hydrogel for Capacitive Energy Storage,” Chemical Engineering Journal 433 (2022): 134488.

[32] G. Rayner‐Canham and T. Overton , Descriptive Inorganic Chemistry (W. H. Freeman, 2003).

[33] A. Sarycheva and Y. Gogotsi , “Raman Spectroscopy Analysis of the Structure and Surface Chemistry of Ti_3_C_2_T* _x_ * MXene,” Chemistry of Materials 32 (2020): 3480–3488.

[34] W. D. Fraser and D. N. Alter , “Bone and Mineral Metabolism,” in Tietz Textbook of Laboratory Medicine, 7th ed., ed. N. Rifai , R. W. K. Chiu , I. Young , C.‐A. D. Burnham , and C. T. Wittwer (Elsevier, 2023), 9780323775724.

[35] B. Akuzum , K. Maleski , B. Anasori , et al., “Rheological Characteristics of 2D Titanium Carbide (MXene) Dispersions: a Guide for Processing MXenes,” ACS Nano 12 (2018): 2685–2694. 10.1021/acsnano.7b08889 29463080

[36] V. R. Feig , H. Tran , M. Lee , and Z. Bao , “Mechanically Tunable Conductive Interpenetrating Network Hydrogels That Mimic the Elastic Moduli of Biological Tissue,” Nature Communications 9 (2018): 2740. 10.1038/s41467-018-05222-4 PMC6048132 30013027

[37] C. W. Macosko , Rheology: Principles, Measurements, and Applications (Wiley‐VCH, 1994).

[38] T. S. Mathis , K. Maleski , A. Goad , et al., “Modified MAX Phase Synthesis for Environmentally Stable and Highly Conductive Ti_3_C_2_ MXene,” ACS Nano 15 (2021): 6420–6429. 10.1021/acsnano.0c08357 33848136

[39] M. Brini , T. Calì , D. Ottolini , and E. Carafoli , “Neuronal Calcium Signaling: Function and Dysfunction,” Cellular and Molecular Life Sciences 71 (2014): 2787–2814. 10.1007/s00018-013-1550-7 PMC11113927 24442513

[40] J. L. Hart , K. Hantanasirisakul , A. C. Lang , et al., “Control of MXenes' Electronic Properties through Termination and Intercalation,” Nature Communications 10 (2019): 522. 10.1038/s41467-018-08169-8 PMC6355901 30705273

[41] H. Sun , S. Wang , P. Wang , et al., “Highly Stretchable, Adhesive, and Conductive PEDOT Nanocomposite Hydrogels for High‐Performance Flexible Bioelectronics,” Advanced Science 12 (2025): 13487. 10.1002/advs.202513487 PMC12752577 41057996

[42] T. Zhou , H. Yuk , F. Hu , et al., “3D Printable High‐Performance Conducting Polymer Hydrogel for all‐Hydrogel Bioelectronic Interfaces,” Nature Materials 22 (2023): 895–902. 10.1038/s41563-023-01569-2 37322141

[43] Y. Liu , J. Liu , S. Chen , et al., “Soft and Elastic Hydrogel‐Based Microelectronics for Localized Low‐Voltage Neuromodulation,” Nature Biomedical Engineering 3 (2019): 58–68. 10.1038/s41551-018-0335-6 30932073

[44] L. Shan , Y. Xue , X. Chen , et al., “Mechanically Compliant and Impedance Matching Hydrogel Bioelectronics for Low‐Voltage Peripheral Neuromodulation,” Advanced Materials 38 (2025): 11014. 10.1002/adma.202511014 40878426

[45] D. M. Kirchmajer , C. A. Watson , and M. Ranson , “Gelapin, a Degradable Genipin Cross‐Linked Gelatin Hydrogel,” RSC Advances 3 (2013): 1073–1081.

[46] S.‐M. Lien , W.‐T. Li , and T.‐J. Huang , “Genipin‐crosslinked Gelatin Scaffolds for Articular Cartilage Tissue Engineering with a Novel Crosslinking Method,” Materials Science and Engineering: C 28 (2008): 36–43.

[47] G. Balakrishnan , A. Bhat , D. Naik , et al., “Gelatin‐Based Ingestible Impedance Sensor to Evaluate Gastrointestinal Epithelial Barriers,” Advanced Materials 35 (2023): 2211581. 10.1002/adma.202211581 PMC10192083 36799712

[48] C. Gabriel , A. Peyman , and E. H. Grant , “Electrical Conductivity of Tissue at Frequencies below 1 MHz,” Physics in Medicine and Biology 54 (2009): 4863–4878. 10.1088/0031-9155/54/16/002 19636081

[49] International Organization for Standardization , Biological Evaluation of Medical Devices‐ Part 5: Tests for In Vitro Cytotoxicity (ISO 10993–5:2009), ISO, Switzerland (2009).

[50] A. Hoppe , N. S. Güldal , and A. R. Boccaccini , “A Review of the Biological Response to Ionic Dissolution Products from Bioactive Glasses and Glass‐Ceramics,” Biomaterials 32 (2011): 2757–2774. 10.1016/j.biomaterials.2011.01.004 21292319

[51] C. Delierneux , S. Kouba , S. Shanmughapriya , M. Potier‐Cartereau , M. Trebak , and N. Hempel , “Mitochondrial Calcium Regulation of Redox Signaling in Cancer,” Cells 9 (2020): 432. 10.3390/cells9020432 PMC7072435 32059571

[52] Y. Wang , R. Garg , J. E. Hartung , et al., “Ti_3_C_2_T* _x_ * MXene Flakes for Optical Control of Neuronal Electrical Activity,” ACS Nano 15 (2021): 14662–14671. 10.1021/acsnano.1c04431 PMC9285622 34431659

[53] Y. Wang , J. E. Hartung , A. Goad , et al., “Photothermal Excitation of Neurons Using MXene: Cellular Stress and Phototoxicity Evaluation,” Advanced Healthcare Materials 13 (2024): 2302330. 10.1002/adhm.202302330 PMC10963341 37755313

[54] Y.‐S. Chen , J.‐Y. Chang , C.‐Y. Cheng , F.‐J. Tsai , C.‐H. Yao , and B.‐S. Liu , “An in Vivo Evaluation of a Biodegradable Genipin‐Cross‐Linked Gelatin Peripheral Nerve Guide Conduit Material,” Biomaterials 26 (2005): 3911–3918. 10.1016/j.biomaterials.2004.09.060 15626438

[55] B. Amsden , “Solute Diffusion within Hydrogels. Mechanisms and Models,” Macromolecules 31 (1998): 8382–8395.

[56] C. Boehler , S. Carli , L. Fadiga , T. Stieglitz , and M. Asplund , “Tutorial: Guidelines for Standardized Performance Tests for Electrodes Intended for Neural Interfaces and Bioelectronics,” Nature Protocols 15 (2020): 3557–3578. 10.1038/s41596-020-0389-2 33077918

[57] S. F. Cogan , “Neural Stimulation and Recording Electrodes,” Annual Review of Biomedical Engineering 10 (2008): 275–309. 10.1146/annurev.bioeng.10.061807.160518 18429704

[58] W. Franks , I. Schenker , P. Schmutz , and A. Hierlemann , “Impedance Characterization and Modeling of Electrodes for Biomedical Applications,” IEEE Transactions on Biomedical Engineering 52 (2005): 1295–1302. 10.1109/TBME.2005.847523 16041993

[59] K. Shen , O. Chen , J. L. Edmunds , D. K. Piech , and M. M. Maharbiz , “Translational Opportunities and Challenges of Invasive Electrodes for Neural Interfaces,” Nature Biomedical Engineering 7 (2023): 424–442. 10.1038/s41551-023-01021-5 37081142

[60] V. Viswam , M. E. J. Obien , F. Franke , U. Frey , and A. Hierlemann , “Optimal Electrode Size for Multi‐Scale Extracellular‐Potential Recording from Neuronal Assemblies,” Frontiers in Neuroscience 13 (2019): 385. 10.3389/fnins.2019.00385 PMC6498989 31105515

[61] J. B. Johnson , “Thermal Agitation of Electricity in Conductors,” Physical Review 32 (1928): 97–109.

[62] M. R. Lukatskaya , S. Kota , Z. Lin , et al., “Ultra‐High‐Rate Pseudocapacitive Energy Storage in Two‐Dimensional Transition Metal Carbides,” Nature Energy 2 (2017): 17105.

[63] L. Bi , R. Garg , N. Noriega , et al., “Soft, Multifunctional MXene‐Coated Fiber Microelectrodes for Biointerfacing,” ACS Nano 18 (2024): 23217–23231. 10.1021/acsnano.4c05797 PMC11363215 39141004

[64] N. Driscoll , B. Erickson , B. B. Murphy , et al., “MXene‐Infused Bioelectronic Interfaces for Multiscale Electrophysiology and Stimulation,” Science Translational Medicine 13 (2021): abf8629. 10.1126/scitranslmed.abf8629 PMC8722432 34550728

[65] R. Garg , G. Balakrishnan , R. B. Rashid , et al., “Graphene and Poly(3,4‐ethylenedioxythiophene)–Polystyrene Sulfonate Hybrid Nanostructures for Input/Output Bioelectronics,” ACS Applied Nano Materials 6 (2023): 8495–8505.

[66] F. Posey and T. Morozumi , “Theory of Potentiostatic and Galvanostatic Charging of the Double Layer in Porous Electrodes,” Journal of the Electrochemical Society 113 (1966): 176.

[67] W. A. Rutala and D. J. Weber , HICPAC, “Guideline for Disinfection and Sterilization in Healthcare Facilities,” *U.S. Centers for Disease Control and Prevention* (2008). https://www.cdc.gov/infection‐control/hcp/disinfection‐and‐sterilization/index.html.

[68] S. R. Averbeck , D. Xu , B. B. Murphy , et al., “Stability of Ti_3_C_2_T* _x_ * MXene Films and Devices under Clinical Sterilization Processes,” ACS Nano 17 (2023): 9442–9454. 10.1021/acsnano.3c01525 PMC11342293 37171407

[69] J. M. Anderson , A. Rodriguez , and D. T. Chang , “Foreign Body Reaction to Biomaterials,” Seminars in Immunology 20 (2008): 86–100. 10.1016/j.smim.2007.11.004 PMC2327202 18162407

[70] J. M. Anderson and M. S. Shive , “Biodegradation and Biocompatibility of PLA and PLGA Microspheres,” Advanced Drug Delivery Reviews 28 (1997): 5–24. 10.1016/s0169-409x(97)00048-3 10837562

[71] S. Jafari , B. Mahyad , H. Hashemzadeh , S. Janfaza , T. Gholikhani , and L. Tayebi , “Biomedical Applications of TiO_2_ Nanostructures: Recent Advances,” International Journal of Nanomedicine 2020 (2020): 3447–3470. 10.2147/IJN.S249441 PMC7234979 32523343

[72] K. M. Buettner and A. M. Valentine , “Bioinorganic Chemistry of Titanium,” Chemical Reviews 112 (2012): 1863–1881. 10.1021/cr1002886 22074443

[73] E. S. Ereifej , G. M. Rial , J. K. Hermann , et al., “Implantation of Neural Probes in the Brain Elicits Oxidative Stress,” Frontiers in Bioengineering and Biotechnology 6 (2018): 9. 10.3389/fbioe.2018.00009 PMC5816578 29487848

[74] T. G. Park , “Degradation of Poly(Lactic‐co‐Glycolic Acid) Microspheres: Effect of Copolymer Composition,” Biomaterials 16 (1995): 1123–1130. 10.1016/0142-9612(95)93575-x 8562787

[75] F. Xia , J. Lao , R. Yu , et al., “Ambient Oxidation of Ti_3_C_2_ MXene Initialized by Atomic Defects,” Nanoscale 11 (2019): 23330–23337. 10.1039/c9nr07236e 31793604

[76] F. Cao , Y. Zhang , H. Wang , et al., “Recent Advances in Oxidation Stable Chemistry of 2D MXenes,” Advanced Materials 34 (2022): 2107554. 10.1002/adma.202107554 34816509

[77] K. Maleski , C. E. Ren , M.‐Q. Zhao , B. Anasori , and Y. Gogotsi , “Size‐Dependent Physical and Electrochemical Properties of Two‐Dimensional MXene Flakes,” ACS Applied Materials & Interfaces 10 (2018): 24491–24498. 10.1021/acsami.8b04662 29956920

[78] C. J. Zhang , S. Pinilla , N. McEvoy , et al., “Oxidation Stability of Colloidal Two‐Dimensional Titanium Carbides (MXenes),” Chemistry of Materials 29 (2017): 4848–4856.

[79] G. Hong and C. M. Lieber , “Novel Electrode Technologies for Neural Recordings,” Nature Reviews Neuroscience 20 (2019): 330–345. 10.1038/s41583-019-0140-6 PMC6531316 30833706

[80] A. Suarez‐Perez , G. Gabriel , B. Rebollo , et al., “Quantification of Signal‐to‐Noise Ratio in Cerebral Cortex Recordings Using Flexible MEAs with Co‐Localized Platinum Black, Carbon Nanotubes, and Gold Electrodes,” Frontiers in Neuroscience 12 (2018): 862. 10.3389/fnins.2018.00862 PMC6282047 30555290

[81] E. N. Brown , R. Lydic , and N. D. Schiff , “General Anesthesia, Sleep, and Coma,” New England Journal of Medicine 363 (2010): 2638–2650. 10.1056/NEJMra0808281 PMC3162622 21190458

[82] L. D. Lewis , S. Ching , V. S. Weiner , et al., “Local Cortical Dynamics of Burst Suppression in the Anaesthetized Brain,” Brain 136 (2013): 2727–2737. 10.1093/brain/awt174 PMC3754454 23887187

[83] C. Schmitz , J. E. Smith , I. Rachinskiy , et al., “Supracortical Microstimulation: Advances in Microelectrode Design and in Vivo Validation,” Annual Review of Biomedical Engineering 27 (2025): 235–254. 10.1146/annurev-bioeng-103023-072855 39914882

[84] K. A. Schafer , J. Eighmy , J. D. Fikes , et al., “Use of Severity Grades to Characterize Histopathologic Changes,” Toxicologic Pathology 46 (2018): 256–265. 10.1177/0192623318761348 29529947

[85] V. Nair , A. N. Dalrymple , Z. Yu , et al., “Miniature Battery‐Free Bioelectronics,” Science 382 (2023): abn4732. 10.1126/science.abn4732 37943926

[86] H.‐Y. Ahn , J. B. Walters , R. Avila , et al., “Bioresorbable, Wireless Dual Stimulator for Peripheral Nerve Regeneration,” Nature Communications 16 (2025): 4752. 10.1038/s41467-025-59835-7 PMC12098704 40404648

[87] A. Inman , B. S. Mohammadlou , K. Shevchuk , et al., “MXene‐Enabled Textile‐Based Energy Grid Utilizing Wireless Charging,” Materials Today 81 (2024): 59–69. 10.1016/j.mattod.2024.10.008 PMC13166144 42130647

[88] M. Alhabeb , K. Maleski , B. Anasori , et al., “Guidelines for Synthesis and Processing of Two‐Dimensional Titanium Carbide (Ti_3_C_2_T* _x_ * MXene),” Chemistry of Materials 29 (2017): 7633–7644.

[89] H. Walls , S. B. Caines , A. M. Sanchez , and S. A. Khan , “Yield Stress and Wall Slip Phenomena in Colloidal Silica Gels,” Journal of Rheology 47 (2003): 847–868.

[90] M. C. Yang , L. Scriven , and C. Macosko , “Some Rheological Measurements on Magnetic Iron Oxide Suspensions in Silicone Oil,” Journal of Rheology 30 (1986): 1015–1029.

[91] M. Krieg , G. Fläschner , D. Alsteens , et al., “Atomic Force Microscopy‐Based Mechanobiology,” Nature Reviews Physics 1 (2019): 41–57.

[92] P. Vijn and J. R. Sneyd , “I.V. Anaesthesia and EEG Burst Suppression in Rats: Bolus Injections and Closed‐Loop Infusions,” British Journal of Anaesthesia 81 (1998): 415–421. 10.1093/bja/81.3.415 9861133

