# Supplementary material for: Soft and Transient Bioelectronics Enabled by Polymer‐Free Ti3C2T x MXene Hydrogels
Source: Adv Mater. 2026 Sep 25;38(63):e75137. doi: 10.1002/adma.75137 (PMC13755660; doi:10.1002/adma.75137)
Supplement: Supplementary file 1 [file ADMA-38-e75137-s001.pdf]

Supporting Information

Soft and Transient Bioelectronics Enabled by Polymer-free $Ti_3C_2T_x$ MXene Hydrogels

*Raghav Garg, Franco A. Laimo, Stefano Ippolito, Prastuti Upadhyay, Spencer R. Averbeck, Dimitris Boufidis, Charles-Antoine Assenmacher, Dayo O. Adewole, Sai Panigrahy, Bita Soltan Mohammadlou, Lucy Plant, Yuan Zhang, Andrew G. Richardson, Yury Gogotsi, D. Kacy Cullen, Flavia Vitale**

Supplementary Notes

Note S1: Mass ratio of $Ti_3C_2T_x$:Ca^{2+} ions

Concentration of $Ti_3C_2T_x$ dispersion = 40 mg/mL

Concentration of $CaCl_2$ = 100 mM = 0.1 M

Concentration of Ca^{2+} ions (M_{Ca} = 40 g) = 0.1 M x 40 x 10^3 mg/mole x 10^{-3} L/mL = 4 mg/mL

When mixing equal volumes of $Ti_3C_2T_x$ dispersion and $CaCl_2$,

Mass ratio of $Ti_3C_2T_x$:Ca^{2+} ions = 40 x 10^{-3} mg/mL : 4 mg/mL = 10 : 1

Note S2: Physiological relevance of biphasic current pulses for continuous pulsing

Total charge per phase (Q) = 1 mA * 500 μs = 0.5 μC

Area of the electrode (A) = π * $(0.5\ \text{mm})^2$ = 0.00785 cm^2

Charge density (D) = Q / A = 63.7 μC/cm^2

Shannon equation, which describes the boundary between damaging and non-damaging electrical stimulation levels, is as follows:

$$log(D) = k - log(Q)$$

where, k is an adjustable parameter that defines the limits for safe stimulation such that $1.5<k<2.0$.[1, 2] Here,

$$k = \log(63.7) + \log(0.5) = 1.503$$

Supplementary Figures

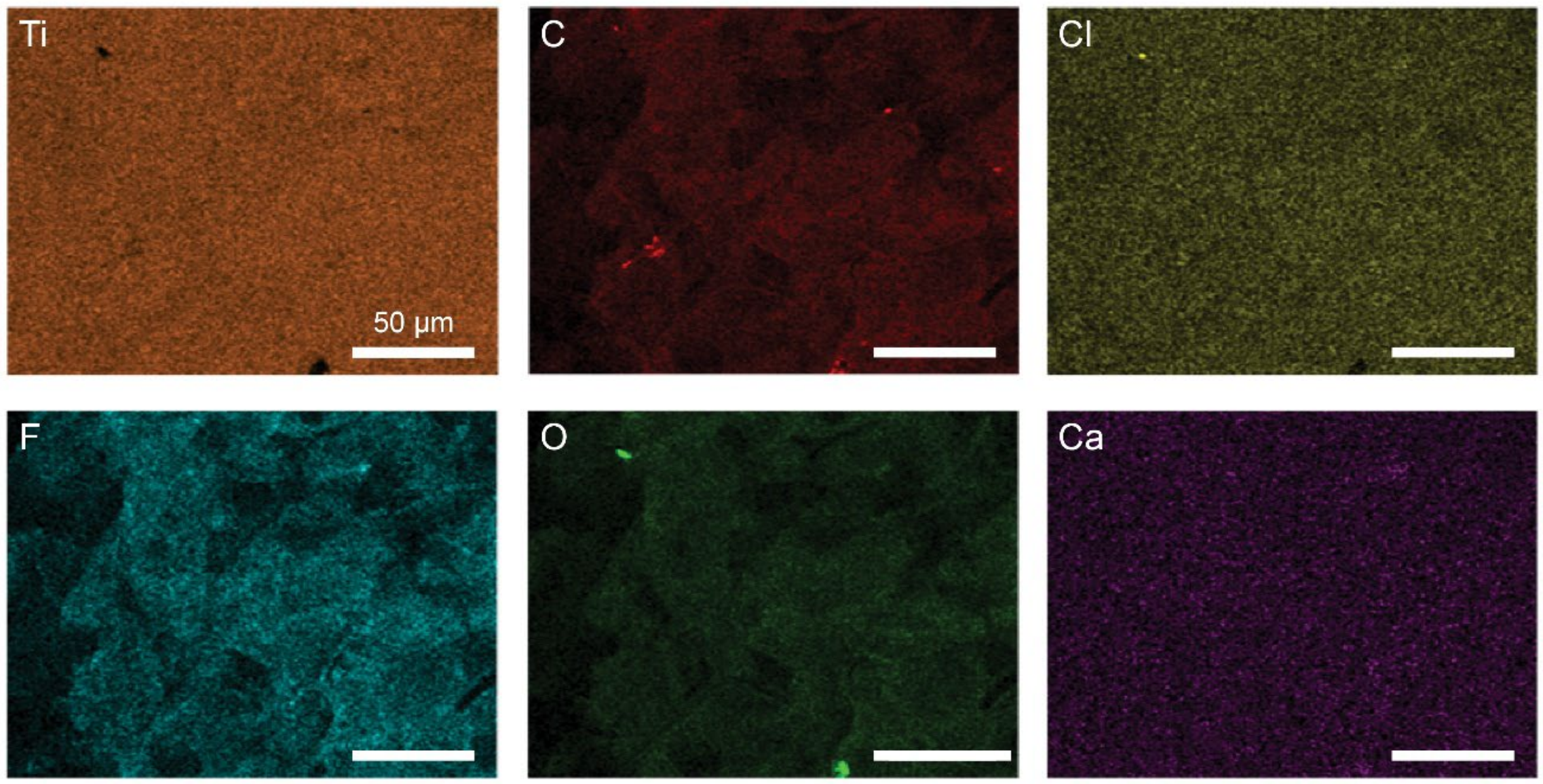

Figure S1. Characterization via energy dispersive X-ray spectroscopy (EDS). Elemental mappings of freeze-dried $Ti_3C_2T_x$ MXgel.

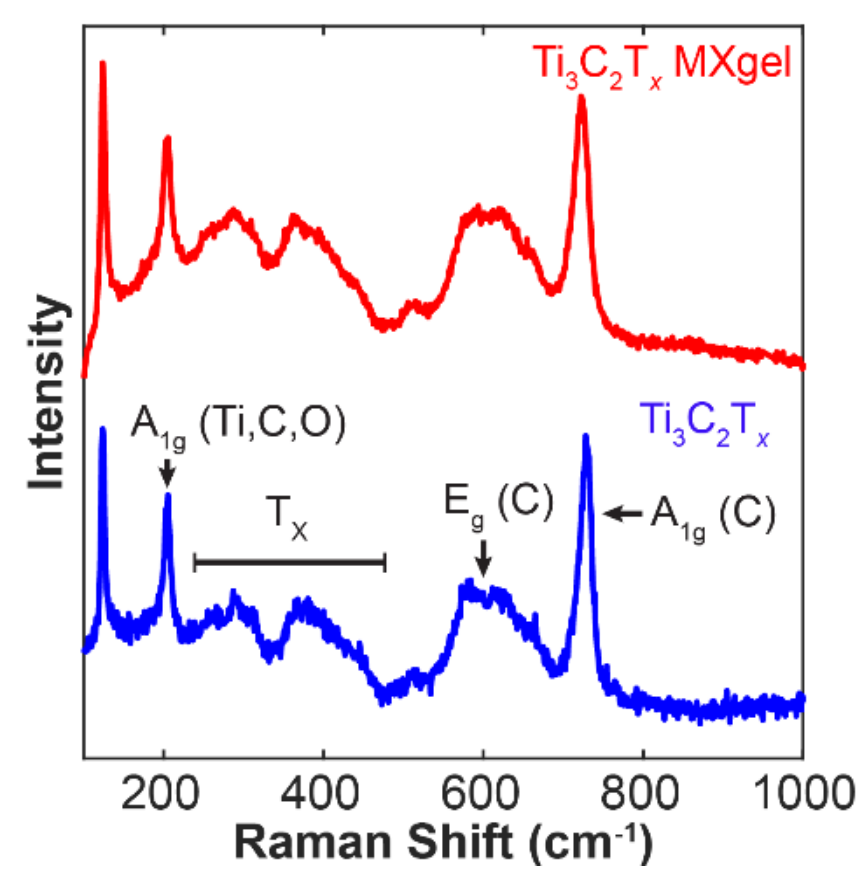

Figure S2. Raman spectroscopy. Representative Raman spectra of $Ti_3C_2T_x$ film (blue) and Ca^{2+} crosslinked $Ti_3C_2T_x$ MXgel (red).

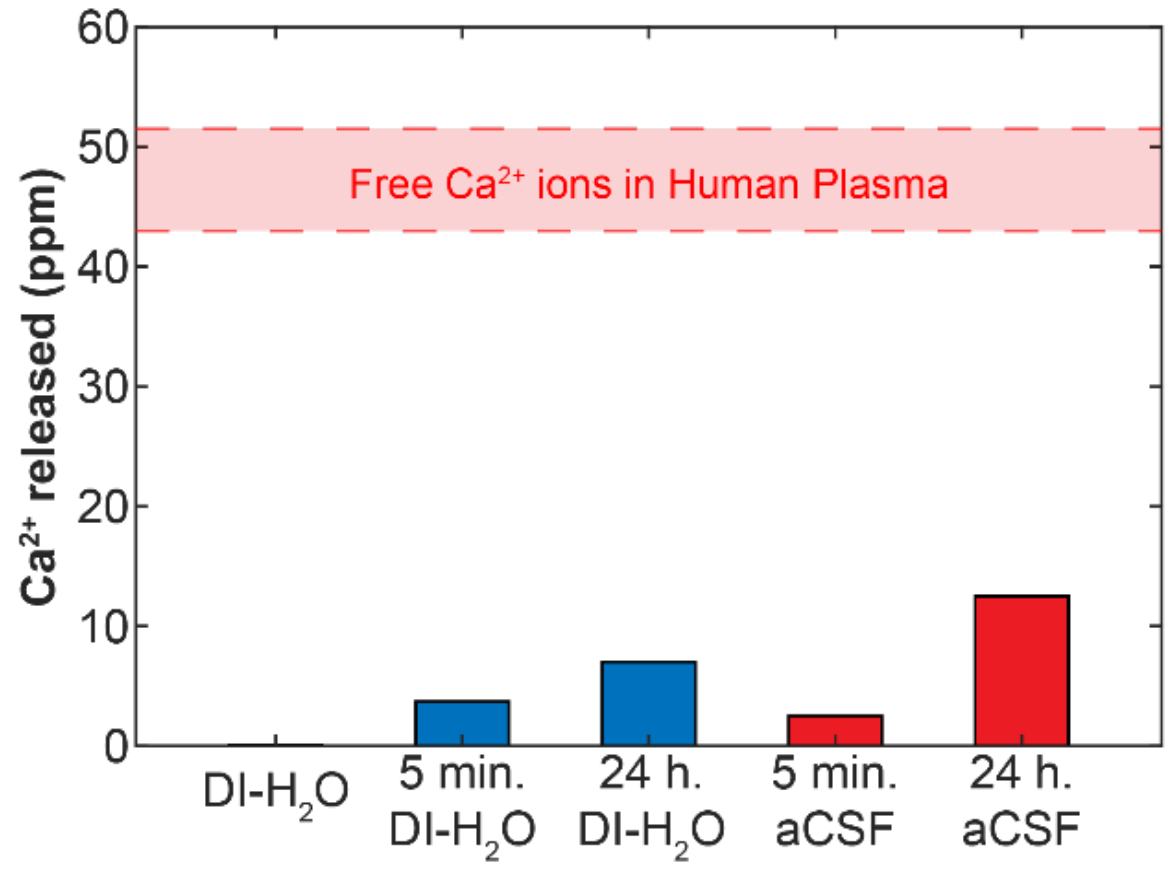

Figure S3. Ca^{2+} ions released from $Ti_3C_2T_x$ MXgel. Concentration of Ca^{2+} ions released in DI-H_2O and aCSF after MXgel samples were soaked for 5 min and 24 h at 37°C, measured using inductively coupled plasma optical emission spectroscopy.

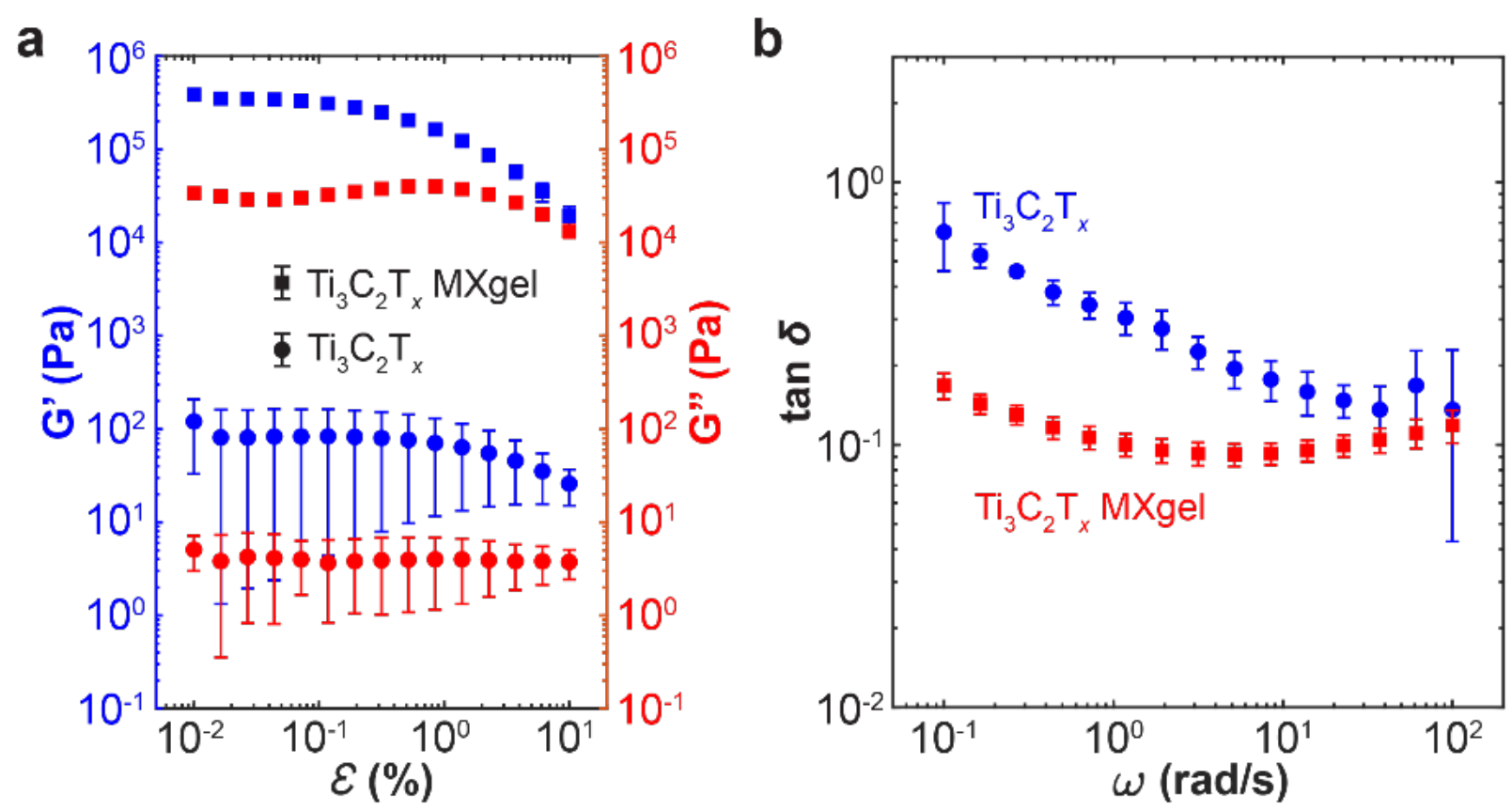

Figure S4. Viscoelastic properties of $Ti_3C_2T_x$ and MXgel. (a) Storage (*G*') and loss (*G*'') moduli as a function of strain amplitude ($\mathcal{E}$) for precursor $Ti_3C_2T_x$ dispersion and $Ti_3C_2T_x$ MXgel. **(b)** Tan δ as a function of angular frequency (ω) at 0.1% strain for precursor $Ti_3C_2T_x$ dispersion and crosslinked $Ti_3C_2T_x$ MXgel. Data presented as mean ± SD ($N = 3$).

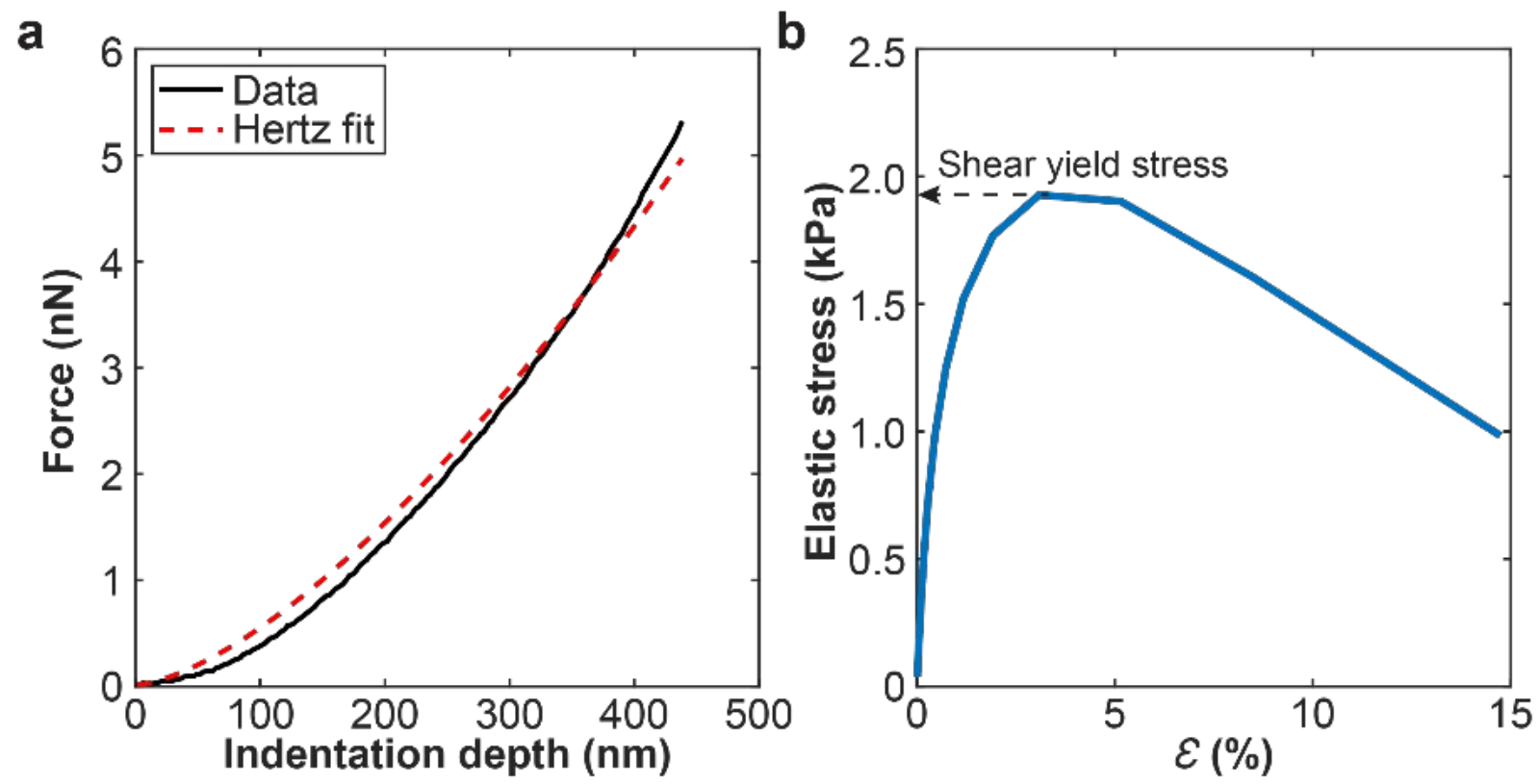

Figure S5. Mechanical properties of MXgel. (a) Force measured by the AFM tip on the surface of a representative MXgel sample as a function of indentation depth ($N = 3$). Black and red dashed lines denote the data and the Hertz model fit, respectively. **(b)** Elastic stress as a function of strain amplitude ($\mathcal{E}$) for a representative $Ti_3C_2T_x$ MXgel ($N = 3$). Red, orange, and blue plots denote individual MXgel samples.

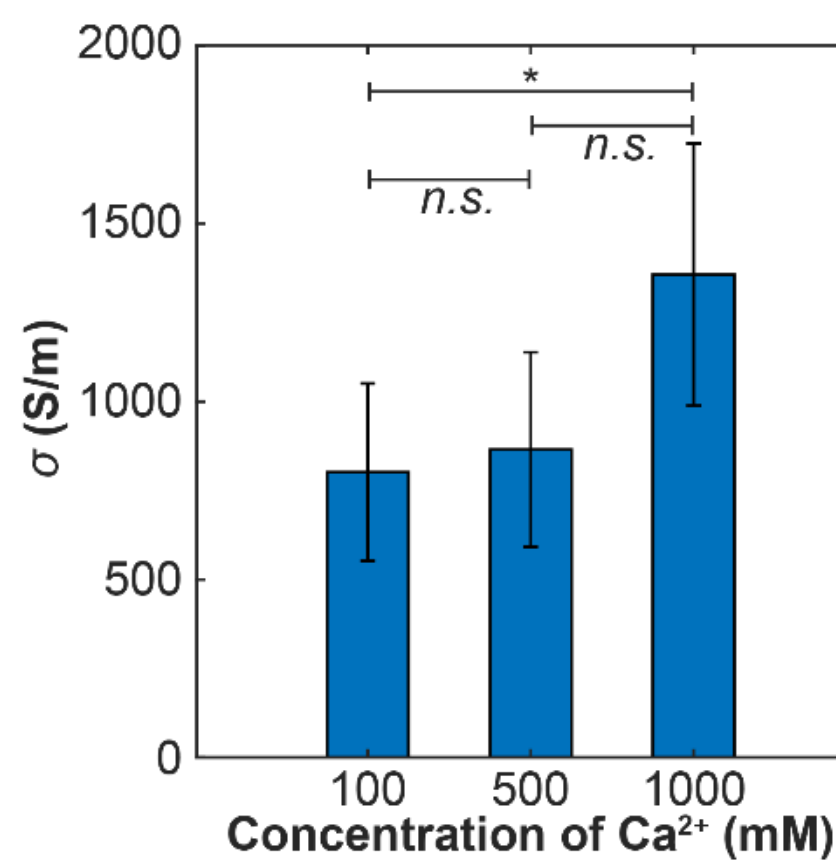

Figure S6. Electrical properties of MXgel with varying Ca^{2+} crosslinker. Electrical conductivity (σ) of MXgel formulated with varying concentrations of Ca^{2+} ions. Data presented as mean ± SD ($N = 5$). *p<0.05, one-way ANOVA with post-hoc Tukey (n.s. – not significant).

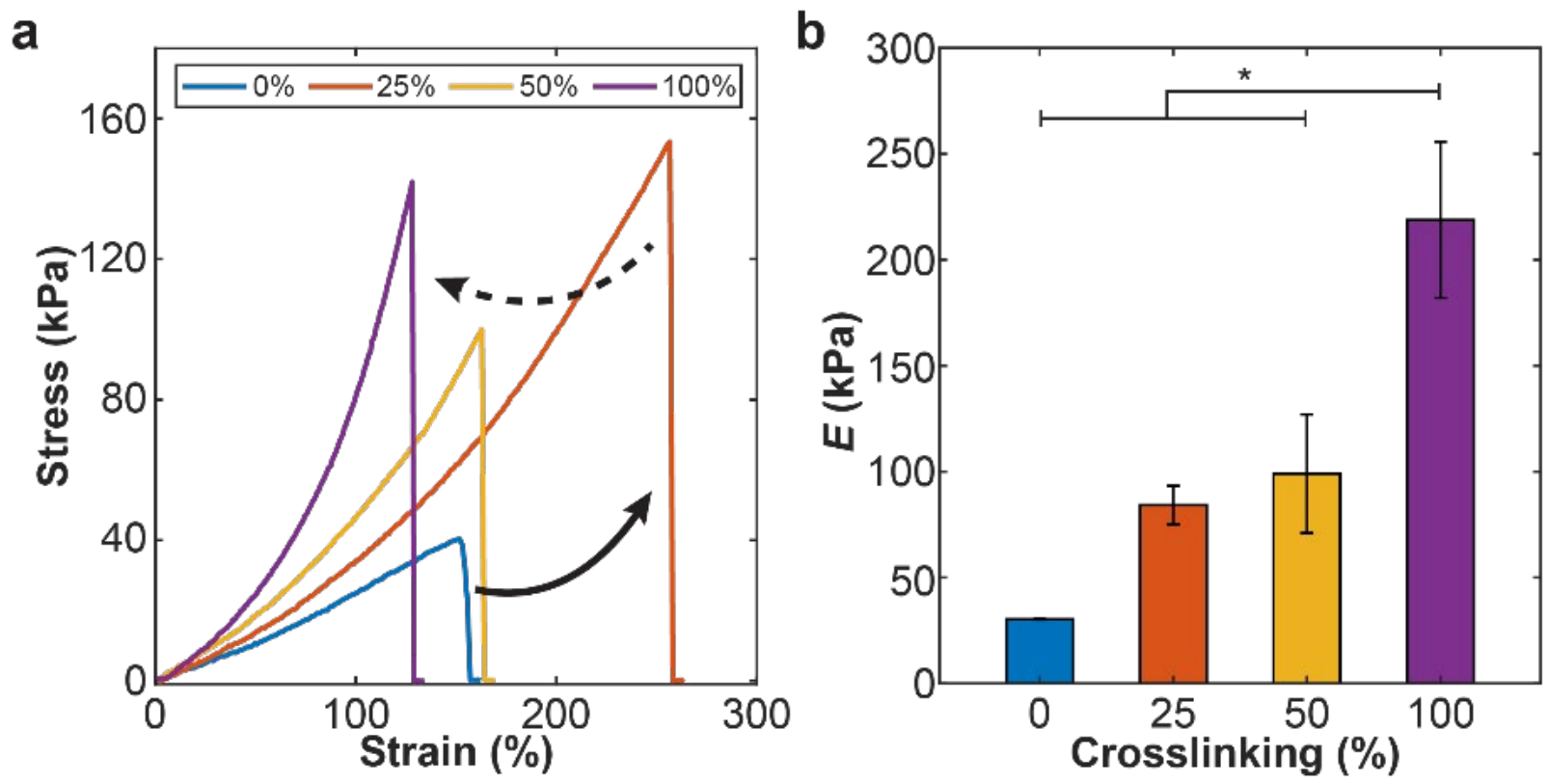

Figure S7. Mechanical properties of gelapin. (a) Representative stress-strain curves of gelapin with increasing degree of crosslinking ($N = 3$) **(b)** Young's modulus (E) as a function of gelapin crosslinking. Data presented as mean ± SD ($N = 3$). *$p<0.05$, one-way ANOVA with post-hoc Tukey.

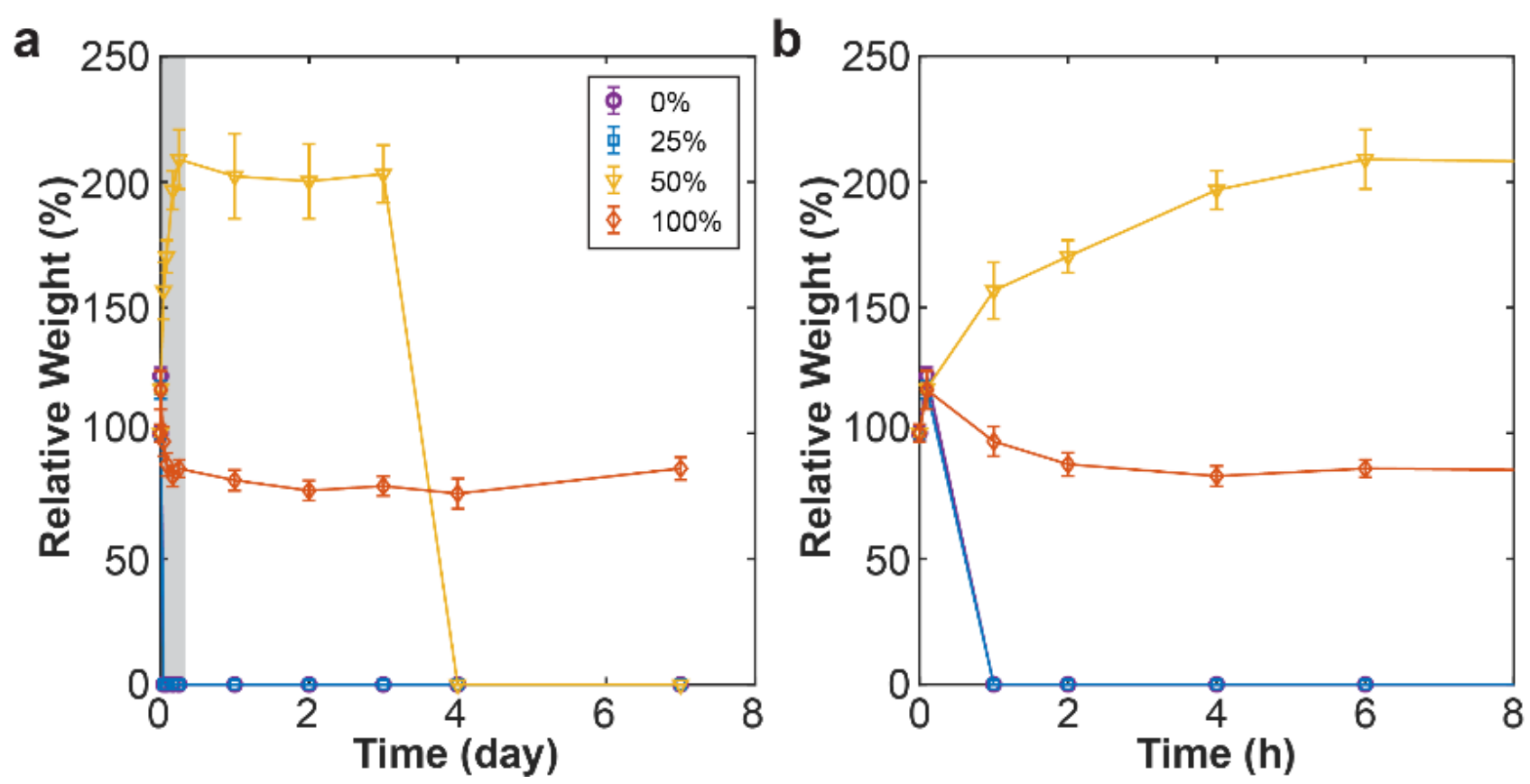

Figure S8. Degradation of gelapin in physiological conditions. (a) Relative weight of gelapin with varying degrees of crosslinking as measured over **(a)** days in 1X PBS at 37°C and **(b)** first 8 hours (shaded region in (a)). Data presented as mean ± SD ($N = 5$).

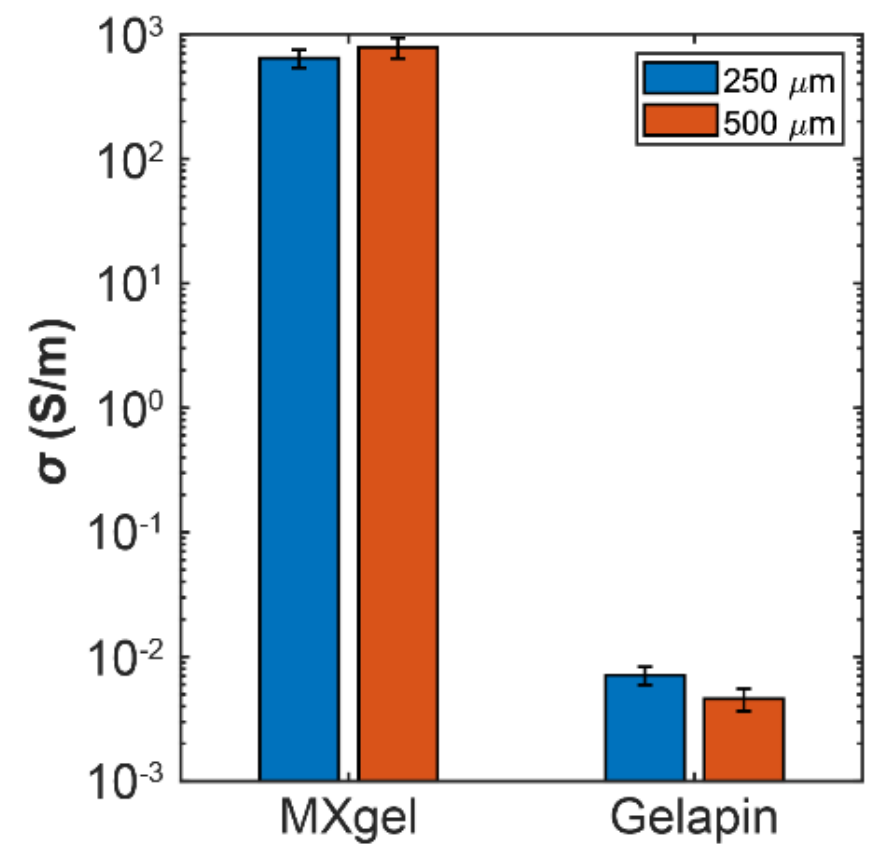

Figure S9. Electrical conductivity of MXgel and gelapin. Electrical conductivity (σ) of 250 and 500 µm thick MXgel and gelapin hydrogels measured via four-point probe. Data presented as mean ± SD ($N = 5$).

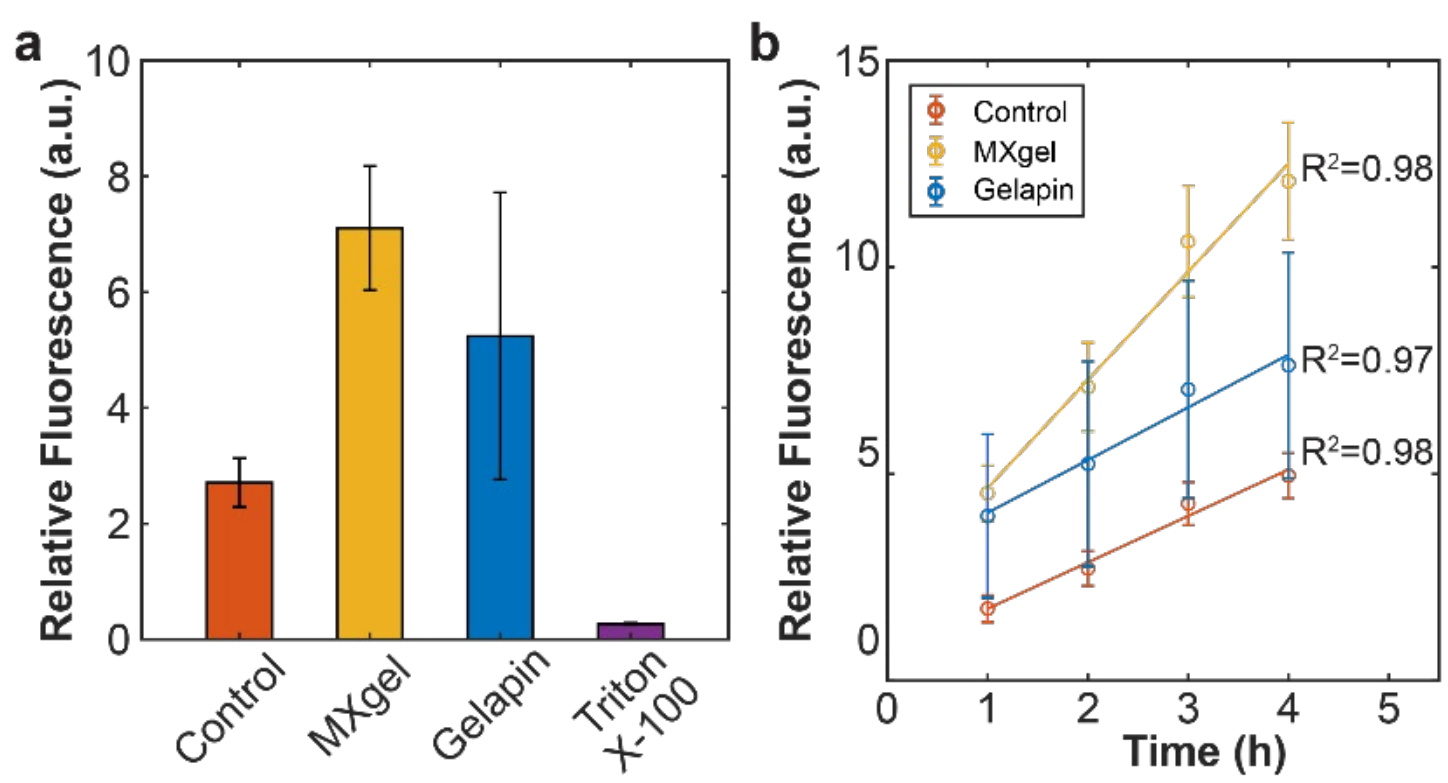

Figure S10. Cytotoxicity screening and metabolic activity of astrocytes exposed to hydrogel-conditioned media extracts. (a) Background-corrected resorufin fluorescence measured 2 h after the addition of alamarBlue to rat astrocytes incubated in media extracted from different samples (control, MXgel, Gelapin, and 0.05% Triton X-100). **(b)** Background-corrected resorufin fluorescence kinetics of resorufin measured over time (1-4 h) post-alamarBlue addition for blank extract (control), MXgel extract, and Gelapin extract. Data presented as mean ± SD ($N = 4$).

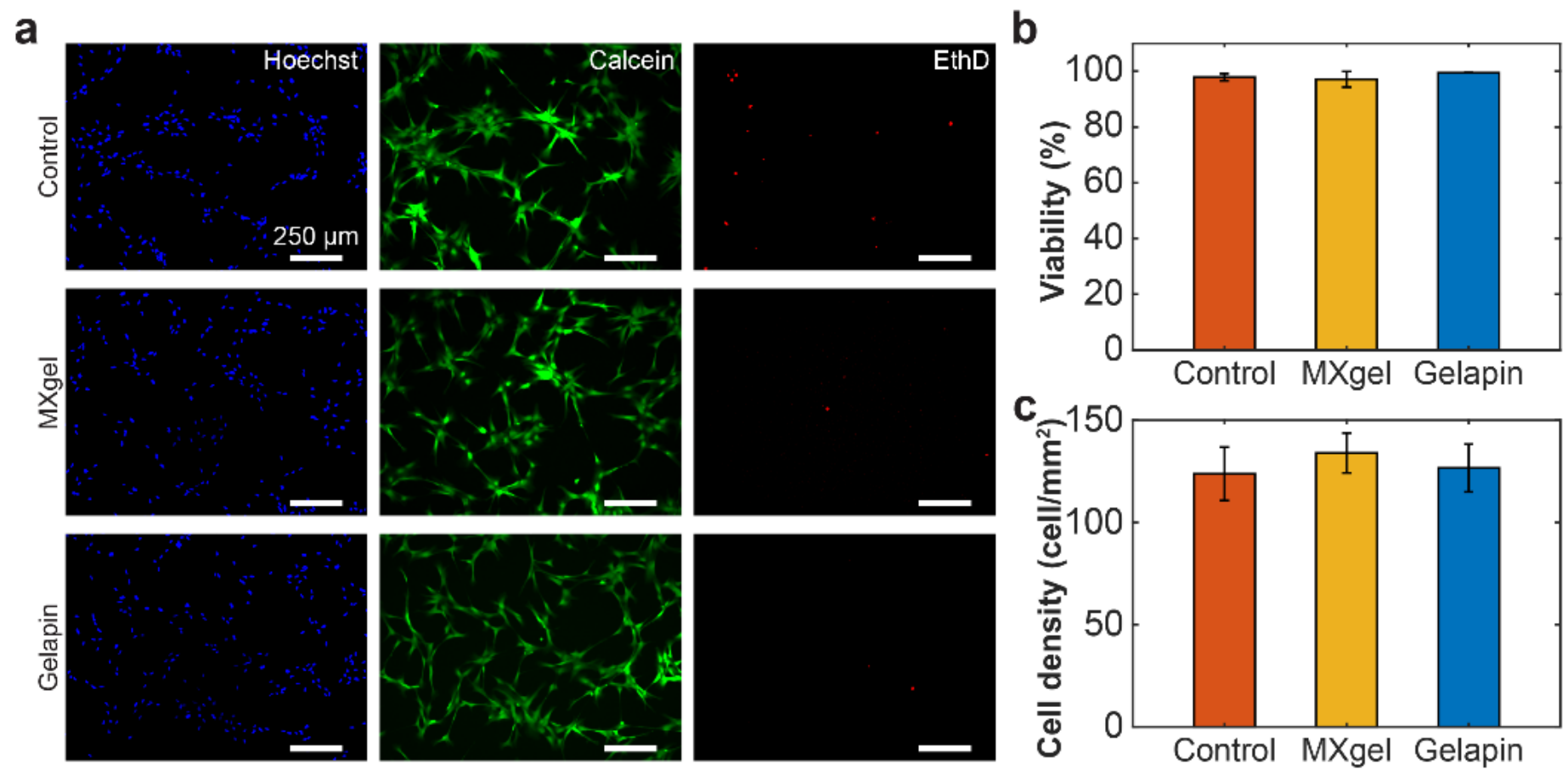

Figure S11. Viability of astrocytes exposed to hydrogel-conditioned media extracts. (a) Representative fluorescence images of Hoechst-stained nuclei (blue), Calcein AM-positive live cells (green), and EthD-1-positive dead cells (red) following 24 h exposure to control (blank), MXgel, and Gelapin extracts. **(b)** Quantification of astrocyte viability following exposure to Control, MXgel, and Gelapin extracts. **(c)** Quantification of cell density from Hoechst-positive nuclei of astrocytes exposed to Control, MXgel, and Gelapin extracts. Data presented as mean ± SD ($N = 4$).

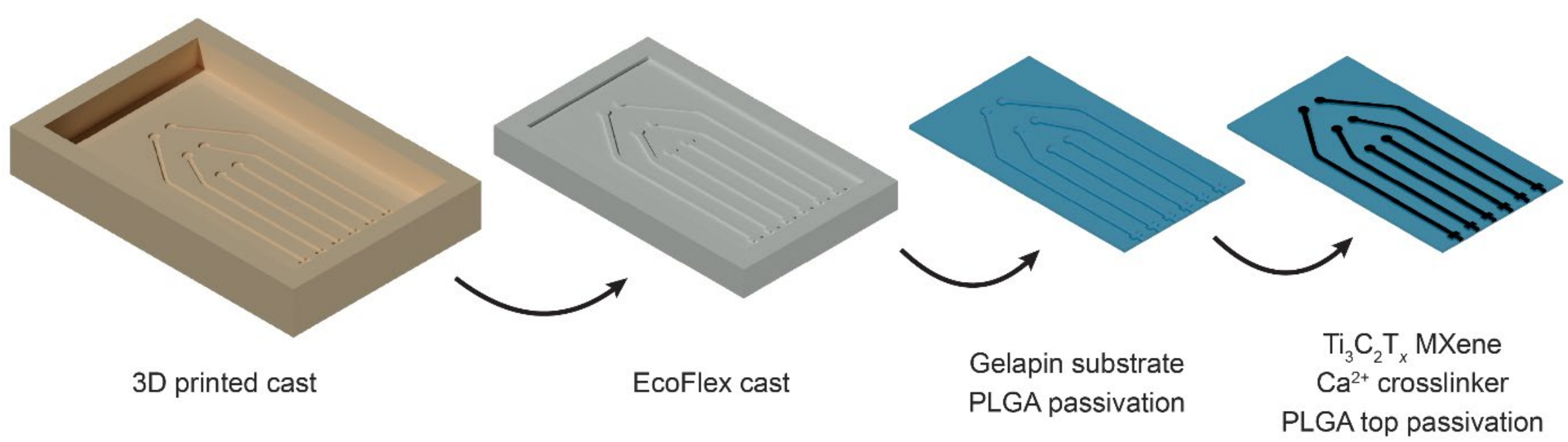

Figure S12. Fabrication protocol. Illustration showing the steps involved in the fabrication of MXgel bioelectronics.

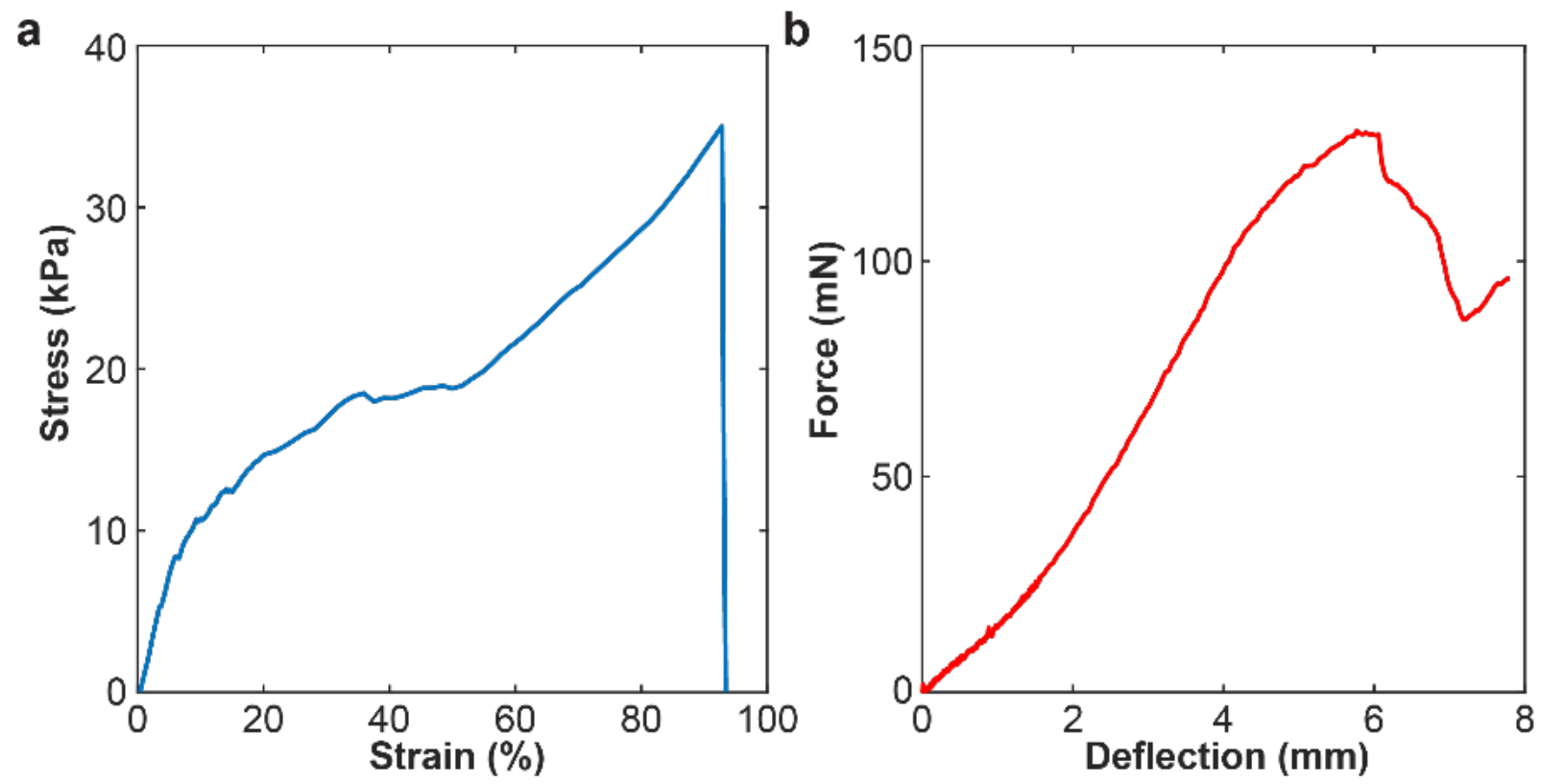

Figure S13. Mechanical characterization of MXgel device structures. (a) Stress as a function of tensile strain for a representative hydrated MXgel device structure (*N* = 4). **(b)** Force as a function of deflection under 3-point bending for a representative hydrated MXgel device structure (*N* = 4).

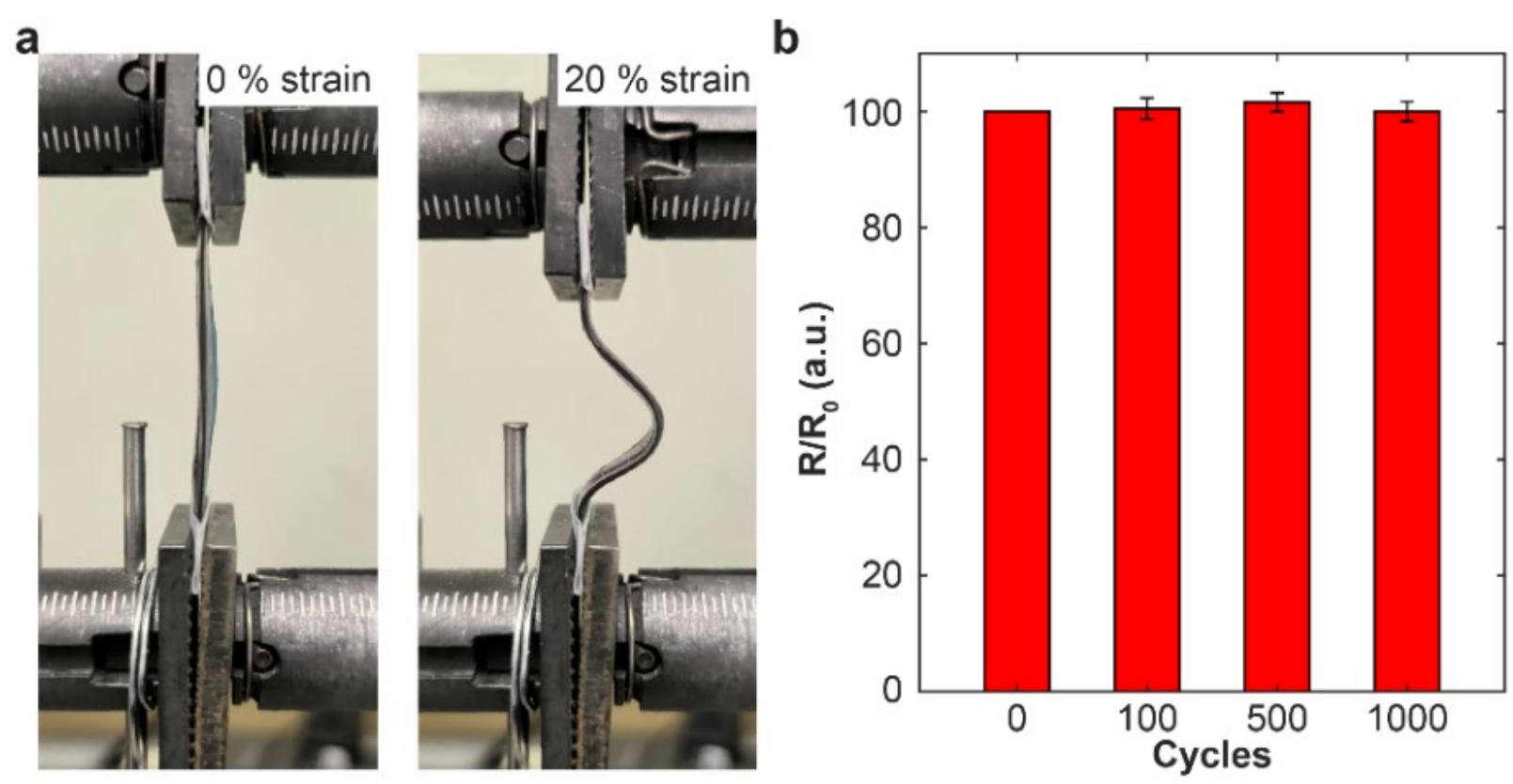

Figure S14. Electrical properties of MXgel under repeated bending. (a) Photographs of MXgel on gelapin substrates at 0% and 20% strain. **(b)** Relative resistance of MXgel on gelapin as a function of bending cycles under 20% strain (maximum) at a rate of 20%/s. Data presented as mean ± SD (*N* = 6). No statistical difference, one-way ANOVA ($p = 0.19$).

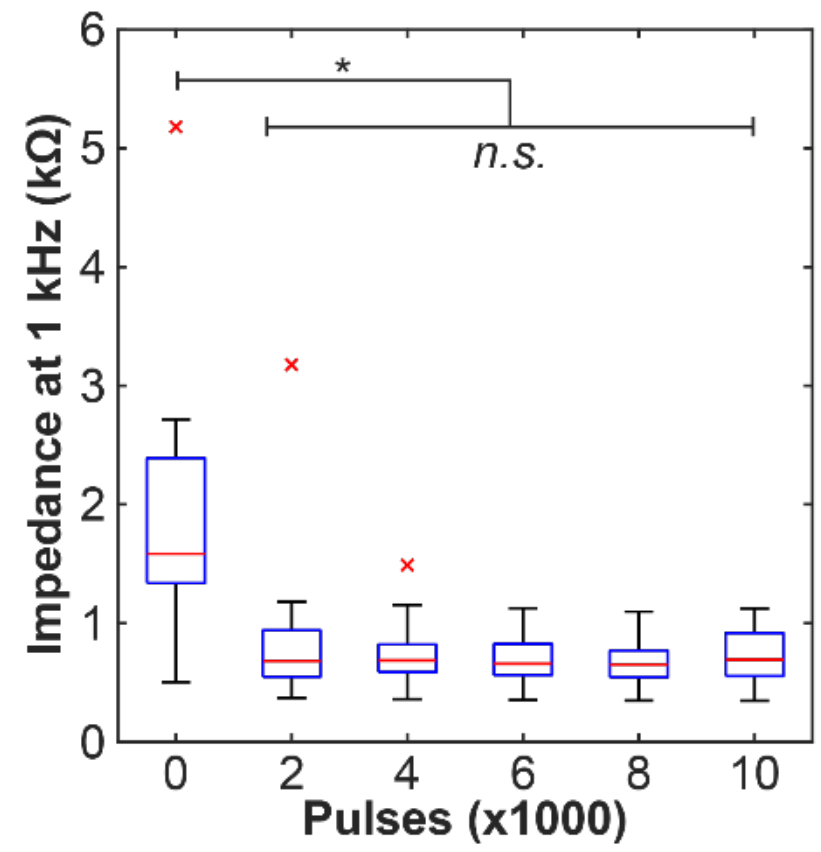

Figure S15. Electrochemical stability of MXgel bioelectronics under continuous current pulsing. Electrochemical impedance at 1 kHz measured for 1 mm MXgel electrodes under continuous biphasic current pulses with 1 mA – 500 µs cathodic phase ($N = 10$). Central mark, bottom edge, top edge, whiskers, and 'x' denote median, 25th percentile, 75th percentile, most extreme data points, and outliers, respectively. *$p<0.05$, one-way ANOVA with post-hoc Tukey (n.s. – not significant).

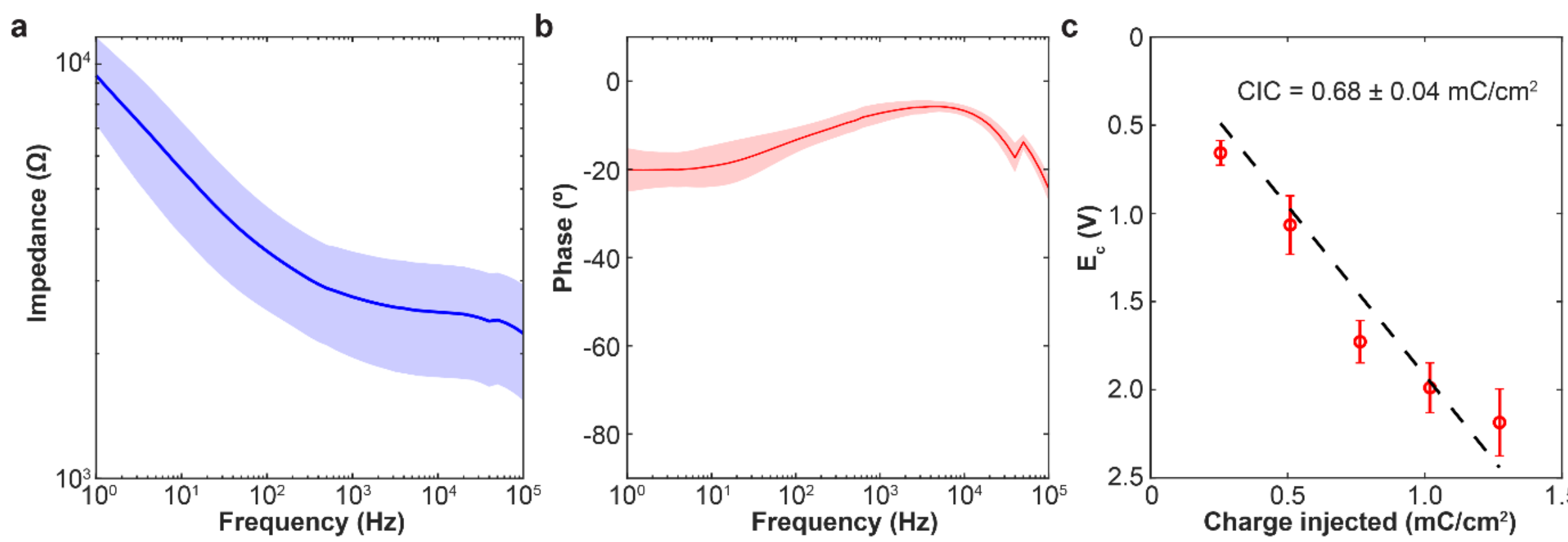

Figure S16. Electrochemical properties of MXgel electrodes with diameter of 500 µm. Bode plot of **(a)** impedance modulus and **(b)** phase for 500 µm MXgel electrodes. Solid line and shaded region denote mean and standard deviation, respectively ($N = 6$ electrodes across 3 devices). **(c)** Cathodic potential excursion (E_c) as a function of injected charge for 500 µm MXgel electrodes. Data presented as mean ± SD ($N = 6$ electrodes across 5 devices).

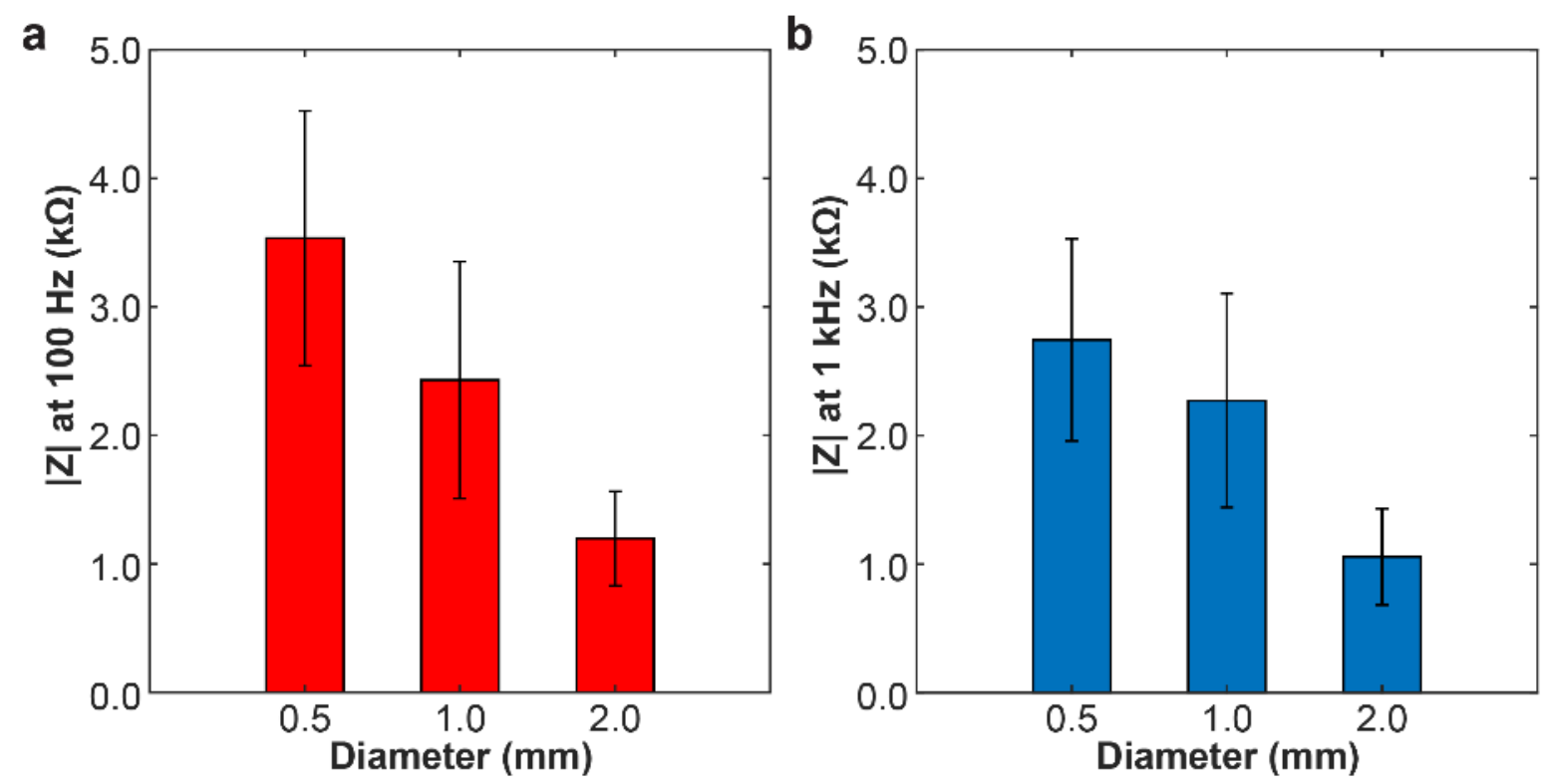

Figure S17. Electrochemical impedance as a function of the electrode diameter. Impedance (|Z|) at **(a)** 100 Hz and **(b)** 1 kHz for 0.5 mm, 1 mm, and 2 mm electrodes. Data presented as mean ± SD (N = 6-20 electrodes).

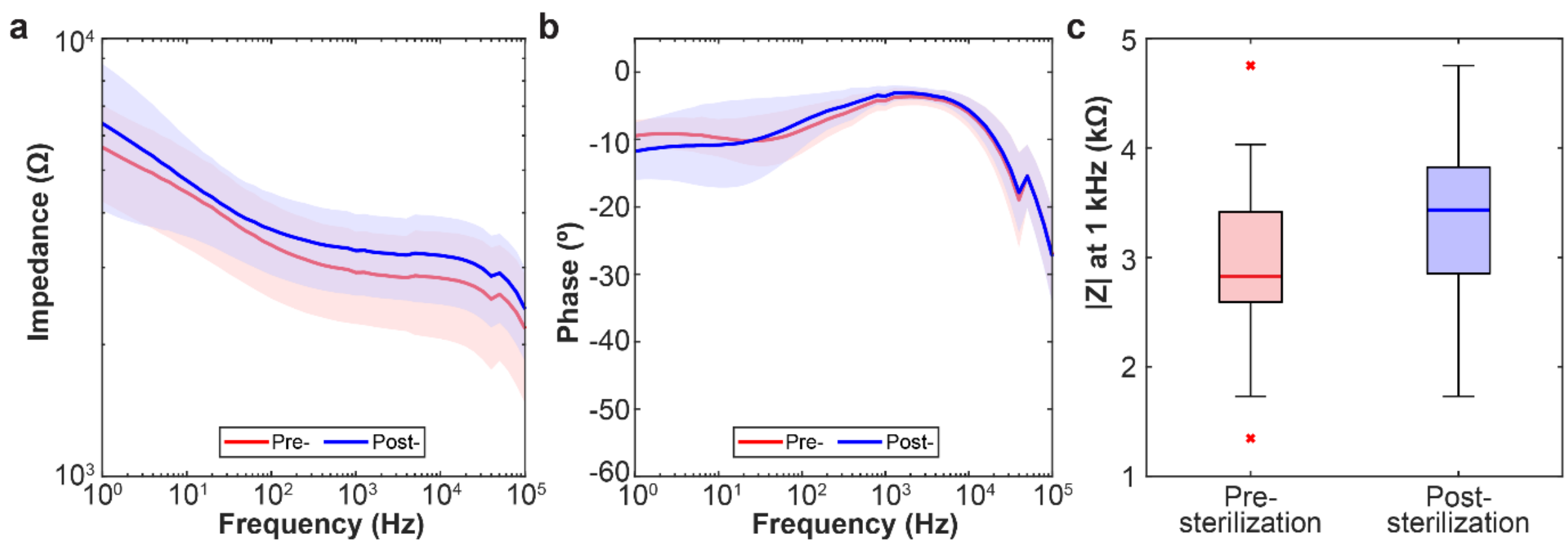

Figure S18. EtO sterilization does not affect MXgel impedance. (a) Impedance modulus and **(b)** phase for 1 mm MXgel electrodes pre- and post-EtO sterilization. Solid line and shaded region denote mean and standard deviation, respectively (N = 27 electrodes across 5 devices). **(c)** Box plot presenting impedance (|Z|) at 1 kHz for 1 mm MXgel electrodes pre- and post-sterilization (N = 27 electrodes across 5 devices). Central mark, bottom edge, top edge, whiskers, and 'x' denote median, 25th percentile, 75th percentile, most extreme data points, and outliers, respectively. No statistical difference, one-way ANOVA (p = 0.07).

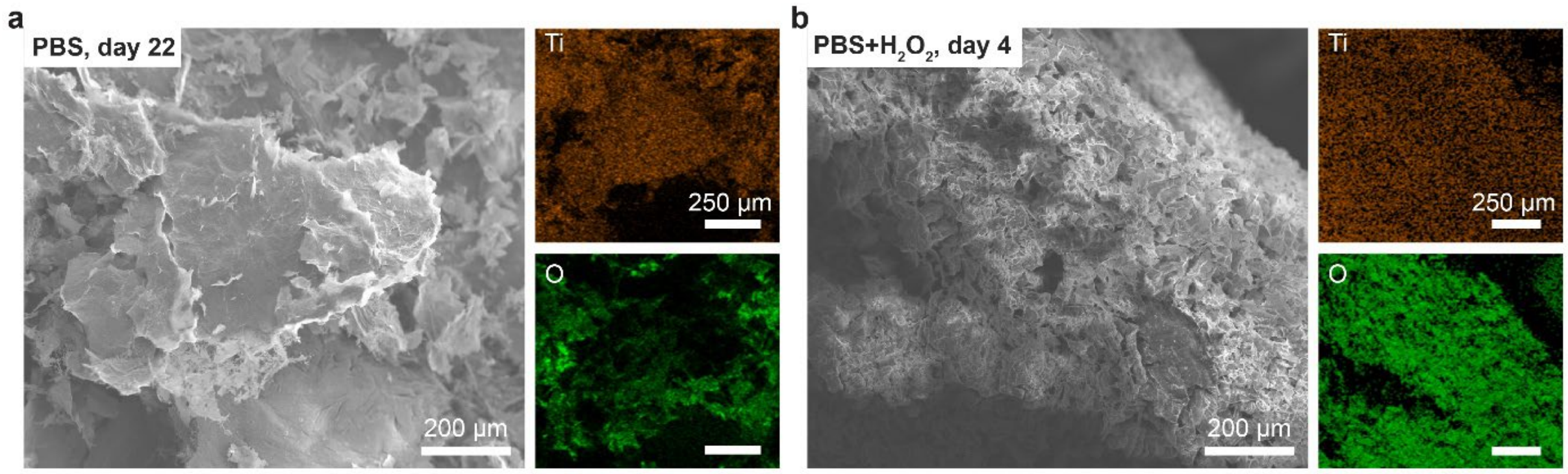

Figure S19. Hydrolysis of MXgel under physiological conditions. Scanning electron microscopy images and related EDS elemental mappings (Ti and O) of representative MXgel stored in **(a)** 1X PBS and **(b)** 1X PBS with 30 mM H_2O_2 over multiple days (N = 4 samples per condition).

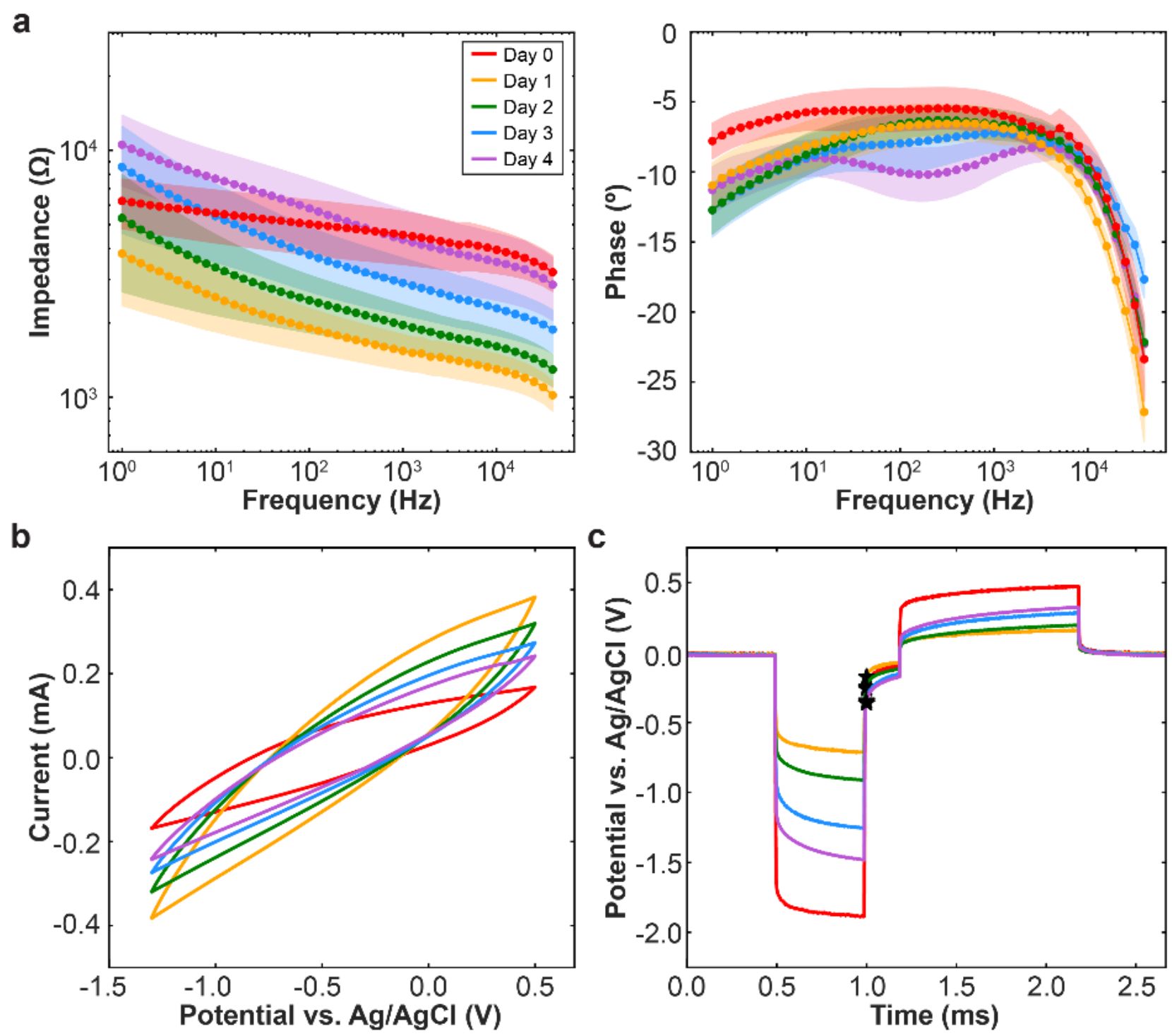

Figure S20. Electrochemical performance of functional MXgel electrodes over 4 days. (a) Impedance (left) and phase (right) as a function of frequency for 1 mm MXgel electrodes stored in 1X PBS at 37°C. Data presented as mean ± SD (*N* = 30 electrodes across 5 devices on day 0). **(b)** Cyclic voltammograms acquired at 250 mV/s for functional 1 mm MXgel electrodes. Data presented as the mean of the 5th cyclic voltammograms across all functional electrodes. **(c)** Voltage transients (VT) measured under 0.5 mA, 0.5-ms cathodic current, 0.1-ms inter-pulse duration, and 0.25 mA, 1.0-ms anodic current pulses for functional 1 mm MXgel electrodes. Data presented as the mean of the 5th VT scan across all functional electrodes. Black stars denote cathodic potential excursion.

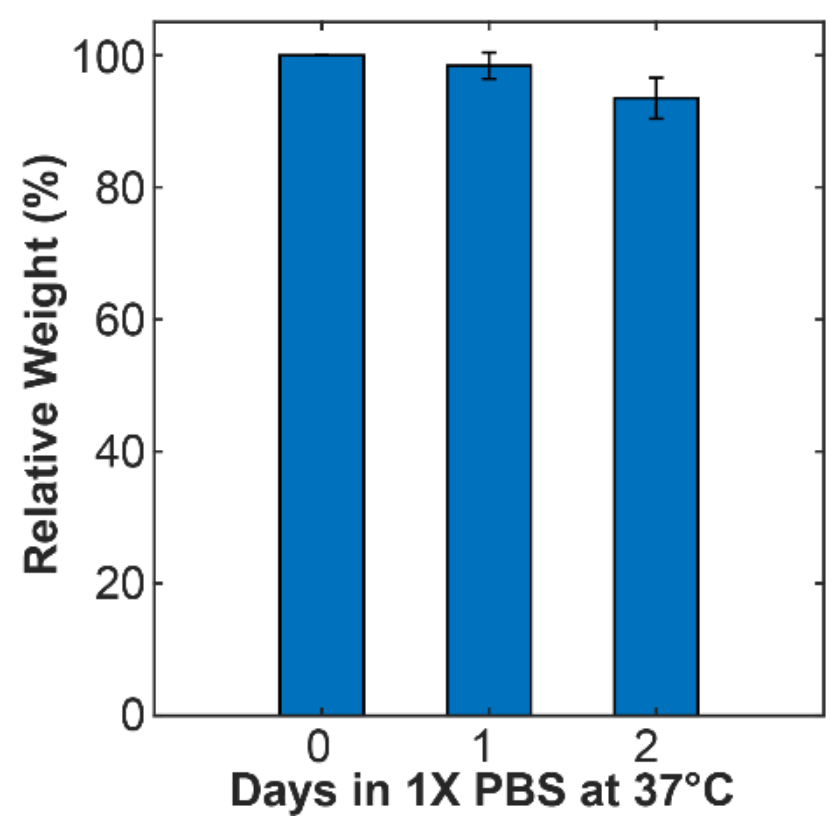

Figure S21. Swelling behavior of MXgel. Relative weight of MXgel as measured over days in 1X PBS at 37°C. Data presented as mean ± SD (N = 5).

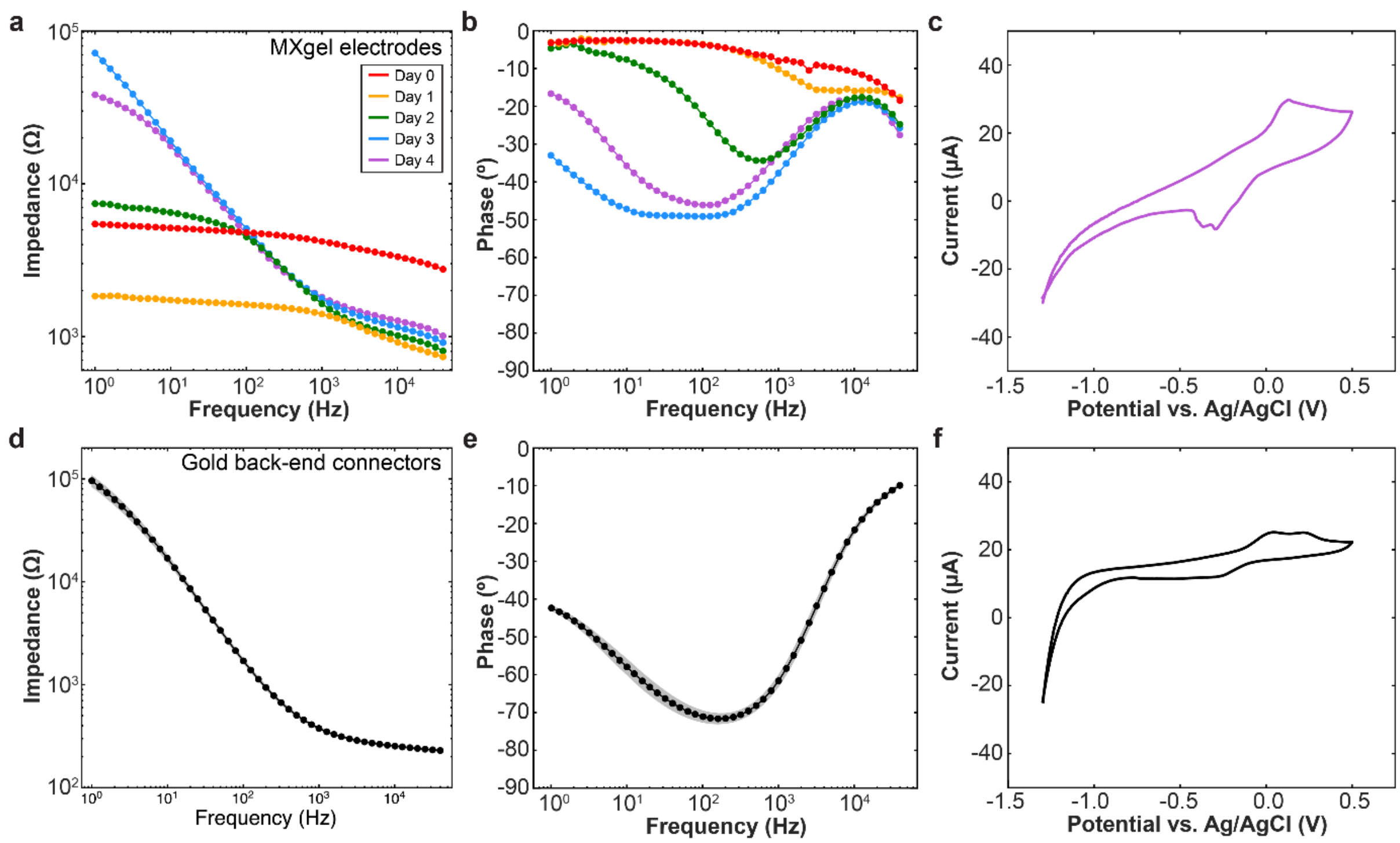

Figure S22. Electrochemical characterization of failed MXgel electrodes. (a) Impedance and **(b)** phase as a function of frequency for a representative MXgel electrode as it fails over multiple days in 1X PBS at 37°C. **(c)** Representative cyclic voltammogram acquired at 250 mV/s scan rate for a MXgel electrode as it failed after 4 days in 1X PBS at 37°C. **(d)** Impedance and **(e)** phase as a function of frequency for gold-plated backend connectors. Data presented as mean ± SD (N = 6). **(f)** Average of the 5th cyclic voltammograms acquired at 250 mV/s scan rate for gold-plated backend connectors.

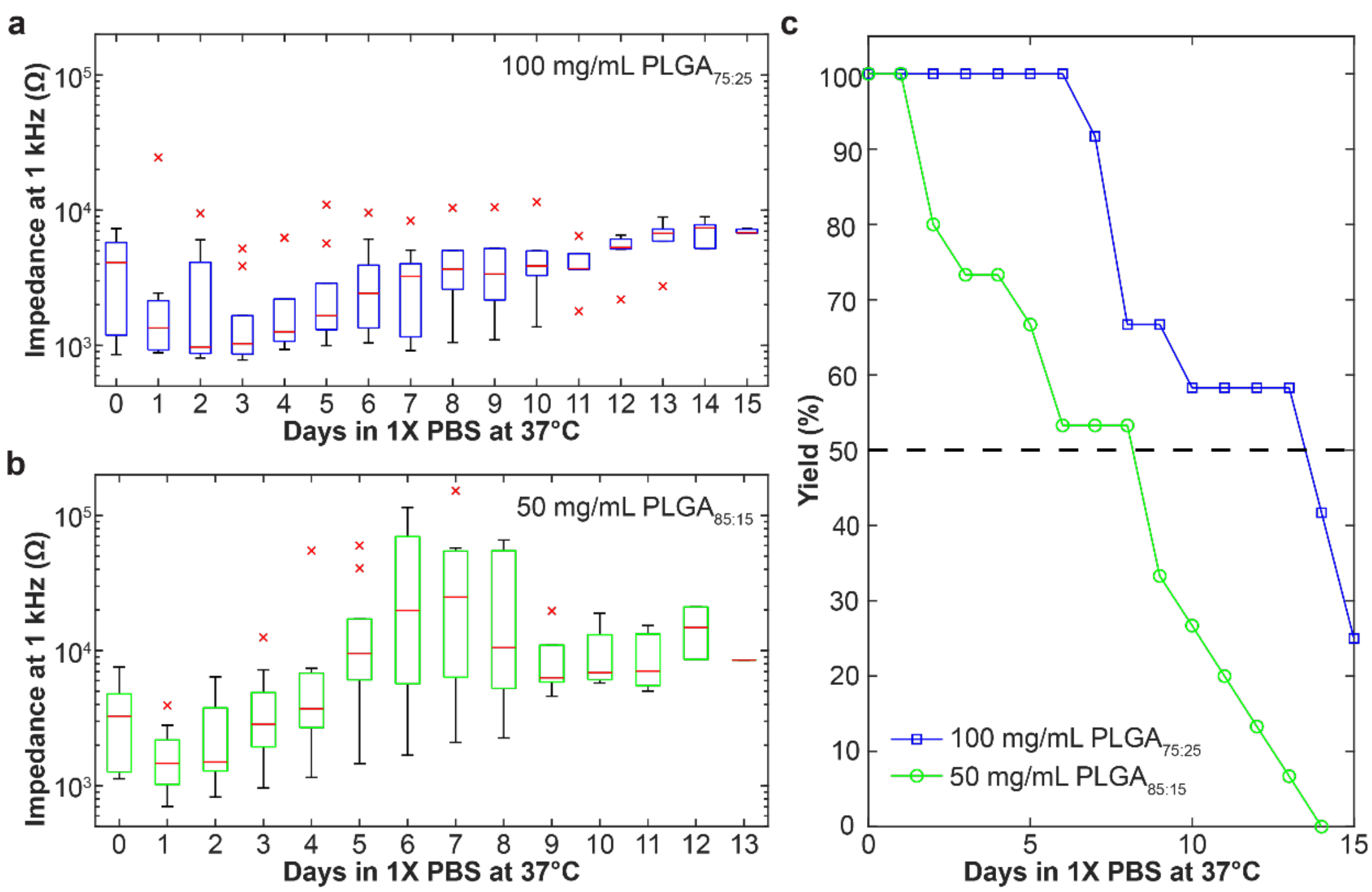

Figure S23. Tailoring functional lifetime of MXgel bioelectronics. Electrochemical impedance at 1 kHz as a function of days in 1X PBS at 37°C measured for functional 1 mm MXgel electrodes fabricated with **(a)** 100 mg/mL of $PLGA_{75:25}$ and **(b)** 50 mg/mL of $PLGA_{85:15}$ (N = 12-15 electrodes on day 0). Central mark, bottom edge, top edge, whiskers, and 'x' denote median, 25th percentile, 75th percentile, most extreme data points, and outliers, respectively. **(c)** Yield of functional 1 mm MXgel electrodes as a function of days in 1X PBS at 37°C.

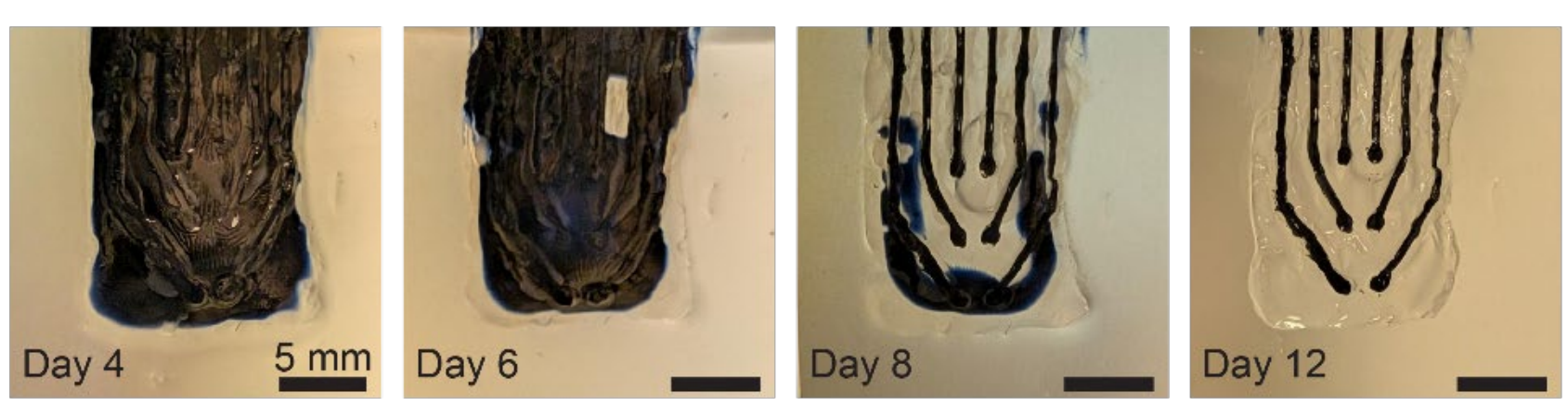

Figure S24. Simulated degradation of MXgel bioelectronics. Macroscopic images of MXgel devices prepared with 100 mg/mL of PLGA and stored under physiological conditions of 1X PBS at 37°C.

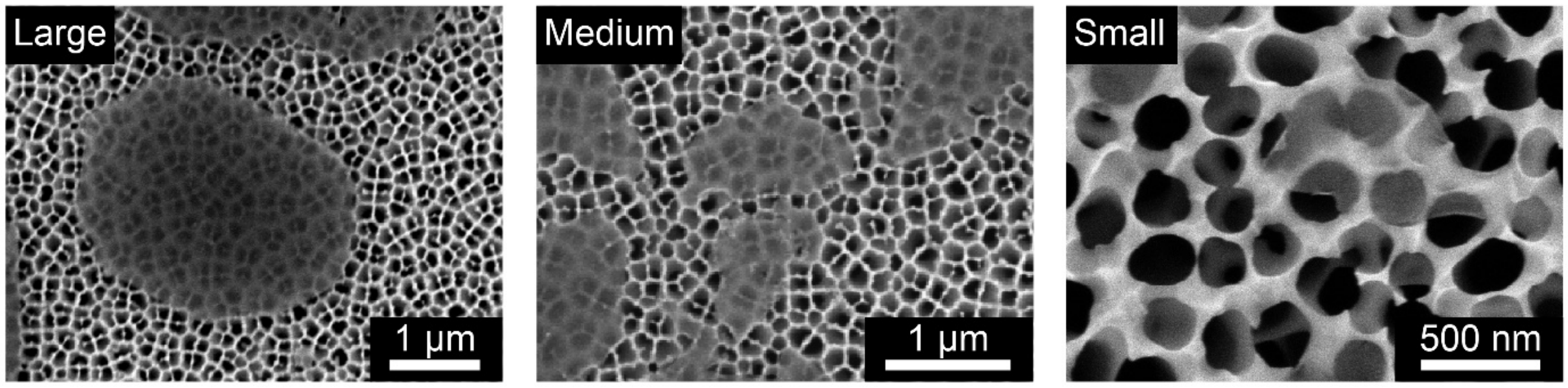

Figure S25. $Ti_3C_2T_x$ flakes of different sizes. Representative SEM images of large, medium, and small-size $Ti_3C_2T_x$ flakes.

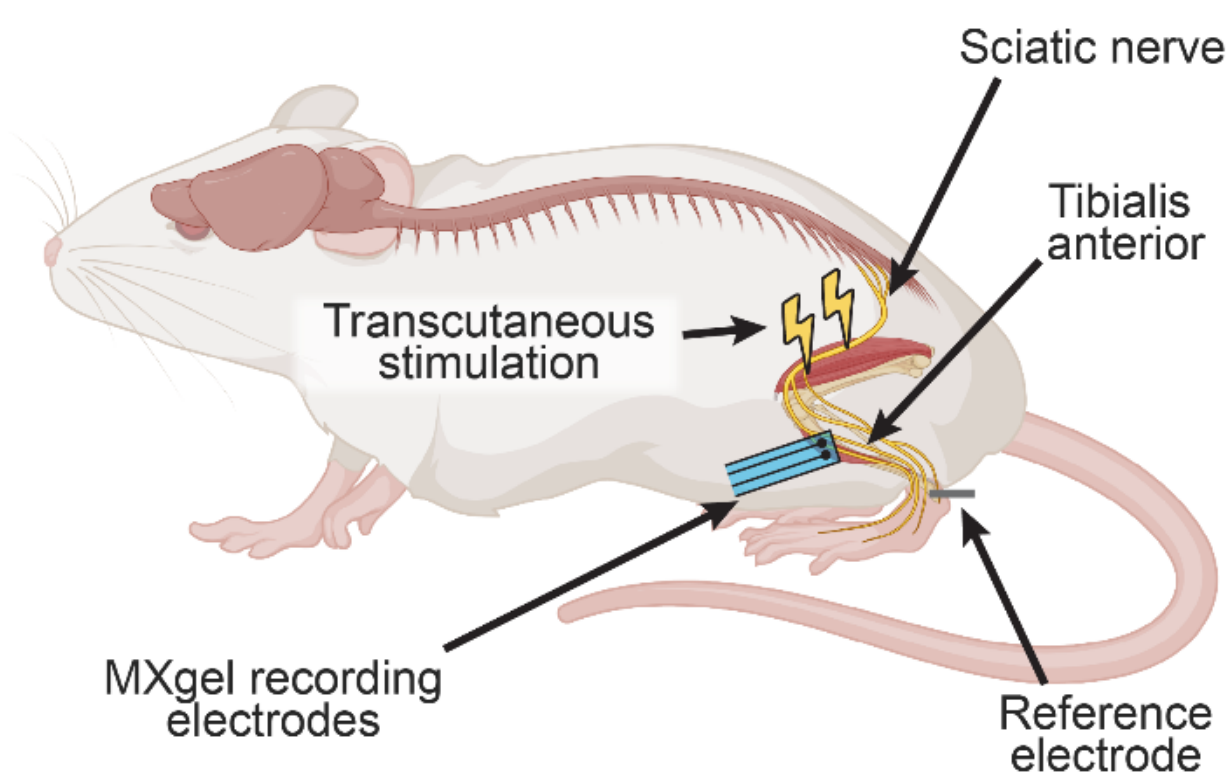

Figure S26. *In vivo* PNS recordings. Schematic illustrating the implantation of MXgel recording electrodes over the tibialis anterior and transcutaneous stimulation of the *sciatic nerve*.

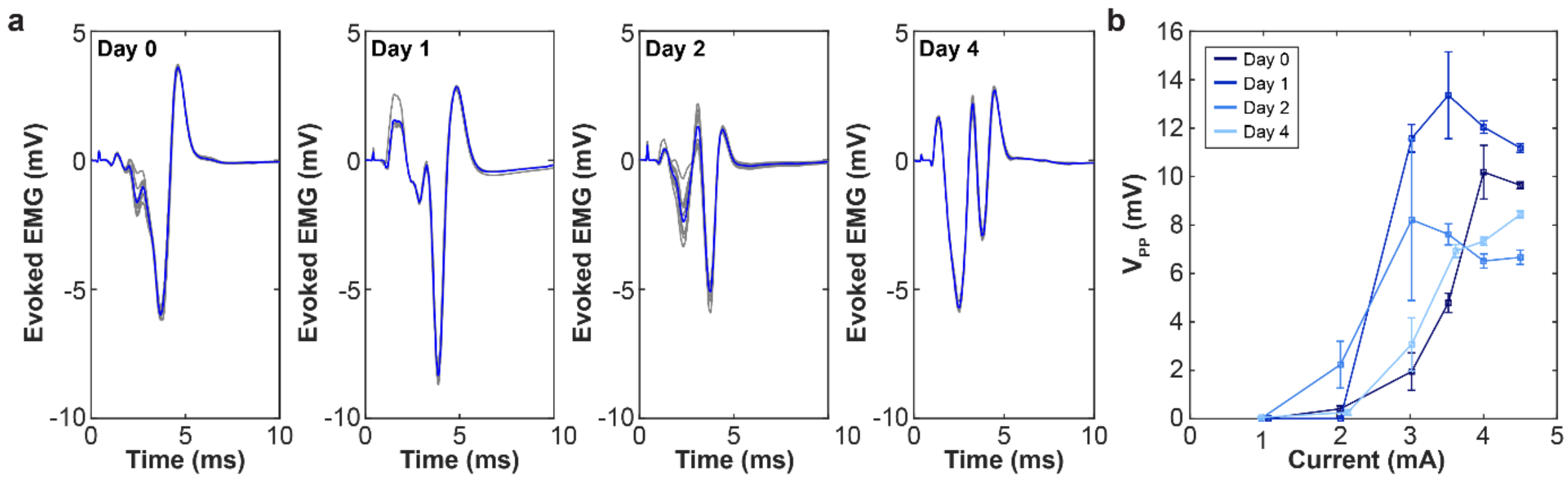

Figure S27. Evoked EMG recorded using MXgel electrodes over 4 days in 2nd rat. (a) Evoked EMG recorded at the *tibialis anterior* using a 1 mm MXgel electrode under transcutaneous stimulation of the *sciatic nerve* with 4.5 mA pulses. Gray and colored plots denote the individual pulses (*N* = 10) and the average of individual traces, respectively. **(b)** Peak-to-peak potential as a function of transcutaneous stimulation current. Dark to light colors denote responses recorded from day 0 to day 4. Data presented as mean ± SD (*N* = 10 pulses per stimulation current amplitude).

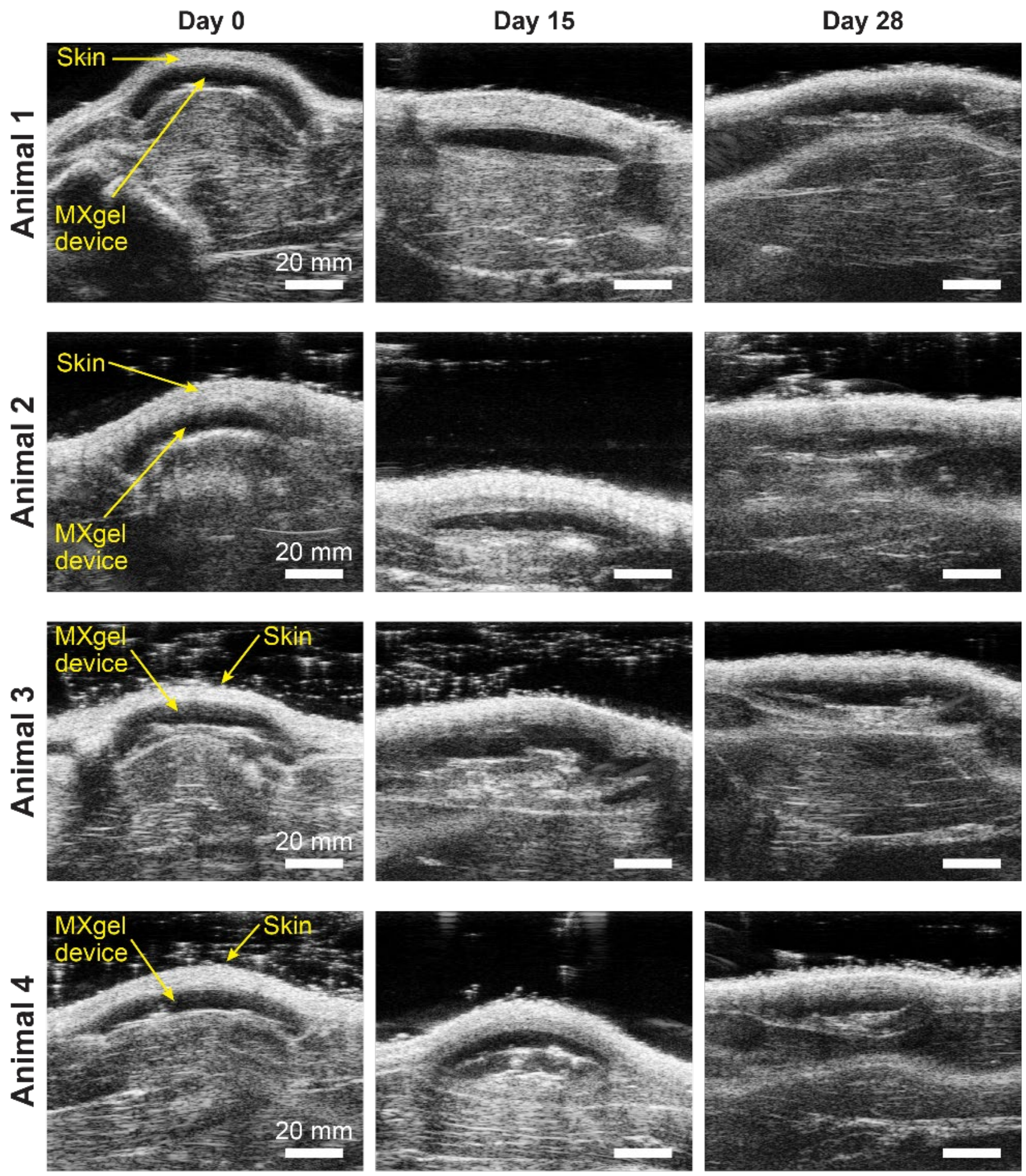

Figure S28. ***In vivo*** **biodegradation of MXgel bioelectronics over 4 weeks.** Representative ultrasound images of the *biceps femoris* before and after implantation of MXgel bioelectronics at days 0, 15, and 28 (terminal day) for 4 animals.

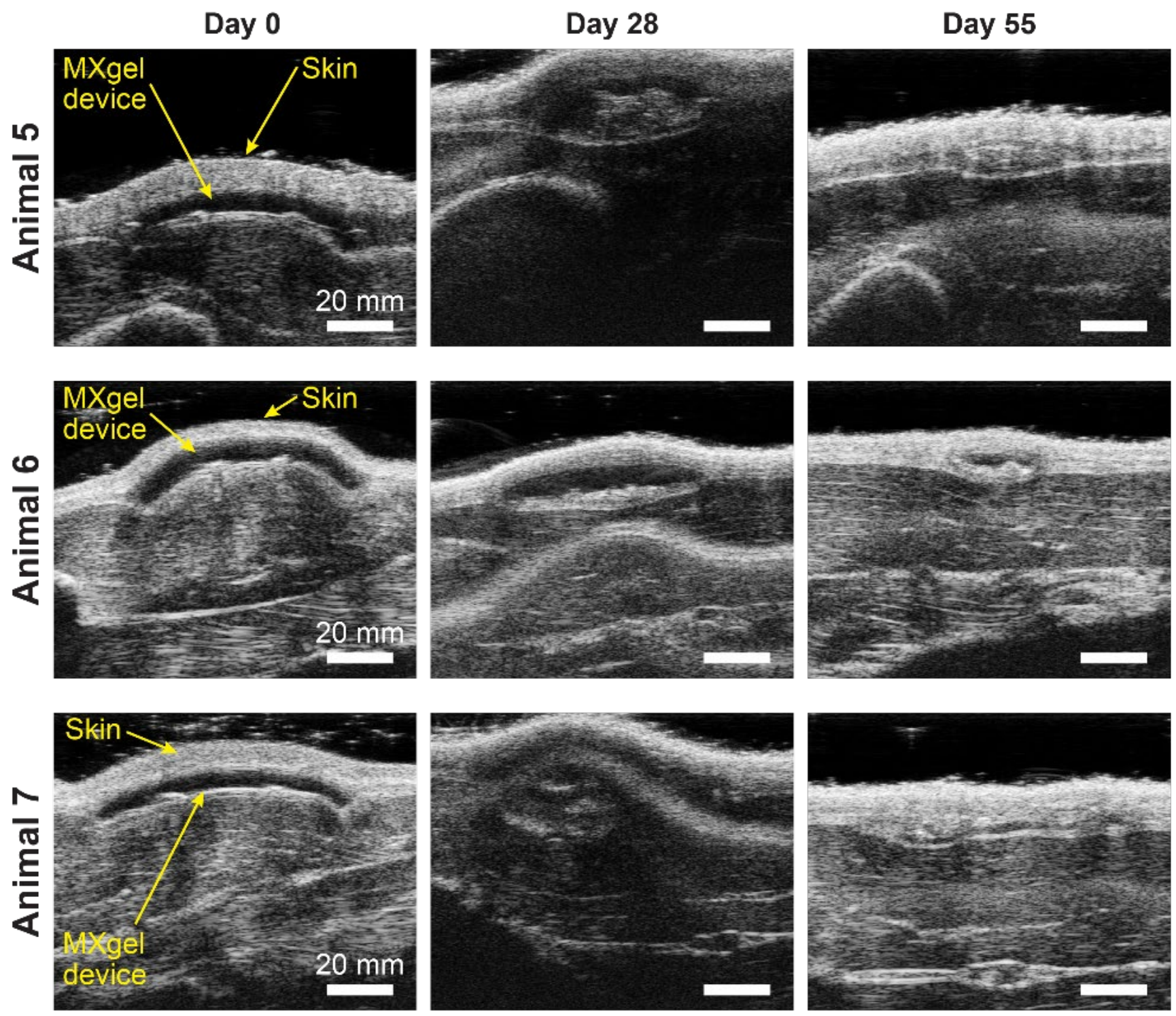

Figure S29. *In vivo* biodegradation of MXgel bioelectronics over 8 weeks. Representative ultrasound images of the *biceps femoris* before and after implantation of MXgel bioelectronics at days 0, 28, and 55 (terminal day) for 3 animals.

Supplementary Tables

Table S1. Elemental composition calculated from EDS maps for Ca^{2+} crosslinked $Ti_3C_2T_x$ MXgels. Data presented as mean ± SD ($N = 3$).

Element	Atomic (%)
Ti	34.3 ± 5.8
C	27.4 ± 4.3
O	17.1 ± 1.3
F	11.1 ± 2.7
Cl	6.6 ± 2.8
Ca	2.9 ± 1.5
Al	0.7 ± 0.5

Table S2. Ti:O ratio calculated from EDS maps of $Ti_3C_2T_x$ MXgels before and after degradation in 1x PBS and 1x PBS with 30 mM H_2O_2.

Condition	Ti : O
Pristine	2 : 1
4 weeks in 1x PBS	1 : 1.8
4 days in PBS + H_2O_2	1 : 3.8

Table S3. Fitting parameters for the conductivity degradation of MXgel samples fabricated with small, medium, and large flakes $Ti_3C_2T_x$ in 1x PBS with 30 mM H_2O_2 at 37°C.

MXene flake size	Time constant τ (hrs.)	R^2
Small	1.09	0.99
Medium	1.27	0.99
Large	1.57	0.99

Table S4. Concentration of Ti^{4+} measured in various tissues of rats with MXgel bioelectronic devices implanted over the *biceps femoris*.

Weeks after implantation	Tissue type	Tissue weight used (mg)	N	Ti^{4+} (ppm)
4	Liver	500	4	Not detected
	Kidneys	500	4	Not detected
	Spleen	200	4	Not detected
8	Liver	500	4	0.02 ± 0.01
	Kidneys	500	4	0.02 ± 0.01
	Spleen	200	4	0.01 ± 0.01

Table S5. Summary of the histopathological scores of *biceps femoris* cross-sections following the implantation of MXgel bioelectronics for 4 and 8 weeks. Ipsilateral (Ipsi.) side contained the hydrogel bioelectronic implant, whereas contralateral (Contra.) side served as the non-implanted internal negative control. (*FBR score = Inflammation + Fibrosis*)

Week	Animal	Side	Inflammation	Fibrosis	FBR score	Muscle injury
4	1	Ipsi.	2	2	4	0
		Contra.	0	0	0	0
	2	Ipsi.	2	3	5	0
		Contra.	0	0	0	0
	3	Ipsi.	2	3	5	0
		Contra.	0	0	0	0
	4	Ipsi.	2	3	5	0
		Contra.	0	0	0	0
8	5	Ipsi.	1	3	4	0
		Contra.	0	0	0	0
	6	Ipsi.	1	3	4	0
		Contra.	0	0	0	0
	7	Ipsi.	1	3	4	0
		Contra.	0	0	0	0
	8	Ipsi.	1	3	4	0
		Contra.	0	0	0	0

Note: 0 – None/Unremarkable; 1 – Minimal; 2 – Mild; 3 – Moderate; and 4 – Severe.

Table S6. Summary of the semi-quantitative scores of the persisting MXgel bioelectronics determined from *biceps femoris* cross-sections following the implantation of MXgel bioelectronics for 4 and 8 weeks. Ipsilateral (Ipsi.) side contained the hydrogel bioelectronic implant, whereas contralateral (Contra.) side served as the non-implanted internal negative control.

Week	Animal	Side	Hydrogel persistence
4	1	Ipsi.	3
		Contra.	0
	2	Ipsi.	3
		Contra.	0
	3	Ipsi.	3
		Contra.	0
	4	Ipsi.	3
		Contra.	0
8	5	Ipsi.	1
		Contra.	0
	6	Ipsi.	1
		Contra.	0
	7	Ipsi.	1
		Contra.	0
	8	Ipsi.	1
		Contra.	0

Note: 0 – No material present; 1 – Remanent fragments only; 2 – Low residual structure; 3 – Moderately retained structure with recognizable components; 4 – Completely intact structure.

Table S7. Summary of functional properties of various thin-film (transient) and hydrogels based bioelectronics.

Material composition	Electrical conductivity (S/m)	Electrochemical impedance at 1 kHz (Ω/mm^2)	Charge storage capacity (mC/cm^2)	Transience	Ref.
**Thin-film bioelectronics**					
Mg	2.15×10^7	N/R	N/R	Y	[3, 4]
Mo	1.91×10^7	N/R	N/R	Y	[4]
W	1.79×10^7	N/R	N/R	Y	[4]
Si	N/R	1.6×10^6	N/R	Y	[5]
Mo/n-type Si	N/R	1.3×10^6	N/R	Y	[6]
**Hydrogel bioelectronics**					
CNT/rGO and alginate	1-40	4.39×10^5	8	-	[7]
PEDOT:PSS and polyurethane	1.1×10^3	6.43×10^4	6	-	[8]
PEDOT:PSS and ionic liquid	4.7×10^3	2.65×10^3	164	-	[9]
PEDOT:AlgS and alginate	7.5×10^{-2}	1.25×10^2	N/R	-	[10]
PEDOT:PSS and HAMA/PEGDA	6×10^2	7.64×10^2	~25 (cathodic)	-	[11]
$Ti_3C_2T_x$ MXene/PEDOT and acrylamide	3.85×10^2	N/R	N/R	-	[12]
AgNFs and alginate	3.5×10^4	N/R	N/R	-	[13]

AgNPs and acrylamide/ alginate	1.4×10^5	N/R	N/R	-	[14]
MXgel	Dry: 4.4×10^3<br>Wet: 8×10^2	3.36×10^2	200 (cathodic)	Y	This work

Note:
Mg – Magnesium; Mo – Molybdenum; W – Tungsten; Si – Silicon; CNT – carbon nanotubes; rGO – reduced graphene oxide; PEDOT – poly(3,4-ethylenedioxythiophene); PSS – polystyrene sulfonate; AlgS – Sulfonated alginate; HAMA – methacrylated hyaluronic acid; PEGDA – poly(ethylene glycol) diacrylate; NFs – nanoflakes; NPs – nanoparticles; N/R – Not reported; and Y – Yes.

References

[1] R. V. Shannon, "A Model of Safe Levels for Electrical Stimulation," *IEEE Transactions on Biomedical Engineering* 39 (1992): 424–426.

[2] S. F. Cogan, K. A. Ludwig, C. G. Welle, P. Takmakov, "Tissue Damage Thresholds During Therapeutic Electrical Stimulation," *Journal of Neural Engineering* 13 (2016): 021001.

[3] S.-W. Hwang, H. Tao, D.-H. Kim, et al., "A Physically Transient Form of Silicon Electronics," *Science* 337 (2012): 1640–1644.

[4] H. Ryu, M. H. Seo, J. A. Rogers, "Bioresorbable Metals for Biomedical Applications: From Mechanical Components to Electronic Devices," *Advanced Healthcare Materials* 10 (2021): 2002236.

[5] K. J. Yu, D. Kuzum, S.-W. Hwang, et al., "Bioresorbable silicon electronics for transient spatiotemporal mapping of electrical activity from the cerebral cortex," *Nature Materials* 15 (2016): 782–791.

[6] M. Cho, J.-K. Han, J. Suh, et al., "Fully Bioresorbable Hybrid Opto-Electronic Neural Implant System for Simultaneous Electrophysiological Recording and Optogenetic Stimulation," *Nature Communications* 15 (2024): 2000.

[7] C. M. Tringides, N. Vachicouras, I. de Lázaro, et al., "Viscoelastic Surface Electrode Arrays to Interface with Viscoelastic Tissues," *Nature Nanotechnology* 16 (2021): 1019–1029.

[8] T. Zhou, H. Yuk, F. Hu, et al., "3D Printable High-Performance Conducting Polymer Hydrogel for All-Hydrogel Bioelectronic Interfaces," *Nature Materials* 22 (2023): 895–902.

[9] Y. Liu, J. Liu, S. Chen, et al., "Soft and Elastic Hydrogel-Based Microelectronics for Localized Low-Voltage Neuromodulation," *Nature Biomedical Engineering* 3 (2019): 58–68.

[10] H. Montazerian, E. Davoodi, C. Wang, et al., "Boosting Hydrogel Conductivity via Water-Dispersible Conducting Polymers for Injectable Bioelectronics," *Nature Communications* 16 (2025): 3755.

[11] L. Shan, Y. Xue, X. Chen, et al., "Mechanically Compliant and Impedance Matching Hydrogel Bioelectronics for Low-Voltage Peripheral Neuromodulation," *Advanced Materials* (2025): e11014.

[12] H. Sun, S. Wang, P. Wang, et al., "Highly Stretchable, Adhesive, and Conductive PEDOT Nanocomposite Hydrogels for High-Performance Flexible Bioelectronics," *Advanced Science* (2025): e13487.

[13] Y. Ohm, C. Pan, M. J. Ford, X. Huang, J. Liao, C. Majidi, "An Electrically Conductive Silver–Polyacrylamide–Alginate Hydrogel Composite for Soft Electronics," *Nature Electronics* 4 (2021): 185–192.

[14] Y. Hui, Y. Yao, Q. Qian, et al., "Three-Dimensional Printing of Soft Hydrogel Electronics," *Nature Electronics* 5 (2022): 893–903.
